# Exploring Intrinsic Disorder in Human Synucleins and Associated Proteins

**DOI:** 10.3390/ijms25158399

**Published:** 2024-08-01

**Authors:** Sriya Reddy Venati, Vladimir N. Uversky

**Affiliations:** 1 Department of Molecular Medicine, Morsani College of Medicine, University of South Florida, Tampa, FL 33612, USA; sriyareddyvenati@usf.edu; 2 USF Health Byrd Alzheimer’s Research Institute, Morsani College of Medicine, University of South Florida, Tampa, FL 33612, USA

**Keywords:** α-synuclein, β-synuclein, γ-synuclein, intrinsically disordered protein, liquid–liquid phase separation, Parkinson’s disease, protein–protein interactions

## Abstract

In this work, we explored the intrinsic disorder status of the three members of the synuclein family of proteins—α-, β-, and γ-synucleins—and showed that although all three human synucleins are highly disordered, the highest levels of disorder are observed in γ-synuclein. Our analysis of the peculiarities of the amino acid sequences and modeled 3D structures of the human synuclein family members revealed that the pathological mutations A30P, E46K, H50Q, A53T, and A53E associated with the early onset of Parkinson’s disease caused some increase in the local disorder propensity of human α-synuclein. A comparative sequence-based analysis of the synuclein proteins from various evolutionary distant species and evaluation of their levels of intrinsic disorder using a set of commonly used bioinformatics tools revealed that, irrespective of their origin, all members of the synuclein family analyzed in this study were predicted to be highly disordered proteins, indicating that their intrinsically disordered nature represents an evolutionary conserved and therefore functionally important feature. A detailed functional disorder analysis of the proteins in the interactomes of the human synuclein family members utilizing a set of commonly used disorder analysis tools showed that the human α-synuclein interactome has relatively higher levels of intrinsic disorder as compared with the interactomes of human β- and γ- synucleins and revealed that, relative to the β- and γ-synuclein interactomes, α-synuclein interactors are involved in a much broader spectrum of highly diversified functional pathways. Although proteins interacting with three human synucleins were characterized by highly diversified functionalities, this analysis also revealed that the interactors of three human synucleins were involved in three common functional pathways, such as the synaptic vesicle cycle, serotonergic synapse, and retrograde endocannabinoid signaling. Taken together, these observations highlight the critical importance of the intrinsic disorder of human synucleins and their interactors in various neuronal processes.

## 1. Introduction

The synuclein family of proteins, comprising α-, β-, and γ-synucleins, plays a critical role in synaptic regulation [1,2]. The proteins of the synuclein family are primarily expressed in vertebrate neuronal tissues, and in humans, they have been found to be associated with various neurodegenerative diseases, such as Parkinson’s disease (PD) [1,2]. All three family members were shown to be mostly disordered in the purified form in vitro [3,4,5,6,7,8,9], and the intrinsically disordered nature of α-synuclein was verified in cellulo [10,11,12,13,14,15,16]. However, at interaction with lipid membranes, the synuclein proteins can undergo disorder-to-order transitions and exhibit an α-helical lipid-bound structure, peculiarities of which have been well-studied due to the analysis of the pathological mutations causing toxicity related to the development of the early onset of PD [1,2].

Of the three synuclein proteins, α-synuclein has been the most studied due to its higher abundance in the brain and because of the discovery of its link to the pathogenesis of PD and later to the development of many other neurodegenerative diseases collectively known as synucleinopathies [17,18,19,20,21,22,23]. In fact, as of 31 March 2024, the Web of Science database contained 30,697 papers dedicated to this protein, a remarkable two-fold increase in comparison with the results of the analogous literature analysis reported in 2017 [1]. The researcher’s strong attention to this protein is determined by its important role in the pathogenesis of neurodegenerative diseases. Although α-synuclein has been originally found to be accumulated in the Lewy bodies (LBs) and Lewy neurites (LNs), which are specific pathological hallmarks in PD cases, later misbehavior of this protein has also been linked to multiple other neurodegenerative diseases, such as Alzheimer’s disease, Down’s syndrome [1], and many other synucleinopathies [17,18,19,20,21,22,23]. In fact, some of the other maladies associated with α-synuclein misbehavior include neurodegeneration with brain iron accumulation type 1 (NBIA1), pure autonomic failure, Down’s syndrome, amyotrophic lateral sclerosis-parkinsonism-dementia complex of Guam (Guam ALS/PDC), multiple system atrophy (MSA), and several LB disorders (that, in fact, might represent a clinical continuum [24]), such as sporadic and familial PD, dementia with Lewy bodies (DLB), diffuse Lewy body disease (DLBD), the Lewy body variant of Alzheimer’s disease (LBVAD), and PD dementia (PDD) [25,26,27,28,29,30,31,32,33].

α-Synuclein aggregation leading to the formation of various oligomers, amorphous aggregates, and amyloid-like fibrils is one of the critical features of this protein, which can be affected by a variety of factors and mechanisms [1,34,35,36]. It was indicated that synucleinopathies represent the α-synuclein-related brain amyloidoses, as selectively vulnerable neurons and glia in different affected brain regions are characterized by the presence of common pathological intracellular inclusions containing α-synuclein, the formation of which correlates with the degeneration of the afflicted brain regions, leading to the onset and progression of the clinical symptoms of these diseases [17,18,23,26,33,37,38,39]. Accumulation of α-synuclein-containing inclusions was detected in the dorsal motor vagal and solitary nuclei, locus coeruleus, parabrachial nuclei, pedunculopontine, and raphe nuclei, periaqueductal gray, prepositus hypoglossal, substantia nigra, reticular formation, and ventral tegmental area, and demonstrated the presence of LN in brainstem fiber tracts and the existence of LBs and LNs in cranial nerve nuclei, premotor oculomotor, precerebellar, and vestibular brainstem nuclei [40,41,42]. Furthermore, the α-synuclein deposition-related pathological processes were shown to spread transneuronally along anatomical pathways [42], supporting the notion of prion-like propagation of the pathological spread within the affected brain during the disease progression (e.g., as described by Braak’s staging criteria for PD [43,44]).

Recent research has also suggested that α-synuclein can form polymorphic structures under certain conditions [1]. Moreover, both the monomeric and polymorphic forms of α-synuclein are amenable to various post-translational modifications (PTMs), providing means for a further increase in the structural and functional diversity of this protein. Furthermore, the capability of α-synuclein to form different high-molecular-weight assemblies was linked to the ability of this protein to trigger different synucleinopathies [45], as demonstrated by the direct observation of the induction of different synucleinopathies after injection of the different α-synuclein aggregated forms (oligomers, ribbons, and fibrils) in the rat brain [46].

Additionally, several pathological mutations of α-synuclein associated with the early onset of PD have been found to increase the aggregation potential of this protein in neurodegenerative diseases [47,48,49,50,51,52,53]. For example, mutation A53T has been found to accelerate fibril formation, thus increasing the chances of inconsistent interactions [1,47]. Another mutation is A30P, which is caused by the replacement of alanine at position 30 by proline. A30P has been found to reduce the binding of α-synuclein to vesicles [47]. Another mutation that has been well studied is E46K, where glutamic acid at position 46 is replaced with lysine [47]. This mutation increases the binding of α-synuclein to liposomes and shows similar effects as A53T. Histidine 50 to glutamine substitution (H50Q) represents another α-synuclein mutation associated with familial PD [54,55]. This mutation was predicted to perturb the same amphipathic α-helix as the previously described pathogenic mutations [55]. It was shown that H50Q was able to enhance the aggregation, secretion, and toxicity of α-synuclein, suggesting that this mutation may play a role in the extracellular toxicity of this protein [56].

Besides its astonishing multipathogeneity, α-synuclein has also been shown to present remarkable multifunctionality, exhibiting a wide range of highly diversified biological functions, ranging from control of the neuronal survival [57], regulation of the neuronal apoptotic response [58], and protection of neurons from various apoptotic stimuli [58], to metal binding [59,60,61,62] and interaction with pesticides and herbicides [63,64,65], to fatty acid binding [57] and interaction with plasma membranes leading to the formation of membrane channels or modification of membrane activity [66], to synaptic vesicle release and trafficking [57] and positive and negative regulation of neurotransmitter release [67], to association with mitochondria causing mitochondrial dysfunction [66], to regulation of various enzymes and transporters [57], to and to promiscuous interaction with hundreds of unrelated proteins and other binding partners [57,68,69,70]. To be able to possess its multifunctionality, the α-synuclein structure is expected to be pliable enough to accommodate such features, and indeed, it expresses itself in the form of an intrinsically disordered protein [1,34,35,36]. Such a diverse set of unrelated functions prompted interest among the researchers in exploring the various interactions of α-synuclein with other proteins and their roles in various degenerative diseases. An interesting question pertaining to the functionality of α-synuclein is the prevalence of intrinsic disorder in its interactome.

In contrast, β-synuclein has been understudied (actually, according to the Web of Science database, as of 31 March 2024, there are 463 papers dedicated to this protein) due to its relative scarcity in the neuronal tissues as compared with α-synuclein, which is estimated to account for up to 1% of the total protein in soluble cytosolic brain fractions [71]. However, β-synuclein is typically co-expressed with α-synuclein and acts as a molecular chaperone to inhibit α-synuclein aggregation [72]. Recent research has also linked β-synuclein to various neurodegenerative diseases, sparking interest in the functions of this protein [72]. β-Synuclein has been found to be critical in the reduction of α-synuclein aggregation-induced toxicity [36,72]. In addition, β-synuclein also regulates synaptic function and dopamine transmission through various structural changes [35].

γ-Synuclein is expressed primarily in the peripheral nervous system, in contrast to α- and β-synucleins [73]. Similar to β-synuclein, γ-synuclein has been relatively understudied due to its lesser abundance as compared with the other members of the synuclein family (as of 31 March 2024, there are 498 papers dedicated to this protein in the Web of Science database). γ-Synuclein has been found to be linked to breast and ovarian cancer [73]. However, specific γ-synuclein mutations have also been found in various neurodegenerative diseases, such as Alzheimer’s, raising speculation regarding their role in the detection and potential treatment of such diseases.

One of the basic premises of modern protein science is the recognition and acceptance of the existence of intrinsically disordered proteins (IDPs) and hybrid proteins with intrinsically disordered regions (IDRs) [74,75,76,77,78], which are abundantly present in nature [75]. These biologically active proteins that do not have unique 3D structures as a whole or in part exist as dynamic conformational ensembles [77,79,80,81,82,83,84], which, at the global level, can be collapsed-disordered (molten globule-like), partially collapsed-disordered (pre-molten globule-like), or extended-disordered (coil-like) [85,86]. In a more general view, IDPs are characterized by a highly dynamic, complex, and mosaic structure with multi-level spatiotemporal heterogeneity, where different parts of a protein can be ordered or disordered to a different degree [87,88]. Since ordered and differently disordered protein regions might have well-defined and specific functions, the spatiotemporal heterogeneity of IDPs/IDRs defines their multifunctionality [89]. Therefore, IDPs/IDRs represent structurally and functionally heterogeneous complex systems that operate within the framework of the protein structure-function continuum model [89,90,91,92,93]. The functional repertoire of IDPs, which are typically engaged in recognition, regulation, signaling, and control of various biological pathways and processes [94,95,96], complements the functions of ordered proteins [97,98,99,100]. The structural flexibility of IDPs/IDRs also determines the variety of ways that can be used to regulate and control their functions [87,101,102,103], with one of the important regulatory means being a variety of post-translational modifications (PTMs) [104,105]. Furthermore, structural pliability and the capability of IDPs/IDRs to be involved in weak multivalent interactions define the broad involvement of these proteins in the biological liquid–liquid phase separation (LLPS) that forms the molecular mechanism of the biogenesis of various membrane-less organelles (MLO) and biomolecular condensates [89,106,107,108]. Finally, many IDPs are involved in various human diseases [57,84,94,97,109,110,111,112,113,114,115,116,117,118,119,120,121,122,123,124,125,126,127].

The intrinsically disordered nature of the synuclein family of proteins and their link to various cellular structures and processes observed in the norm and neurodegenerative diseases prompted research into the synuclein family. The functional and structural diversity of these proteins introduces various challenges in the determination of the complete function of the synuclein family. Moreover, the interactions of these proteins with other proteins, which may or may not be intrinsically disordered, introduce additional challenges in the study of neurodegenerative disease. In this work, we explore and compare the sequence and structure of the human synuclein family with those of species from other classes. We attempt to determine the similarity of the synuclein family across species to aid in establishing the function of the proteins. Further, we also conduct a detailed disorder analysis of the proteins of the human synuclein family. Due to the wide variety of interacting proteins in the interactomes of the synuclein family, we performed a detailed disorder analysis of the interacting proteins exhibiting the highest disorder.

## 2. Results and Discussion

### 2.1. Intrinsic Disorder Status of Members of Human Synuclein Family

**Figure 1:**
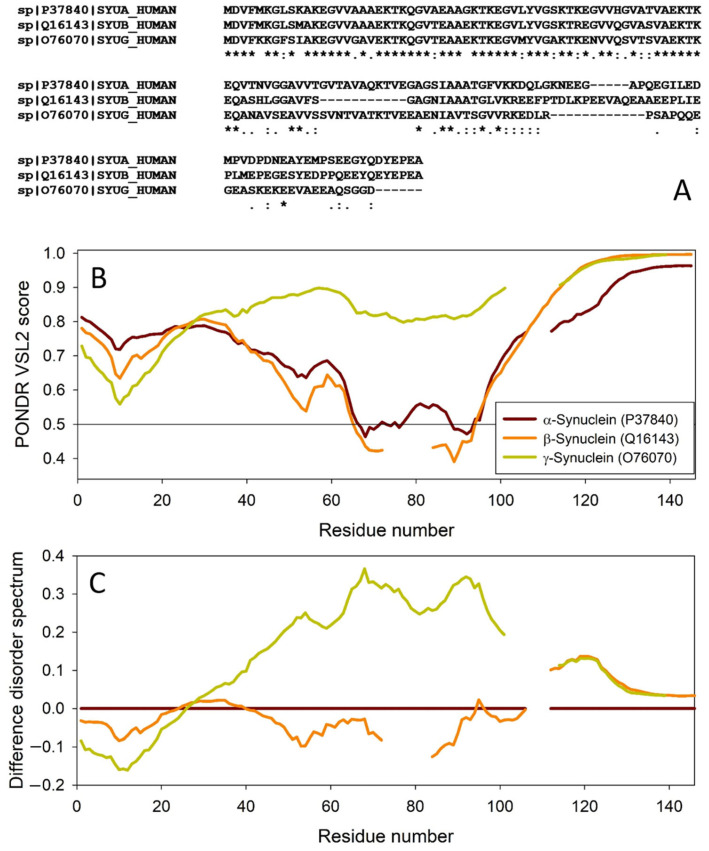
Comparison of amino acid sequences of human α-, β-, and γ-synucleins. (**A**) Multiple sequence alignment conducted by Clustal Omega using default parameters. An asterisk (*) indicates positions which have a single, fully conserved residue. A colon (:) indicates conservation between groups of strongly similar properties and shows that some sequences in a column have different amino acids, but the amino acids have similar chemical properties. A period (.) indicates conservation between groups of weakly similar properties. A dash (-) indicates a gap in the alignment. (**B**) Per-residue disorder profiles of human α-, β-, and γ-synucleins generated by PONDR^®^ VSL2. To better represent the peculiarities of the per-residue intrinsic disorder propensity distribution, the scale of Y–axis is extended to cover PONDR^®^ VSL2 scores from 0.34 to 1.0. (**C**) Difference disorder spectra calculated by subtracting profiles of β- and γ-synucleins from the profiles of human α-synuclein.

**Figure 2:**
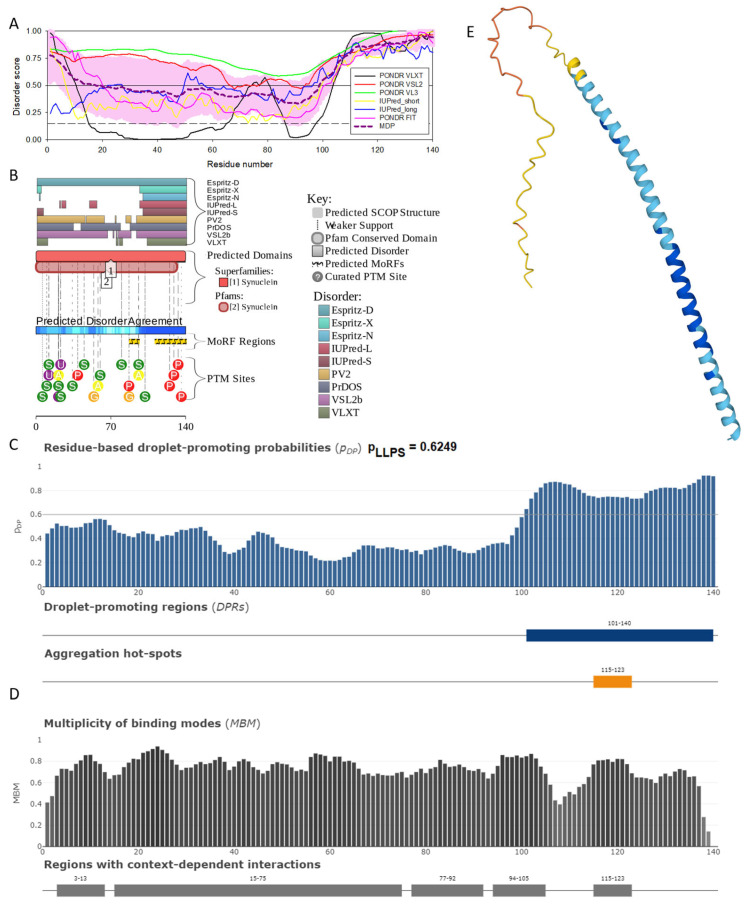
Functional disorder analysis of human α-synuclein (UniProt ID: P37840). (**A**), Multiparametric disorder analysis of the protein using RIDAO. The outputs of PONDR^®^ VLXT, PONDR^®^ VSL2, PONDR^®^ VL3, PONDR^®^ FIT, IUPred long, and IUPred short are shown by black, red, green, pink, blue, and yellow lines, respectively. Mean disorder profile (or mean disorder prediction, MDP), calculated as an average of outputs of these six predictors, is shown by dashed dark pink line, whereas error distribution is shown as light pink shadow. In this per-residue disorder analysis, a disorder score was assigned to each residue. A residue with disorder score equal to or above 0.5 is considered disordered, and a residue with disorder score below 0.5 is predicted to be ordered. Residues/regions with disorder scores between 0.15 and 0.5 were considered ordered but flexible. The corresponding thresholds are shown by solid (0.5) and long-dashed lines (0.15). (**B**) Functional disorder profile generated for α-synuclein by the D^2^P^2^ database showing the outputs of several disorder predictors such as VLXT, VSL2b, PrDOS, IUPred, and Espritz. The colored bar highlighted by blue and green shades represents the disorder prediction; yellow zigzagged bars show positions of MoRFs, whereas colored circles at the bottom of the plot show the positions of predicted PTMs, such as phosphorylation (red circles marked P), sumoylation (green circles marked S), acetylation (yellow circles marked A), glycosylation (orange circles marked G), and ubiquitylation (violet circles marked U). (**C**) The FuzDrop-generated plot shows the sequence distribution of the residue-based droplet-promoting probabilities, pDP, for human α-synuclein. (**D**) The FuzDrop-generated plot of the multiplicity of binding modes shows positions of regions that can sample multiple binding modes in a cellular context (sub-cellular localization, partners, posttranslational modifications)-dependent manner. (**E**) 3D structural model as predicted by AlphaFold. The structure is colored according to the per-residue model confidence score (p_LDDT_) ranging from orange to blue, where fragments of structure with very high (p_LDDT_ > 90), confident (90 > p_LDDT_ > 70), low (70 > p_LDDT_ > 50), and very low (p_LDDT_ < 50) p_LDDT_ scores are shown by blue, cyan, yellow, and orange colors, respectively.

**Figure 3:**
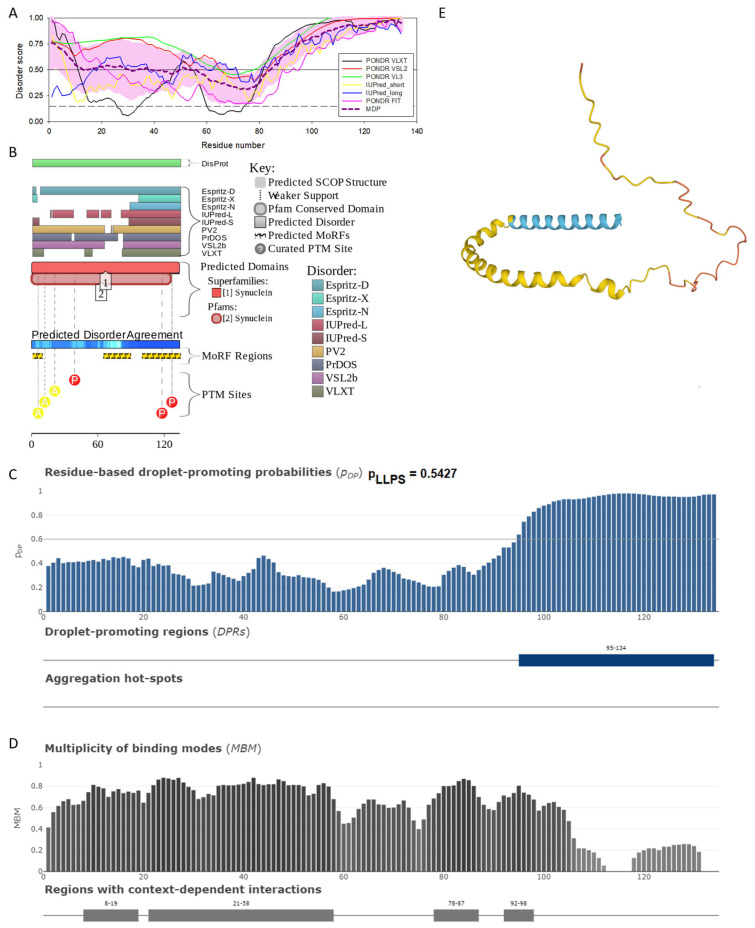
Functional disorder analysis of human β-synuclein (UniProt ID: Q16143). (**A**), Multiparametric disorder analysis of the protein using RIDAO. (**B**) Functional disorder profile generated for human β-synuclein by the D^2^P^2^ database. Colored circles at the bottom of the plot show the localization of PTMs, such as phosphorylation (red circles marked P) and acetylation (yellow circles marked A). (**C**) The FuzDrop-generated plot shows the sequence distribution of the residue-based droplet-promoting probabilities, pDP. (**D**) The FuzDrop-generated plot of the multiplicity of binding modes. (**E**) A 3D structural model is predicted by AlphaFold.

**Figure 4:**
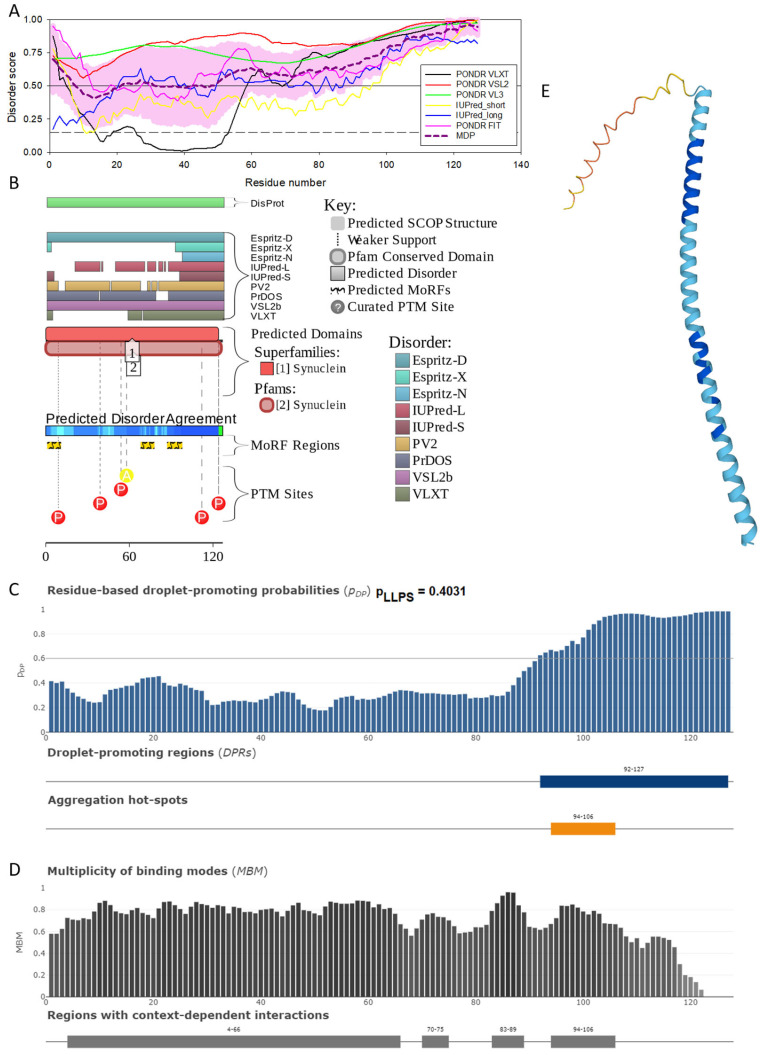
Functional disorder analysis of human γ-synuclein (UniProt ID: O76070). (**A**), Multiparametric disorder analysis of the protein using RIDAO. (**B**) Functional disorder profile generated for human γ-synuclein by the D^2^P^2^ database. Colored circles at the bottom of the plot show the localization of PTMs, such as phosphorylation (red circles marked P) and acetylation (yellow circles marked A). (**C**) The FuzDrop-generated plot shows the sequence distribution of the residue-based droplet-promoting probabilities, pDP. (**D**) The FuzDrop-generated plot of the multiplicity of binding modes. (**E**) A 3D structural model is predicted through AlphaFold.

The amino acid sequences of all the synucleins analyzed in this study are listed in Supplementary Table S1. Figure 1, Figure 2, Figure 3 and Figure 4 represent the results of the intrinsic disorder-centric analysis of human α-, β-, and γ-synucleins, which consist of 140, 134, and 127 amino acids, respectively. It was emphasized that among the characteristic features of human synucleins is the presence of acidic stretches within their C-terminal regions, whereas within their 87 N-terminal residues, they possess a degenerative KTKEGV repeat that defines the hydrophobic variability of their sequences with a periodicity of 11 amino acids, which is characteristic of the amphipathic helices [128]. To illustrate the sequence similarity of the members of the human synuclein family, Figure 1A represents the results of the multiple sequence alignment of these proteins. Although human α- and β-synucleins share 78% identical residues, including conserved C-termini containing three identically placed tyrosine residues, β-synuclein lacks 11 residues (residues 73–83) within its middle region [19]. There is 60% sequence similarity between human α- and γ-synucleins, with γ-synuclein lacking the tyrosine-rich C-terminal signature of α- and β-synucleins [19]. The results of multiple sequence alignment were combined with the outputs of the PONDR^®^ VSL2-based per-residue disorder analysis to generate the aligned disorder profiles of human synucleins. Figure 1B shows that all three proteins are mostly disordered. To better illustrate differences in the disorder propensity of these proteins, we generated their “difference disorder spectra” by subtracting the human α-synuclein per-residue disorder propensities from the corresponding data for the β- and γ-synucleins (see Figure 1C). The use of this approach highlights the local differences in the disorder propensity, as positive peaks in the resulting plots show regions in β- and γ-synucleins with an increased local disorder propensity relative to the human α-synuclein. On the other hand, negative peaks correspond to regions with decreased disorder propensity. Therefore, Figure 1C clearly indicates that β-synuclein is moderately less disordered than α-synuclein (with the noticeable exception of the 35 residues in its C-terminal region), whereas γ-synuclein is noticeably more disordered than both other synucleins almost over its entire length (with the exception of the 25 N-terminal residues).

The analysis of these figures provides compelling evidence of the highly disordered nature of all three members of the human synuclein family. Originally, the interest of the researchers in human α-synuclein was promoted by finding a relation between the aggregation of this protein and the pathogenesis of Parkinson’s disease (PD), which is recognized as the most common aging-related movement disorder and the second most common neurodegenerative disease after Alzheimer’s disease (AD). It is estimated that ~1.5 million Americans are affected by PD. Sporadic (or idiopathic) forms of this disease account for about 95% of PD patients [129,130]. The probability of sporadic PD development increases with age, with only a small percentage of patients diagnosed before the age of 50 [131]. The prevalence of PD is much greater among those who are at least 65 years old [132]. Approximately 1% of the population at 65–70 years of age is affected by PD, whereas the number of PD patients increases to 4–5% in 85-year-olds [133]. In addition to the sporadic form, multiple familial forms of PD are associated with mutations in a number of genes. These hereditary forms account for ~4% of PD patients who develop early-onset disease before the age of 50 [134,135]. The pathological hallmarks of PD are the presence of cytosolic filamentous inclusions known as Lewy bodies (LBs) and Lewy neurites (LNs) in surviving dopaminergic neurons within the substantia nigra [8,9]. These inclusions that contain aggregated forms of α-synuclein can also be found in other parts of the brain [136] and are associated with the pathogenesis of various synucleinopathies [25,26,27,28,29,30,31,32,33], characterized by the presence of the common pathologic inclusions composed of aggregated α-synuclein, which are deposited in selectively vulnerable neurons and glia [17,18,23,38]. Finding α-synuclein in LBs and LNs [32,37], as well as the existence of the specific missense mutations in the SNCA gene, corresponding to the A30P, E46K, and A53T substitutions in the α-synuclein protein in autosomal dominant early-onset forms of PD [137,138,139], and a link of other early-onset PD forms to the hyper-expression of wild type α-synuclein due to the gene duplication/triplication [140,141,142] strongly implicated α-synuclein in the PD pathogenesis.

The α-synuclein sequence is assumed to contain three functional regions: the N-terminal region (residues 1–60) contains four 11-amino acid imperfect repeats with a conserved motif (KTKEGV, residues 10–15, 21–26, 32–37, and 43–48); the central region (residues 61–95) that contains three additional repeats (residues 58–63, 69–74, and 80–85) and is known as a highly amyloidogenic non-Aβ component of AD plagues (NAC) region that was found in amyloid plaques associated with AD [118]; and the highly charged C-terminal region (residues 96–140) which is involved in protein–protein interactions. Note that the N-terminal and central regions comprise a lipid-binding domain. A detailed experimental analysis of purified α-synuclein in vitro provided strong evidence of the highly disordered nature of this protein [3,4,6,143]. However, it was also indicated that the structure of α-synuclein does not represent a random coil but is characterized by the presence of transient long-range contacts within the protein [9,144,145,146].

In agreement with experimental data, Figure 2A,B show that human α-synuclein is predicted to be highly disordered by most computational tools utilized in this study. Furthermore, Figure 2B shows that the C-terminal region of this protein contains two molecular recognition features (MoRFs, which are disordered regions that can undergo binding-induced folding at interaction with specific partners) (residues 87–94 and 111–140), and the entire protein is heavily decorated by multiple PTMs (which are commonly located within intrinsically disordered regions, IDRs), clearly indicating the crucial functional role of its intrinsic disorder. Figure 2C shows that human α-synuclein is characterized by a high liquid–liquid phase separation (LLPS) potential. Its probability of spontaneous liquid–liquid phase separation (p_LLPS_) value of 0.6249 exceeds the threshold of 0.6, indicating that the α-synuclein can act as a droplet-driver capable of undergoing LLPS spontaneously [147]. Furthermore, the C-terminal region of this protein contains a long droplet-promoting region (DPR, residues 101–140), which also includes an aggregation hotspot (residues 115–123), which is defined as a region that is capable of promoting the conversion of the liquid-like condensed state into a solid-like amyloid state [148]. These predicted LLPS potentials of human α-synuclein are in line with the experimentally demonstrated capability of this protein to undergo LLPS [149,150,151,152,153].

**Figure 5:**
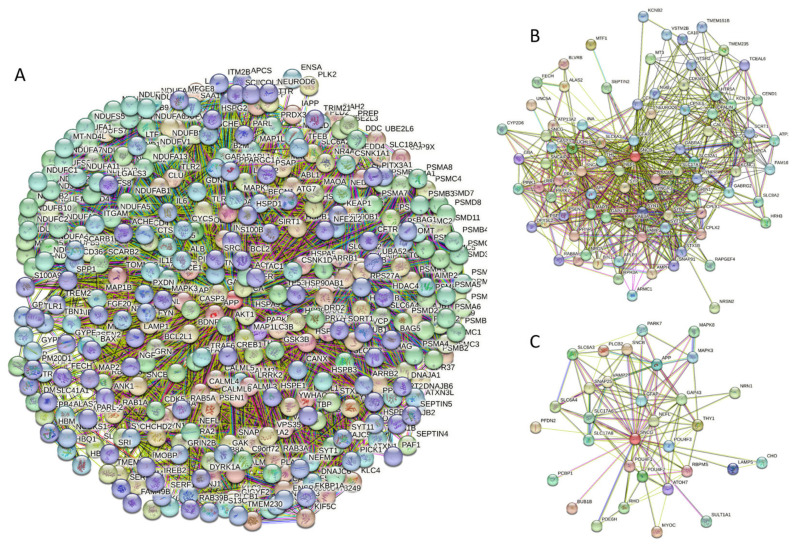
(**A**) Protein–protein interaction network of human α-synuclein (UniProt ID: P37840) (**A**), β-synuclein (UniProt ID: Q16143) (**B**), and human γ-synuclein (UniProt ID: O76070) (**C**). These PPI networks were generated by STRING using the minimum required interaction score of 0.5 (α-synuclein) or 0.4 (medium confidence, β- and γ-synucleins) and adjusting the value of the maximum number of interactors in the first shell to 500. Network nodes represent individual proteins, and edges represent protein–protein interaction for shared function, with different types of interactions; the blue line represents curated databases, black line represents co-expression, and green line represents gene neighborhoods. Access to the interactive PPI maps for α-, β-, and γ-synucleins can be found on the STRING webpage via the following URLs: https://string-db.org/cgi/network?taskId=bTG0CScAf8Wp&sessionId=bcRTSNZudCtN (accessed on 5 May 2024); https://string-db.org/cgi/network?taskId=bvfk903ldEwq&sessionId=bcRTSNZudCtN (accessed on 5 May 2024), and https://string-db.org/cgi/network?taskId=brXAj9n6xxUM&sessionId=bcRTSNZudCtN (accessed on 5 May 2024).

Curiously, Figure 2D shows that human α-synuclein is expected to contain multiple regions with context-dependent interactions (residues 3–13, 15–75, 77–92, 94–105, and 115–123), i.e., regions exhibiting ordered or disordered binding modes depending on the cellular context (environment, sub-cellular localization, partners, and PTMs). These regions are capable of engaging in a multiplicity of binding modes in a cellular context-dependent manner [154]. The data shown in Figure 2B,D indicate that human α-synuclein is predisposed to be a promiscuous binder, as its almost entire sequence can act as a potential binding platform. In line with this conjecture, Figure 5A shows that α-synuclein can be engaged in interaction with 356 proteins, forming a very dense protein–protein interaction network, 357 members of which are connected by 7316 interactions. This network is characterized by an average node degree of 41 and an average local clustering coefficient of 0.639. Since the expected number of edges in a random set of proteins of the same size and degree distribution drawn from the genome is 2946, this α-synuclein-centric network has significantly more interactions than what would be expected (its PPI enrichment *p*-value is <1.0 × 10^−16^). The five most enriched biological processes, molecular functions, and cellular components (as per Gene Ontology annotations) of the members of this network, as well as the most enriched local STRING network clusters and KEGG pathways, are listed in Table 1.

Figure 2E demonstrates the 3D structure of human α-synuclein modeled by AlphaFold. According to this model, α-synuclein does not have a compact core, with the only structured element predicted in this protein being a long α-helix spanning residues 1–91. This is a rather unrealistic structure, as long α-helices typically cannot exist in isolation, as they need to be stabilized by interactions either with the compact protein core or via binding to specific partners, such as other proteins, nucleic acids, or membranes. Therefore, it is likely that in this case, AlphaFold predicts the 3D structure of a bound form of α-synuclein. In fact, comprehensive experimental analysis of purified α-synuclein in vitro using a multitude of techniques sensitive to different levels of protein structural organization revealed that this protein is highly disordered [3,4,6,143]. Although transient long-range interactions were observed within this protein [9,144,145,146] solution, NMR analysis did not show the presence of any stable structural elements in the unbound form of this protein. However, this protein has been shown to adopt a secondary structure of mostly helical nature upon interaction with negatively charged small, unilamellar vesicles (SUVs) or detergent micelle surfaces [3,5,155,156], and α-helical structure was induced in this protein in the presence of lipids [157] and organic solvents [158]. Furthermore, binding of α-synuclein to a micelle of the detergent sodium lauroyl sarcosinate (SLAS) was shown to be accompanied by the disorder-to-order transition resulting in the formation of two antiparallel micelle-bound α-helices (residues 1–31 and 41–91) [159]. In agreement with this NMR-EPR-based study, solution NMR analysis of the micelle-bound form of α-synuclein revealed the presence of the two anti-parallel curved α-helices (residues 3–37 and 45–92) connected via an extended but well-ordered linker [160].

Similar to α-synuclein, human β-synuclein is predicted to contain high levels of intrinsic disorder (see Figure 3). The major difference between these two proteins is the lack of 11 residues (residues 73–83) within the middle region of β-synuclein [19]. As a result, the overall percent of disordered residues (as per PONDR^®^ VSL2 analysis) decreases from 90.71% in α-synuclein to 87.31% in β-synuclein. On the contrary, the average prediction score increased from 0.7199 in α-synuclein to 0.7342 in β-synuclein (see Figure 3A). Figure 3B shows that human β-synuclein, being predicted to be mostly disordered by all the tools included in the D^2^P^2^-based analysis, is expected to have three MoRFs (residues 1–9, 65–89, and 100–134), indicating that intrinsic disorder plays a crucial role in its interactability. Furthermore, the function of β-synuclein can be modulated by various PTMs. At the same time, this protein has lost the capability to undergo spontaneous LLPS (its p_LLPS_ of 0.5427 is below the threshold of 0.6) together with the aggregation hot spot. However, β-synuclein can still act as a droplet client since it has a long DPR (residues 95–134) at its C-terminal tail (see Figure 3C). As per Figure 3D, human β-synuclein contains four regions with context-dependent interactions (residues 8–19, 21–58, 78–87, and 92–98). Therefore, this protein is also expected to act as a highly promiscuous binder. The idea is supported by Figure 5B, which shows the β-synuclein-centered PPI network generated by STRING, which contains 85 nodes connected by 715 edges. The average node degree of this network is 16.8, and its average local clustering coefficient is 0.682. Furthermore, this network has significantly more interactions than expected (715 vs. 143), as characterized by the PPI enrichment *p*-value of <1.0 × 10^−16^. The five most enriched biological processes, molecular functions, and cellular components (as per Gene Ontology annotations) of the members of this network, as well as the most enriched local STRING network clusters and KEGG pathways, are listed in Table 1. Among the functional differences among the members of the α-synuclein- and β-synuclein-centered PPI networks is a remarkable change in the KEGG pathways from exclusively disease-oriented pathways in the α-synuclein-centered network (PD, ALS, AD, Prion disease, and Huntington’s disease) to the synaptic vesicle cycle, PD, nicotine addiction, serotonergic synapse, and insulin secretion pathways in the β-synuclein-centered PPI network.

Similar to α-synuclein, human β-synuclein was shown experimentally to be extensively disordered [6,8,9,72], with β-synuclein being somewhat more disordered than α-synuclein [6]. These experimental observations are supported by the results of our computational analysis. Figure 3E represents the AlphaFold-generated 3D structural model of human β-synuclein, showing the presence of a single, long, horseshoe-like α-helix (residues 2–80). Solution NMR analysis of this protein in its unbound form revealed that its residual structure was shown to noticeably differ from that of α-synuclein, with the helical propensity of β-synuclein being clearly reduced between residues 66 and 83 [9]. This difference in the residual structure of the unbound state was shown to propagate to its micelle-bound form, as the NMR analysis revealed that although the lipid-binding domain of β-synuclein, which is missing 11 residues, remains predominantly helical in the micelle-bound form and preserves the break around position 42, it is characterized by a dramatic decrease in the stability of the helical structure within the 65–83 region [8].

Figure 4 shows that human γ-synuclein (which is different from other members of the human synuclein family by the absence of the tyrosine-rich C-terminal signature [19]) is also predicted to be a highly disordered protein. In fact, it seems that it is the most disordered member of the family, since its overall percent disordered residues (as per PONDR^®^ VSL2 analysis) is 100% and its average prediction score is 0.8328 (see Figure 4A). Figure 4B represents the functional disorder profile of human γ-synuclein generated by the D^2^P^2^ platform and also shows the high prevalence of disorder in this protein, which is also expected to have three MoRFs (residues 1–10, 68–77, and 87–97) and several PTMs. As per FuzDrop analysis (see Figure 4C), γ-synuclein is not expected to undergo spontaneous LLPS but can serve as a droplet client and also contains an aggregation hotspot (residues 94–106). These features make this protein closer to α-synuclein than to β-synuclein. This hypothesis is supported by experimental analyses that revealed the closer structural similarity of these two proteins [6,9,161]. The decreased aggregation potential of γ-synuclein in comparison with that of α-synuclein was attributed to an increased α-helical propensity in the amyloid-forming region that is critical for α-synuclein fibrillation, suggesting that increased structural stability in this region may protect against γ-synuclein aggregation [161]. Figure 4D shows the presence of four regions with context-dependent interactions (residues 4–66, 70–75, 83–89, and 94–106). Two of these regions overlap with MoRFs. Figure 5C represents the γ-synuclein-centered PPI network, which contains 32 nodes and 117 edges. Although this network is the smallest one among the synuclein family members, it still has significantly more interactions than expected (117 vs. 46). It is characterized by a PPI enrichment *p*-value of <1.0 × 10^−16^, an average node degree of 7.31, and a high average local clustering coefficient of 0.752. The five most enriched biological processes, molecular functions, and cellular components (as per Gene Ontology annotations) of the members of this network, as well as the most enriched local STRING network clusters and KEGG pathways, are listed in Table 1. Finally, Figure 4E represents a 3D model of human γ-synuclein generated by AlphaFold. In line with all other data discussed in this section, this structural model is very similar to that generated for α-synuclein, where a single long α-helix (residues 2–91) is observed.

**Figure 6:**
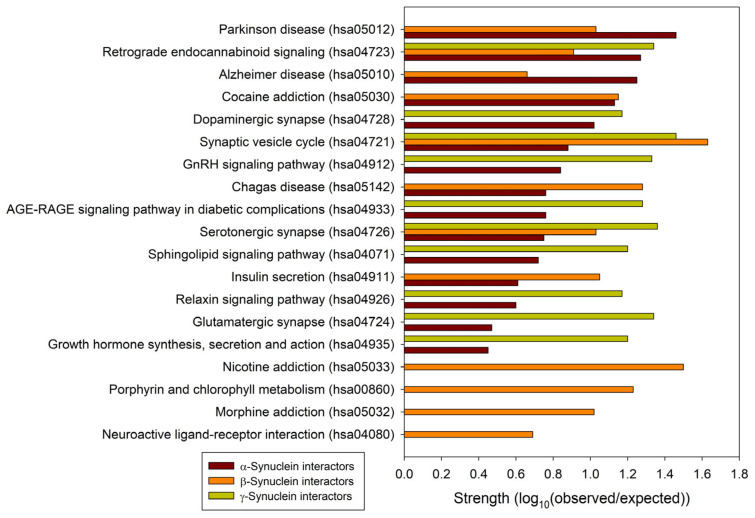
Comparison of the functional enrichments of the interactomes of human synucleins in terms of the abundance of the corresponding proteins in various KEGG pathways. Strength corresponds to Log_10_(observed/expected), a measure describing the scale of the enrichment effect. The ratio considered here is between the number of proteins in the given STRING-generated network that are annotated with a given term and the number of proteins that are expected to be annotated with this term in a random network of the same size.

To understand the general similarity and difference in the functionality of proteins interacting with human α-, β-, and γ-synucleins, we conducted a comparative analysis of the functional enrichment of the members of the corresponding PPI networks. To this end, we looked at the abundance of these proteins in Kyoto Encyclopedia of Genes and Genome (KEGG) pathways [162,163]. Based on the analysis of the networks generated by STRING using the parameters utilized in this study, α-synuclein interactors were found to be associated with 158 different KEGG pathways. These findings are summarized in Supplementary Table S2. A detailed description of these pathways is outside the scope of this study. However, it is important to mention that via its interactors, α-synuclein is involved in numerous pathological pathways, including those associated with neurodegenerative diseases such as Alzheimer disease, amyotrophic lateral sclerosis, Huntington disease, Parkinson disease, Prion disease, and Spinocerebellar ataxia, as well as various types of cancer and metabolic diseases (see Supplementary Table S2). On the other hand, interactors in the β- and γ-synuclein-centered PPI networks were associated with 11 KEGG pathways each. These observations indicate that, relative to the β- and γ-synuclein interactomes, α-synuclein interactors are involved in a much broader spectrum of highly diversified functional pathways. One cannot exclude the possibility that this observation could be related to the fact that there are much more studies dedicated to α-synuclein than to two other members of this protein family. Although one would expect that the α-synuclein interactors should be involved in most of the functions conducted by the members of the β- and γ-synuclein interactomes, Figure 6 shows that there are only three common KEGG pathways shared by the interactors of three human synucleins: synaptic vesicle cycle (hsa04721), serotonergic synapse (hsa04726), and retrograde endocannabinoid signaling (hsa04723). On the other hand, α- and β-synucleins have 8 common pathways, whereas interactors of β- and γ-synucleins share 3 KEGG pathways. Furthermore, via their interactors, β-synucleins are associated with several unique KEGG pathways, such as porphyrin and chlorophyll metabolism (hsa00860), nicotine addiction (hsa05033), neuroactive ligand-receptor interaction (hsa04080), and morphine addiction (hsa05032). However, no such unique pathways were found for the γ-synuclein interactome.

### 2.2. Effect of Familial Point Mutations on the Intrinsic Disorder Propensity of Human α-Synuclein

**Figure 7:**
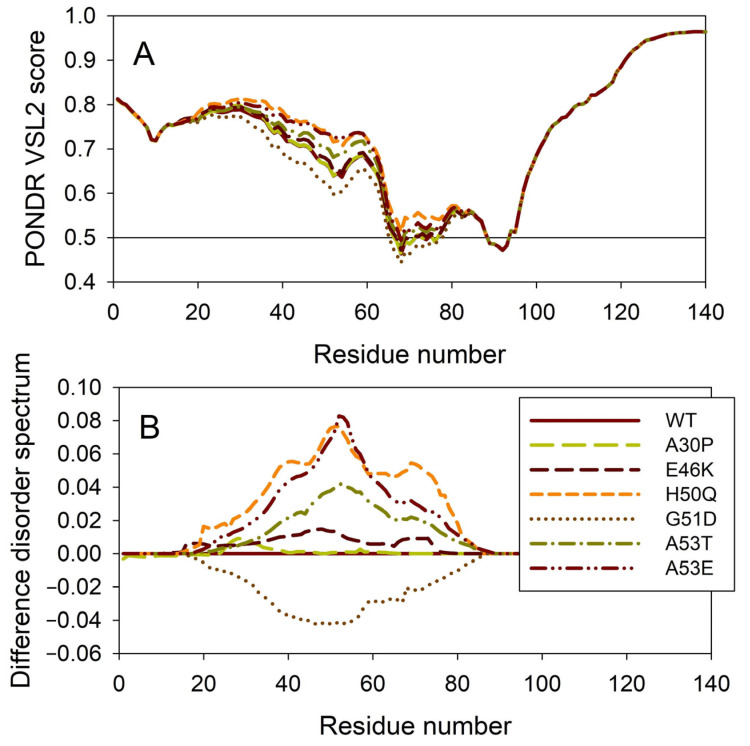
Effect of the missense point mutations associated with the familial cases of PD (A30P, E46K, H50Q, G51D, A53T, and A53E) on the intrinsic disorder propensity of human α-synuclein. (**A**) Per-residue disorder profiles generated by PONDR^®^ VSL2. (**B**) Difference disorder spectra calculated by subtracting mutant profiles from those of the wild type protein.

It is known that the residual structure of α-synuclein is affected by the familial PD missense mutations. There are at least six such mutations: A53T [138], A30P [164], E46K [165], H50Q [54,55], G51D [166,167], and A53E [168]. To understand how these point mutations associated with the early-onset familial cases of PD affect the propensity of α-synuclein for intrinsic disorder, we analyzed the corresponding sequences of the wild type protein (WT) as well as the A30P, E46K, H50Q, G51D, A53T, and A53E mutants using PONDR^®^ VSL2. Results of this analysis are shown in Figure 7A, whereas Figure 7B represents the “difference disorder spectra” calculated by subtracting the wild type per-residue disorder propensities from the corresponding data for the mutants. The use of “difference disorder spectra” simplifies the understanding of the effects of mutations, as positive (or negative) peaks in these plots show regions in mutant proteins with an increased (or decreased) local disorder propensity relative to the wild type protein. Since, with the exception of G51D, all “difference disorder spectra” contain positive peaks, the disease-associated mutations A30P, E46K, H50Q, A53T, and A53E caused some increase in the local disorder propensity. On the other hand, local intrinsic disorder propensity is absent in the G51D mutant. Note that the observed effects are mostly local and small (in a range from 0.01 for A30P and E46K to ~0.08 for H50Q and A53E). Since for estimation of the per-residue disorder scores, the disorder predictors use sliding windows, it is expected that changes in the disorder propensity would propagate outside the mutation site and affect a region containing the analyzed point mutation. The length of a region that “feels” mutation would depend, among other factors, on a window size utilized by the predictor and on the actual scale of the disorder score change at the mutation site. This is illustrated by the comparison of the “difference disorder spectra” generated for A53T and A53E mutants, with the A53T “spectrum” being narrower and less intensive than the A53E “difference disorder spectrum”.

**Figure 8:**
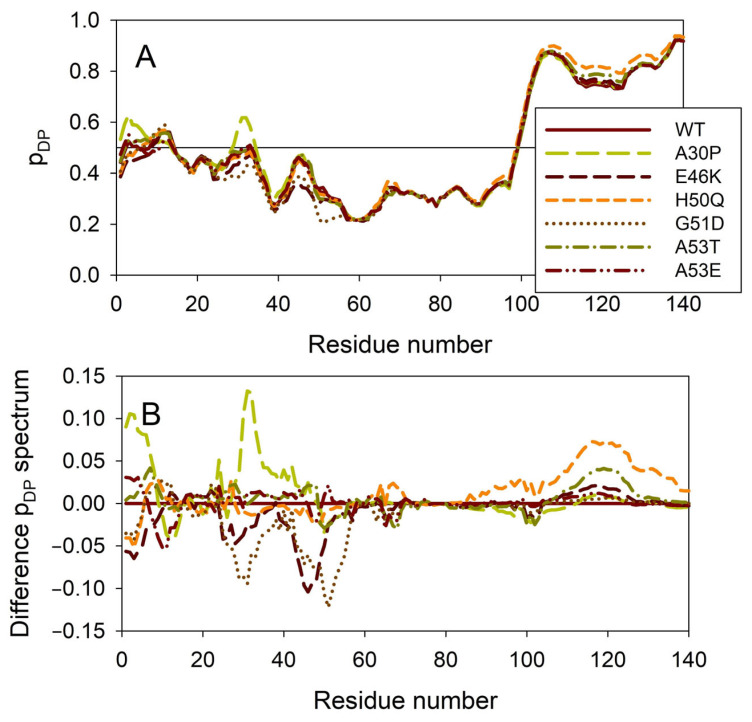
Effect of the missense point mutations associated with the familial cases of PD (A30P, E46K, H50Q, G51D, A53T, and A53E) on intrinsic disorder propensity of human α-synuclein. (**A**) Per-residue droplet-promoting probabilities (p_DP_) evaluated by FuzDrop. (**B**) Difference p_DP_ spectra calculated by subtracting mutant profiles from those of the wild type protein.

Figure 8 illustrates the effect of these mutations on the propensity of human α-synuclein for spontaneous LLPS. Although the droplet-promoting region is located within the C-terminal region of this protein and although all the mutations are located within the N-terminal region, the A30P, E46K, H50Q, G51D, A53T, and A53E mutations show noticeable effects on the LLPS potential of this protein. In fact, based on their propensity for spontaneous liquid–liquid phase separation, p_LLPS_, these forms of α-synuclein can be arranged in the following order: A53T (P_LLPS_ = 0.6416) > A30P (P_LLPS_ = 0.6413) > A53E (P_LLPS_ = 0.6350) > WT (P_LLPS_ = 0.6249) > H50Q (P_LLPS_ = 0.6165) > E46K (P_LLPS_ = 0.5730) > G51D (P_LLPS_ = 0.5153). Based on these observations, one can hypothesize that the capability of α-synuclein to undergo spontaneous LLPS can be eliminated by point mutations E46K and G51D. Since the formation of LLPS is considered a step preceding fibril formation, these data indicate that the aggregation potential of α-synuclein is modulated by mutations. In agreement with these suppositions, these mutations associated with the early onset of PD were experimentally shown to differently modulate α-synuclein functions and aggregation propensity. The A30P mutation promoted the fast formation of non-fibrillar aggregates (such as oligomers or protofibrils) and not fibrils [48,169]. Two other PD mutants, A53T and E46K, were characterized by accelerated fibrillation [48,49,170,171]. Similarly, α-synuclein aggregation and fibrillation were dramatically accelerated by the H50Q mutant [56]. On the other hand, a significant reduction in the α-synuclein oligomerization and fibrillation rates was induced by the G51D and A53E mutations, with the G51D mutant forming amorphous aggregates [167,172] and the A53E mutant being able to slowly form very thin amyloid fibrils [172,173,174].

### 2.3. Intrinsic Disorder Potential of α-, β-, and γ-Synucleins from Other Species

**Figure 9:**
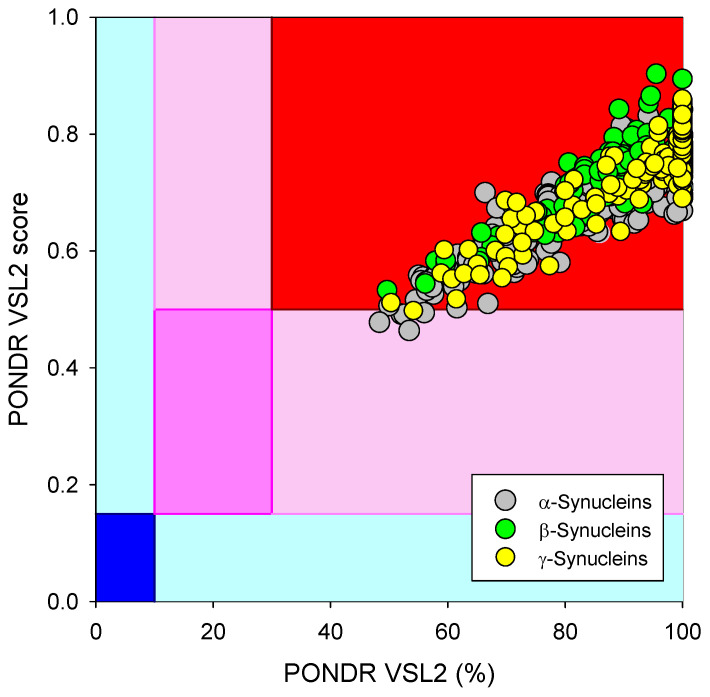
Global disorder analysis of 381 α- synucleins, 320 β- synucleins, and 234 γ-synucleins from different species in the form of the PONDR^®^ VSL2 score vs. PONDR^®^ VSL2 (%) plot. Here, each point corresponds to a query protein, coordinates of which are evaluated from the corresponding PONDR^®^ VSL2 data as its ADS and PPIDR. Color blocks are used to visualize proteins based on the accepted classification, with red, pink/light pink, and blue/light blue regions containing highly disordered, moderately disordered, and ordered proteins, respectively (see the text). Dark blue or pink regions correspond to the regions where PPIDR agrees with ADS, whereas areas in which only one of these criteria applies are shown by light blue or light pink.

Based on the experimental and computational data, all three human synucleins are known as highly disordered proteins, so we decided to evaluate the intrinsic disorder propensities of α-, β-, and γ-synucleins from other species. At the first step, we extracted the amino sequences of 381 α- synucleins, 320 β- synucleins, and 234 γ-synucleins from UniProt and checked their global intrinsically disordered predispositions. The results of these analyses are summarized in Figure 9, which shows the PONDR^®^ VSL2 score vs. PONDR^®^ VSL2 (%) plot for all these proteins. Typically, the percent of the predicted intrinsically disordered residues (PPIDR) is used to classify proteins as highly ordered, moderately disordered, or highly disordered if their corresponding PPIDR values are below 10%, between 10% and 30%, or above 30%, respectively [175,176]. Additional angle is provided by the analysis of the averaged disorder scores (ADS), which are calculated for each query protein as a protein length-normalized sum of all the per-residue disorder scores and classify them as highly ordered, moderately disordered/flexible, or highly disordered if their ADS < 0.15, 0.15 ≤ ADS < 0.5, and ADS ≥ 0.5. Based on these criteria, all synucleins analyzed in this study are clearly classified as highly disordered, being characterized by PPIDR values of 85.8 ± 14.0, 89.7 ± 8.4, and 94.3 ± 10.8 and ADS values of 0.686 ± 0.075, 0.751 ± 0.055, and 0.758 ± 0.074.

To check if the propensity for intrinsic disorder is an evolutionary conserved feature of the members of the synuclein family, we analyzed disorder propensity in a variety of evolutionary distinct species, such as *Macaca fascicularis*, *Mus musculus*, *Monodelphis domestica*, *Tachyglossus aculeatus*, *Gallus gallus*, *Pelodiscus sinensis*, *Xenopus laevis*, and *Erpetoichthys calabaricus*. In other words, our analysis encompassed mammals, including a marsupial and an egg-laying monotreme, a bird, a reptile, an amphibian, and a fish. Amino acid sequences of α- (where available), β-, and γ-synucleins from these species were used for the multiple sequence alignments and per-residue disorder analysis. We did not find sequences of α-synucleins from *Monodelphis domestica* and *Tachyglossus aculeatus*, and therefore these proteins were not included in subsequent analyses. The amino acid sequences of all proteins used in these analyses are shown in Supplementary Table S1.

**Figure 10:**
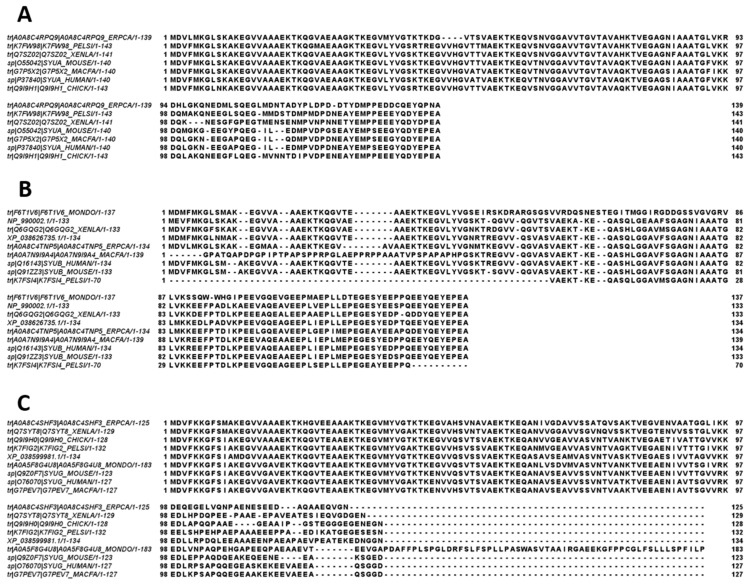
Multiple sequence alignments of α- (**A**), β- (**B**), and γ-synucleins (**C**) conducted by Clustal Omega using default parameters.

Figure 10 represents the results of multiple sequence alignments of these proteins conducted using Clustal Omega [177] and shows remarkable sequence similarity among these intrinsically disordered proteins. In fact, the percent of sequence identity of human protein with the α-synucleins from other species ranged from 76.3% (*Erpetoichthys calabaricus*) to 98.57% (*Macaca fascicularis*) (see Supplementary Figure S1). In the case of human β-synuclein, the percent of sequence identity ranged from 61.07% (*Monodelphis domestica*) to 97.74% (*Mus musculus*) (see Supplementary Figure S2). Finally, human γ-synuclein was shown to have the highest (96.06%) and lowest percent of sequence identity (61.34%) with *Macaca fascicularis* and *Erpetoichthys calabaricus*, respectively (see Supplementary Figure S3).

**Figure 11:**
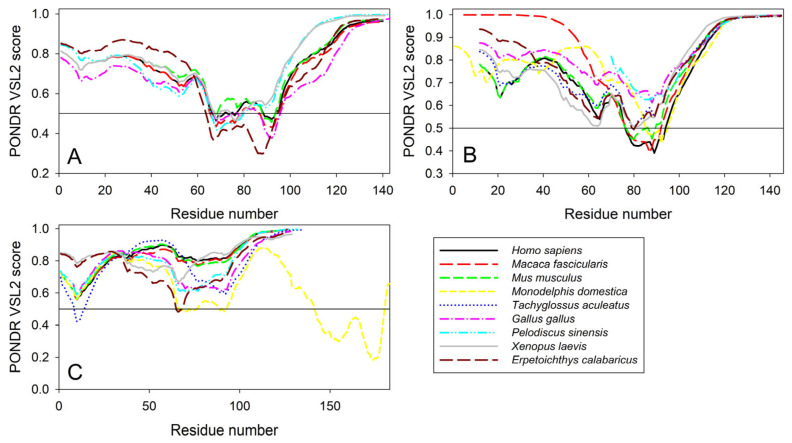
Conservation of the peculiarities of the per-residue intrinsic disorder propensity among the members of synuclein family. Disorder profiles were generated for α- (**A**), β- (**B**), and γ-synucleins (**C**) by PONDR^®^ VSL2.

Furthermore, the global multiple sequence alignment of all 25 synuclein proteins selected for the analysis revealed that these proteins as a group have a sequence similarity that ranges from 30.00% to 98.57% (see Supplementary Figure S4). Based on these observations, it was not surprising to find that the members of the synuclein family are characterized by rather strong conservation of their within-group per-residue disorder profiles (see Figure 11). This analysis indicated that proteins with high levels of intrinsic disorder can be characterized by remarkable evolutionary conservation.

Based on the phylogenetic analysis of the 252 unique synuclein sequences from 73 organisms, it was concluded that γ-synuclein can be considered a common ancestor of the α- and β-synucleins [178]. Furthermore, in line with the results of our analyses, all three synuclein subfamilies were found to be highly conserved [178]. However, it should be emphasized here that the detailed analysis of the evolution of the synuclein family and comprehensive examination of the evolutionary peculiarities of the intrinsic disorder distribution in these proteins are outside the scope of this article, being an exciting and interesting subject of the dedicated study.

It was emphasized that although the analysis of the synucleins of non-mammalian origin would be useful for a better understanding of the evolution and physiological roles of these proteins, currently reported research on synucleins in non-mammalian vertebrates constitutes a very small percentage of the overall publications on this topic [179]. In fact, there are only a very few studies that provide information on the synucleins of amphibians [179,180,181,182,183], birds [184,185,186], fish [187,188,189,190,191], and reptiles [192].

For example, a comprehensive analysis of the spatial and temporal expression patterns of three synucleins during the early embryonic development of *Xenopus laevis* revealed that genes encoding these proteins are most intensely expressed in the nervous system [182]. Based on the facts that at the tadpole stages, synucleins showed distinct expression patterns, with *snca* and *sncbb* being expressed in the brain and retina, *sncbb* showing high expression in the spinal cord, and *sncg* being mainly expressed in the peripheral nervous system, it was concluded that during embryonic development, these proteins have different functions [182]. Since the observed expression patterns of synuclein genes in *Xenopus laevis* were similar to the expression patterns of synucleins in zebrafish [193], *Takifugu rupribes* [188], and chickens [184], it was also indicated that synucleins may have a conserved function in nervous system development [182]. It was also shown that the expression levels of the three synucleins in the green lizard’s *Anolis carolinensis* nervous system were similar to those of human synucleins, confirming the evolutionarily conserved functions of these proteins [192].

In line with the results of our bioinformatics analysis, the recently published study revealed that α-, β-, and γ-synucleins from *Xenopus laevis* are intrinsically disordered in aqueous media but can undergo disorder-to-order transition into an α-helical structure in the presence of the anionic detergent SDS [179].

### 2.4. Functional Disorder Analysis of Human Proteins Engaged in Interaction with Members of Synuclein Family

**Figure 12:**
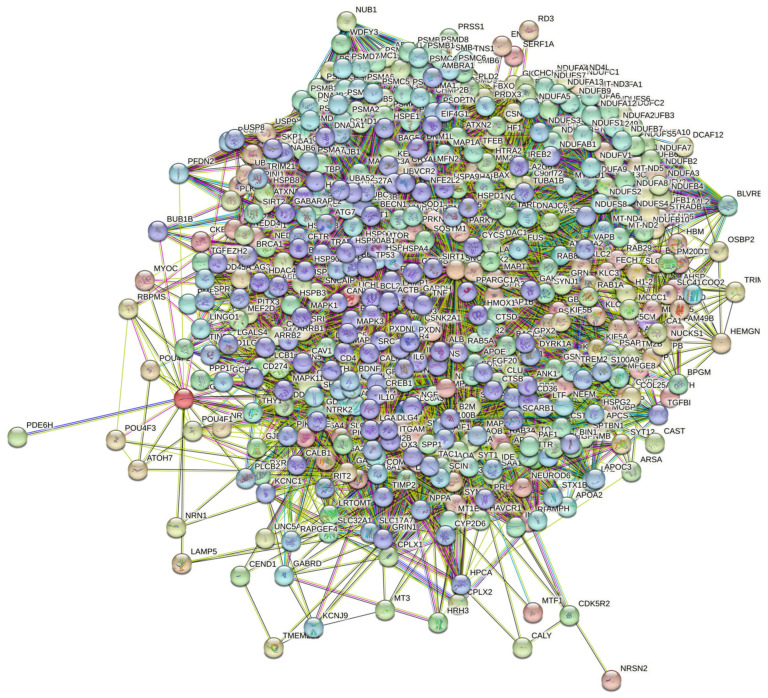
STRING-generated PPI network centered at human α-, β-, and γ-synucleins.

At the next stage, we checked the prevalence of intrinsic disorders in human proteins involved in interactions with α-, β-, and γ-synucleins. PPI networks generated for individual proteins are shown in Figure 5, whereas a global PPI network centered on all three synucleins is shown in Figure 12. This network was generated using a confidence level of 0.45 as the minimum required interaction score. The network includes 469 proteins involved in 10,731 interactions, which significantly exceed the 4889 interactions expected to happen in a random set of proteins of the same size and degree distribution drawn from the genome. The average node degree of this network is 45.8, whereas its average local clustering coefficient is 0.585. The five most enriched biological processes, molecular functions, and cellular components (as per Gene Ontology annotations) of the members of this network, as well as the most enriched local STRING network clusters and KEGG pathways, are listed in Table 1.

**Figure 13:**
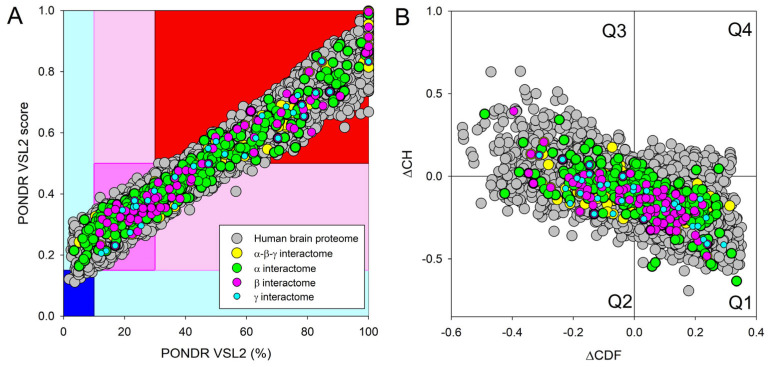
Evaluation of the global disorder status of 10,611 proteins from the human brain proteome (gray circles), as well as the interactomes of individual human synucleins and the global interactome centered at the three synucleins, with corresponding data shown by differently colored circles. (**A**) PONDR^®^ VSL2 score vs. PONDR^®^ VSL2 (%) plot. Here, each point corresponds to a query protein coordinate, which is evaluated from the corresponding PONDR^®^ VSL2 data as its average disorder score (ADS) and percent of the predicted intrinsically disordered residues (PPIDR). Color blocks are used to visualize proteins based on the accepted classification, with red, pink/light pink, and blue/light blue regions containing highly disordered, moderately disordered, and ordered proteins, respectively (see the text). Dark blue or pink regions correspond to the regions where PPIDR agrees with ADS, whereas areas in which only one of these criteria applies are shown by light blue or light pink. (**B**) CH-CDF plot, where the coordinates for a query protein are calculated as the average distance of its CDF curve from the CDF boundary (X axis) and its distance from the CH boundary. Protein classification is based on the quadrant where it is located: Q1, protein predicted to be ordered by both predictors. Q2, protein predicted to be ordered by CH-plot and disordered by CDF. Q3, protein predicted to be disordered by both predictors. Q4, protein predicted to be disordered by CH-plot and ordered by CDF.

Next, we compared the levels of intrinsic disorder in all these interactomes with the disorder status of all proteins in the human brain. The results of this analysis are shown in Figure 13, which clearly indicates that all analyzed protein sets contain noticeable levels of intrinsic disorder. Figure 13A summarizes the results of this analysis in the form of the PONDR^®^ VSL2 score vs. PONDR^®^ VSL2 (%) plot. Based on the results of these analyses, proteins can be classified using the percent of predicted intrinsically disordered residues (PPIDR), i.e., the percent of residues with a disorder score of 0.5 or higher. Here, a PPIDR value of less than 10% is taken to correspond to a highly ordered protein; PPIDR between 10% and 30% is ascribed to a moderately disordered protein; and PPIDR greater than 30% corresponds to a highly disordered protein [175,176]. In addition to PPIDR, the average disorder score (ADS) was calculated for each query protein as a protein length-normalized sum of all the per-residue disorder scores. The resulting ADS values can be used for protein classification as highly ordered (ADS < 0.15), moderately disordered or flexible (ADS between 0.15 and 0.5), and highly disordered (ADS ≥ 0.5). Figure 13B represents the results of global disorder analysis in the form of the ΔCH-ΔCDF plot that can be used for further classification of proteins as mostly ordered, molten globule-like or hybrid, or highly disordered based on their positions within the resulting CH-CSD phase space [109,194,195,196]. The results of the corresponding classification are summarized in Table 2. This analysis revealed that although proteins in the joint α-β-γ synuclein interactome and especially proteins interacting with human α-synuclein are somewhat less disordered than proteins in the human brain proteome, interactors of β- and especially γ-synuclein are noticeably more disordered. In fact, as per PONDR^®^ VSL2 analysis, all proteins interacting with β- and γ-synucleins are moderately or highly disordered.

Table 2 provides further illustration for this observation and also shows that, on average, most of the proteins in these various sets are classified as moderately or highly disordered, emphasizing the potential importance of intrinsic disorder for the functionality of these proteins.

**Figure 14:**
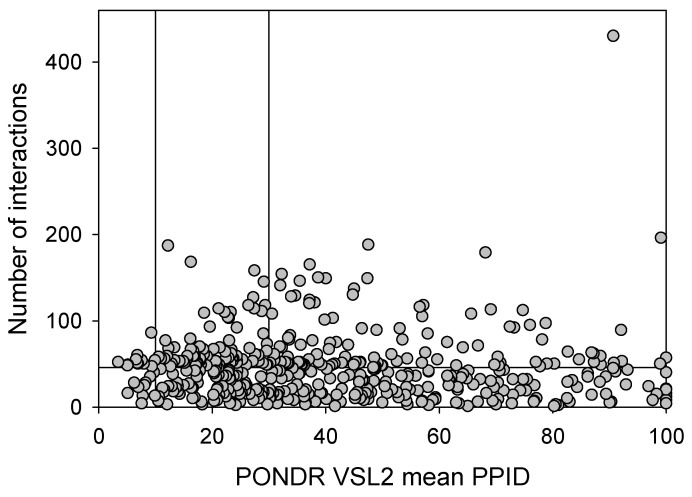
Correlation between the number of interactions and intrinsic disorder level of human proteins in the joint α-β-γ synuclein interactome. A vertical solid line represents the average node degree of this network (which is 45.8). Two verical solid lines represent two disorder boundaries of 15% and 30%.

Next, we took a look at the intractability of different proteins from the joint α-β-γ synuclein interactome and compared the corresponding node degree of these proteins with their disorder status. The results of this analysis are shown in Figure 14. In this network, almost half of the proteins (207 of 467, 44.3%) are involved in more than 47 interactors each, indicating that these proteins can be considered hubs. These hub proteins are characterized by a mean node degree of 76 ± 41 and a mean PPID of 37.8 ± 22.8%. Our analysis revealed that 60 proteins with the least number of interactors (with 10 or fewer partners each) were characterized by a mean node degree of 6.0 ± 2.7 and a mean PPID of 51.4 ± 25.9%. On the other hand, the 60 most connected proteins were characterized by a mean node degree of 123 ± 43 and a mean PPID of 43.1 ± 21.4%. Curiously, the 60 most disordered proteins in this dataset had a mean node degree of 44.2 ± 60.6 and a mean PPID of 87.5 ± 8.4%, whereas the 60 most ordered proteins in this set were characterized by a mean node degree of 43.7 ± 32.0 and a mean PPID of 11.1 ± 11.6%. These data taken together indicated that generally, proteins with lower disorder levels are expected to engage in a bit more interactions. However, the situation changes if one compares the 20 most ordered proteins (PPID of 7.5 ± 1.8%) with the 20 most disordered proteins (PPID of 96.6 ± 3.9%), as their interactomes range from 4 to 430 and from 4 to 86 proteins, respectively.

**Figure 15:**
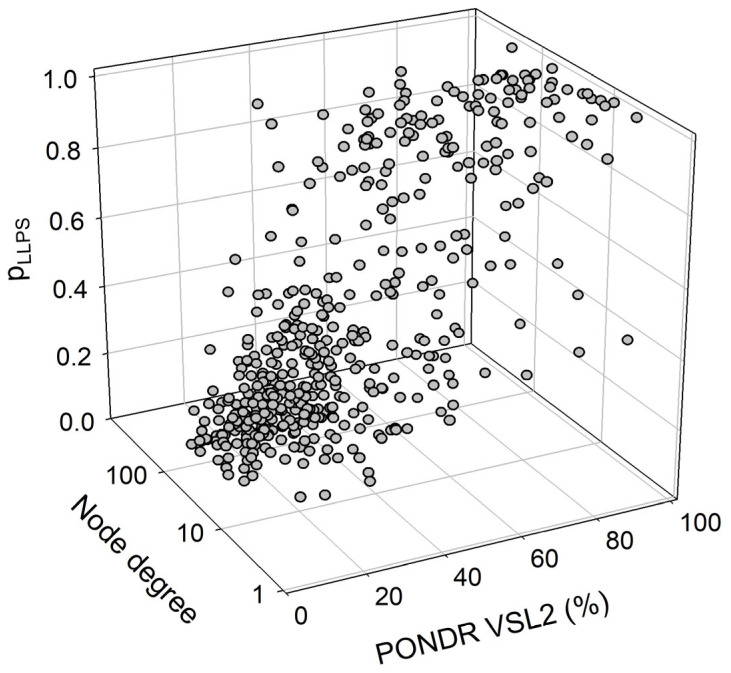
A correlation between the overall disorder status (PONDR^®^ VSL2, %), interactability (node degree), and LLPS predisposition (p_LLPS_) of 467 human proteins in the joint α-β-γ synuclein interactome.

We also looked for a correlation between the overall disorder status, intractability, and LLPS predisposition of human proteins in the joint α-β-γ synuclein interactome. The results of this analysis are summarized in Figure 15, which shows the corresponding outputs in the form of a 3D plot. This analysis revealed that proteins predicted by FuzDrop as droplet drivers (i.e., possessing p_LLPS_ ≥ 0.6) are on average more disordered than proteins that are not capable of spontaneous liquid–liquid phase separation. In fact, 130 proteins with p_LLPS_ ≥ 0.6 were characterized by a mean PPIDR of 66.3 ± 19.5%, whereas the remaining 337 proteins from the joint human α-β-γ synuclein interactome were characterized by a mean PPIDR of 31.4 ± 18.4%. On the other hand, LLPS drivers and non-drivers did not show a noticeable difference in their within network interactivity: within the joint human α-β-γ synuclein interactome, their corresponding mean node degrees were 40.4 ± 49.3% (drivers) and 48.0 ± 35.6% (non-drivers), respectively. Comparative analysis of the 130 most disordered proteins revealed that they are characterized by a mean PPIDR of 74.5 ± 14.0%, a mean node degree of 41.7 ± 48.2, and a mean p_LLPS_ of 0.753 ± 0.295. The remaining 337 proteins are characterized by a mean PPIDR of 28.3 ± 12.4%, a mean node degree of 47.4 ± 35.2, and a mean p_LLPS_ of 0.311 ± 0.229. Comparative analysis of the 130 most connected proteins with a mean node degree of 90.8 ± 45.6 revealed that they are characterized by a mean PPIDR of 38.4 ± 22.2% and a mean p_LLPS_ of 0.379 ± 0.273. The remaining less interactive human proteins in the joint α-β-γ synuclein interactome have a mean node degree of 28.6 ± 16.3, a mean PPIDR of 42.2 ± 25.2%, and a mean p_LLPS_ of 0.456 ± 0.331.

A detailed description of the prevalence and functionality of intrinsic disorder in the sets of 11 most disordered and 5 most ordered members of the joint α-β-γ synuclein interactome is presented in Appendix A and Appendix B, respectively.

## 3. Materials and Methods

### 3.1. Overview

In order to facilitate sequence-based and structure-based comparison of synuclein proteins of different species, we utilized web-based computational tools such as UniProt [197], NCBI Blast, and AlphaFold [198]. To perform disorder-based analysis and comparison, we utilized the RIDAO application [199], a computational tool, to identify the predicted disorder throughout the amino acid sequence. Further, we also utilized the D^2^P^2^ tool [200] and the FuzDrop tool [147,148,201] to examine intrinsic disorder and predict liquid–liquid phase separation (LLPS). We conducted extensive analysis of interacting proteins through the STRING database [202] to enable disorder-based comparison of the human synuclein family with the proteins in their respective interactomes.

### 3.2. Sequence and Structure-Based Analysis

We utilized the UniProt database [197] to extract the amino acid sequence information for human α-synuclein, β-synuclein, and γ-synuclein. UniProt is a database that provides the known amino acid sequences along with additional information regarding species and protein identity. Utilizing the extracted amino acid sequence, we visualized the predicted 3D structure of the proteins using the AlphaFold platform [198]. AlphaFold is an AI-based computational tool that predicts the 3D structure of a protein given its amino acid sequence. Having analyzed the sequence and structure of the synuclein family of proteins, we utilized the NCBI Blast tool to compare the human synuclein family sequences with those of other species. To this end, we used UniProt to find the amino acid sequences of α-, β-, and γ-synucleins from different species using “(protein_name:”alpha-synuclein”) AND (gene:SNCA) NOT fragment”, “(protein_name:”beta-synuclein”) AND (gene:SNCB) NOT fragment”, and “(protein_name:”gamma-synuclein”) AND (gene:SNCG) NOT fragment” as search criteria. This search resulted in 381 α- synucleins, 320 β- synucleins, and 234 γ-synucleins, which were used for global disorder analysis. Next, we selected eight species from different classes of animals, such as *Macaca fascicularis*, *Mus musculus*, *Monodelphis domestica*, *Tachyglossus aculeatus*, *Gallus gallus*, *Pelodiscus sinensis*, *Xenopus laevis*, and *Erpetoichthys calabaricus*. We extracted the amino acid sequences of the three synuclein proteins for each of these species using UniProt and performed a sequence-based comparison with the corresponding human synucleins using NCBI Blast. Further, we analyzed the intrinsic disorder of the synucleins of these species using the RIDAO platform.

### 3.3. Disorder-Based Analysis of the Interactomes of Human Synucleins

Having performed sequence- and structure-based comparisons of human synucleins with the synucleins of various species, we performed detailed intrinsic disorder analysis of the three human synucleins and the proteins in their interactomes. To this end, we utilized the STRING database [202] to identify proteins that are known to interact with human α-, β-, and γ-synucleins. The STRING database assembles information from different sources, such as laboratory experiments, previous research, and text mining models. The STRING database takes the query protein sequence as input and provides a network of interacting proteins with varying levels of confidence. The interacting proteins are sorted by confidence, with a score of 0.7 or above being termed high confidence, a score of 0.4 being considered medium confidence, and a score of 0.15 or below being taken as low confidence. Additional customization allows us to specify the maximum number of interactors in the first shell (the proteins directly interacting with the target protein). Known 3D structures of interactors and the nature of interactions (known interaction or predicted interaction) are also provided. We specify a maximum of 500 interactors in the first shell to enable an extensive search of the interacting proteins.

Further, we predict intrinsic disorder for each of these proteins using the RIDAO platform [199] and, based on the outputs of CH-CDF analysis incorporated into RIDAO, label them as ‘disordered’, ‘mixed’, ‘rare’, or ‘structured’. We selected the first 10 most disordered proteins in the interactomes of each member of the synuclein family and performed a detailed intrinsic disorder analysis with the RIDAO and the D^2^P^2^ platforms [200]. Further, we analyze the propensity of these proteins for liquid–liquid phase separation (LLPS) with the FuzDrop computational platform [147,148,201].

## 4. Conclusions

This work provides a discussion of the sequence-based and structure-based functionality of the proteins of the human synuclein family. Through comparative inter-species sequence-based analysis, various insights regarding the similarity of α-, β-, and γ-synucleins from different species are obtained. Intrinsic disorder analysis demonstrates the presence of disordered, ordered, and mixed members in the human joint α-β-γ interactome. Interestingly, comprehensive disorder analysis reveals the presence of a significant percentage of intrinsically disordered interacting proteins in the interactomes of human α-, β-, and γ-synucleins. The analysis of the liquid–liquid phase separation probability of human synucleins and their interactors provides important insights into the potential roles of intrinsic disorder in the organization of synuclein-related MLOme. Finally, we explore the potential functionality of intrinsic disorder in a set of the most disordered members of the joint α-β-γ interactome using a set of bioinformatics tools.

## Figures and Tables

**Table 1:** Functional enrichment of the networks centered at human α-, β-, and γ-synucleins.

Protein	ID	Description	Order of Magnitude of the *p*-Value
**α-synuclein**	**Biological Process (Gene Ontology)**		
	GO:0006120	Mitochondrial electron transport, NADH to ubiquinone	−44
	GO:0007005	Mitochondrion organization	−44
	GO:0042776	Proton motive force-driven mitochondrial ATP synthesis	−43
	GO:0006810	Transport	−41
	GO:0051179	Localization	−41
	**Molecular Function (Gene Ontology)**		
	GO:0008137	NADH dehydrogenase (ubiquinone) activity	−46
	GO:0019899	Enzyme binding	−36
	GO:0009055	Electron transfer activity	−35
	GO:0015399	Primary active transmembrane transporter activity	−31
	GO:0005515	Protein binding	−29
	**Cellular Component (Gene Ontology)**		
	GO:0005747	Mitochondrial respiratory chain complex I	−49
	GO:0005737	Cytoplasm	−47
	GO:0098803	Respiratory chain complex	−42
	GO:0070469	Respirasome	−42
	GO:0005746	Mitochondrial respirasome	−42
	**Local Network Cluster (STRING)**		
	CL:11079	NADH dehydrogenase (ubiquinone) activity	−46
	CL:11077	Respiratory chain complex	−42
	CL:11070	Respiratory chain complex, and Complex I biogenesis	−41
	CL:11080	NADH dehydrogenase (ubiquinone) activity	−39
	CL:11066	Respiratory electron transport, ATP synthesis by chemiosmotic coupling, and heat production by uncoupling proteins, and respiratory chain complex IV	−37
	**KEGG Pathways**		
	hsa05012	Parkinson’s disease	−124
	hsa05014	Amyotrophic lateral sclerosis	−99
	hsa05010	Alzheimer’s disease	−97
	hsa05020	Prion disease	−95
	hsa05016	Huntington’s disease	−87
**β-synuclein**	**Biological Process (Gene Ontology)**		
	GO:0099003	Vesicle-mediated transport in synapse	−23
	GO:0001505	Regulation of neurotransmitter levels	−22
	GO:0007268	Chemical synaptic transmission	−22
	GO:0099504	Synaptic vesicle cycle	−21
	GO:0006836	Neurotransmitter transport	−21
	**Molecular Function (Gene Ontology)**		
	GO:1903136	Cuprous ion binding	−6
	GO:0000149	SNARE binding	−6
	GO:0005507	Copper ion binding	−5
	GO:0019899	Enzyme binding	−5
	GO:0015318	Inorganic molecular entity transmembrane transporter activity	−4
	**Cellular Component (Gene Ontology)**		
	GO:0043005	Neuron projection	−38
	GO:0098793	Presynapse	−37
	GO:0045202	Synapse	−35
	GO:0030424	Axon	−33
	GO:0036477	Somatodendritic compartment	−31
	**Local Network Cluster (STRING)**		
	CL:14440	Mixed, including early-onset Parkinson’s disease, and C-terminal of Roc (COR) domain	−13
	CL:23285	Neurotransmitter transport, and RIMS-binding protein, third SH3 domain	−12
	CL:23286	Mixed, including synaptic vesicle pathway, and Cytoskeleton of presynaptic active zone	−10
	CL:14443	Early-onset Parkinson’s disease	−9
	CL:23287	Mixed, including presynaptic active zone cytoplasmic component, and Clathrin-sculpted vesicle	−9
	**KEGG Pathways**		
	hsa04721	Synaptic vesicle cycle	−8
	hsa05012	Parkinson’s disease	−6
	hsa05033	Nicotine addiction	−5
	hsa04726	Serotonergic synapse	−2
	hsa04911	Insulin secretion	−2
**γ-synuclein**	**Biological Process (Gene Ontology)**		
	GO:0006836	Neurotransmitter transport	−5
	GO:0001505	Regulation of neurotransmitter levels	−5
	GO:0050885	Neuromuscular process controlling balance	−4
	GO:0099504	Synaptic vesicle cycle	−4
	GO:0007399	Nervous system development	−4
	**Molecular Function (Gene Ontology)**		
	GO:0005326	Neurotransmitter transmembrane transporter activity	−4
	GO:1903136	Cuprous ion binding	−3
	GO:0017075	Syntaxin-1 binding	−2
	GO:0015370	Solute:sodium symporter activity	−2
	GO:0015108	Chloride transmembrane transporter activity	−2
	**Cellular Component (Gene Ontology)**		
	GO:0043005	Neuron projection	−12
	GO:0120025	Plasma membrane bounded cell projection	−11
	GO:0030424	Axon	−10
	GO:0150034	Distal axon	−9
	GO:0098793	Presynapse	−9
	**Local Network Cluster (STRING)**		
	CL:23829	Mixed, including antibiotic biosynthesis monooxygenase, and Synuclein	−3
	CL:20559	Mixed, including habenula development, and Regulation of retinal ganglion cell axon guidance	−3
	CL:23287	Mixed, including presynaptic active zone cytoplasmic component, and clathrin-sculpted vesicle	−2
	CL:23831	Mixed, including synuclein, and negative regulation of myoblast fusion	−2
	CL:23313	Mixed, including autosomal dominant nonsyndromic deafness 25, and ureter cancer	−2
	**KEGG Pathways**		
	hsa04721	Synaptic vesicle cycle	−5
	hsa04723	Retrograde endocannabinoid signaling	−4
	hsa04726	Serotonergic synapse	−3
	hsa04724	Glutamatergic synapse	−3
	hsa04912	GnRH signaling pathway	−2
**α-synuclein + β-synuclein + γ-synuclein**	**Biological Process (Gene Ontology)**		
	GO:0051179	Localization	−44
	GO:0006810	Transport	−42
	GO:0051234	Establishment of localization	−42
	GO:0007005	Mitochondrion organization	−40
	GO:0006120	Mitochondrial electron transport, NADH to ubiquinone	−40
	**Molecular Function (Gene Ontology)**		
	GO:0008137	NADH dehydrogenase (ubiquinone) activity	−41
	GO:0019899	Enzyme binding	−38
	GO:0005515	Protein binding	−34
	GO:0009055	Electron transfer activity	−31
	GO:0015399	Primary active transmembrane transporter activity	−26
	**Cellular Component (Gene Ontology)**		
	GO:0005737	Cytoplasm	−51
	GO:0031982	Vesicle	−45
	GO:0005747	Mitochondrial respiratory chain complex I	−44
	GO:0043005	Neuron projection	−42
	GO:0031410	Cytoplasmic vesicle	−41
	**Local Network Cluster (STRING)**		
	CL:11079	NADH dehydrogenase (ubiquinone) activity	−41
	CL:11077	Respiratory chain complex	−37
	CL:11070	Respiratory chain complex, and Complex I biogenesis	−35
	CL:11080	NADH dehydrogenase (ubiquinone) activity	−35
	CL:11066	Respiratory electron transport, ATP synthesis by chemiosmotic coupling, and heat production by uncoupling proteins, and respiratory chain complex IV	−32
	**KEGG Pathways**		
	hsa05012	Parkinson disease	−111
	hsa05014	Amyotrophic lateral sclerosis	−94
	hsa05010	Alzheimer disease	−88
	hsa05020	Prion disease	−87
	hsa05016	Huntington disease	−82

**Table 2:** Distribution of human synuclein-interacting proteins among different disorder categories.

Dataset	Protein Number	PONDR^®^ VSL2 Score vs. PONDR^®^ VSL2 (%) Plot					CH-CDF Plot			
		Blue	Cyan	Dark Pink	Pink	Red	Q1	Q2	Q3	Q4
Human brain proteome	10,611	15 (0.15%)	411 (3.87%)	3593 (33.86%)	2335 (22.00%)	4257 (40.12%)	6203 (58.5%)	2938 (27.7%)	1193 (11.2%)	277 (2.6%)
Joint α-β-γ interactome	467	0 (0.0%)	22 (4.7%)	172 (36.8%)	110 (23.6%)	163 (34.9%)	292 (62.5%)	105 (22.5%)	61 (13.1%)	9 (1.9%)
α-Synuclein interactome	356	0 (0.0%)	20 (5.6%)	135 (37.9%)	89 (25.0%)	112 (31.5%)	234 (65.7%)	65 (18.3%)	48 (13.5%)	9 (2.5%)
β-Synuclein interactome	85	0 (0.0%)	0 (0.0%)	30 (35.3%)	19 (22.3%)	36 (32.4%)	48 (56.5%)	26 (30.6%)	11 (12.9%)	0 (0.0%)
γ-Synuclein interactome	32	0 (0.0%)	0 (0.0%)	12 (37.5%)	4 (12.5%)	16 (50.0%)	14 (43.75%)	14 (43.75%)	4 (12.5%)	0 (0.0%)

**Figure A1:**
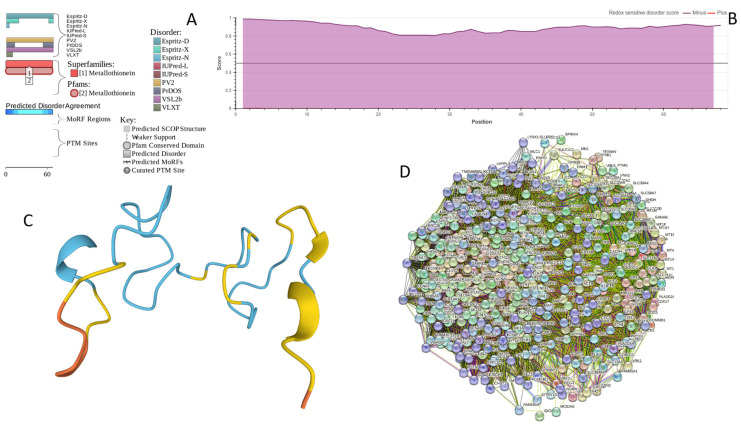
Functional disorder analysis of human Metallothionein-3 (UniProt ID: P25713). (**A**) Functional disorder profile generated by D^2^P^2^. (**B**) Redox-sensitive disorder profile generated by IUPred2A. (**C**) 3D structural model generated by AlphaFold. (**D**) CHMP2B-centered PPI network generated by STRING. This network was generated using medium confidence level of 0.185.

**Figure A2:**
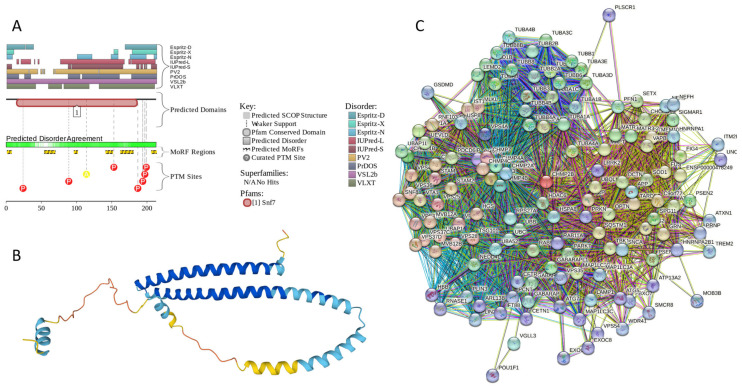
Functional disorder analysis of human CHMP2B (Q9UQN3). (**A**) Functional disorder profile generated by D^2^P^2^. (**B**) 3D structural model generated by AlphaFold. (**C**) CHMP2B-centered PPI network generated by STRING. This network was generated using medium confidence level of 0.4.

**Figure A3:**
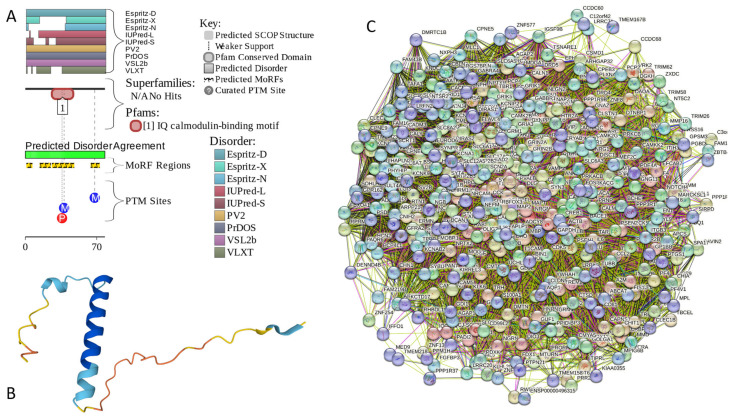
Functional disorder analysis of human neurogranin (UniProt ID: Q92686). (**A**) Functional disorder profile generated by D^2^P^2^. (**B**) 3D structural model generated by AlphaFold. (**C**) NRGN-centered PPI network generated by STRING. This network was generated using low confidence of 0.15 for minimum required interaction score.

**Figure A4:**
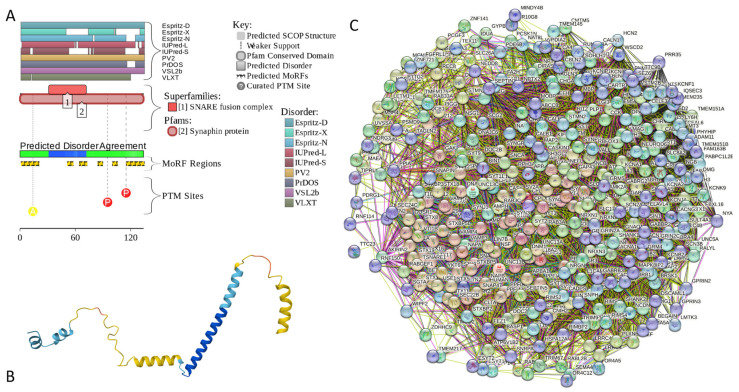
Functional disorder analysis of human complexin-1 (UniProt ID: O14810). (**A**) Functional disorder profile generated by D^2^P^2^. (**B**) 3D structural model generated by AlphaFold. (**C**) CPLX1-centered PPI network generated by STRING. This network was generated using a custom confidence value of 0.225 for minimum required interaction score.

**Figure A5:**
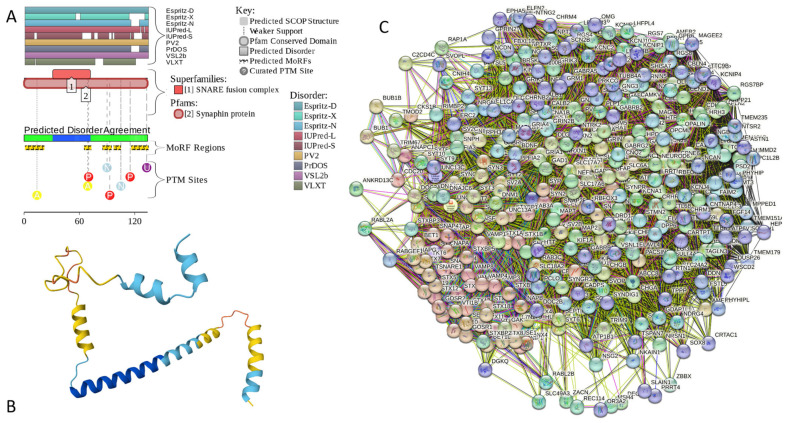
Functional disorder analysis of human complexin-2 (UniProt ID: Q6PUV4). (**A**) Functional disorder profile generated by D^2^P^2^. (**B**) 3D structural model generated by AlphaFold. (**C**) CPLX2-centered PPI network generated by STRING. This network was generated using a custom confidence value of 0.225 for minimum required interaction score.

**Figure A6:**
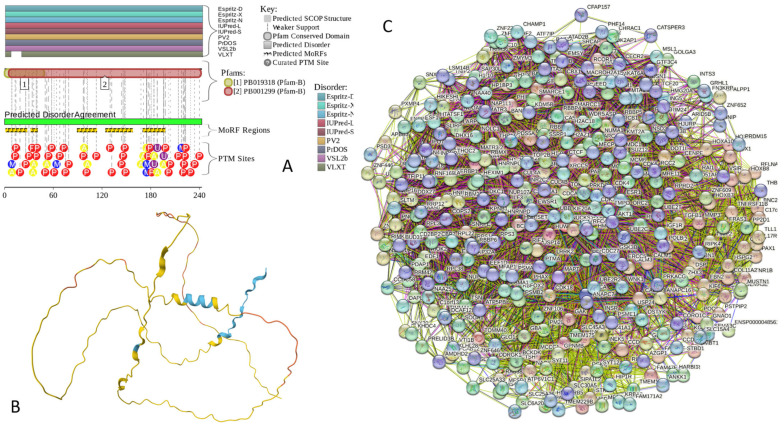
Functional disorder analysis of human NUCKS1 (UniProt ID: Q9H1E3). (**A**) Functional disorder profile generated by D^2^P^2^. (**B**) 3D structural model generated by AlphaFold. (**C**) NUCKS1-centered PPI network generated by STRING. This network was generated using a custom confidence value of 0.200 for minimum required interaction score.

**Figure A7:**
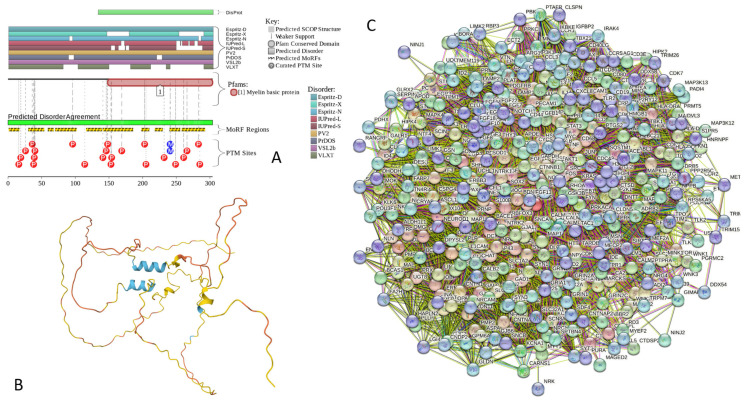
Functional disorder analysis of human MBP (UniProt ID: P02686). (**A**) Functional disorder profile generated by D^2^P^2^. (**B**) 3D structural model generated by AlphaFold. (**C**) MBP-centered PPI network generated by STRING. This network was generated using a custom confidence value of 0.300 for minimum required interaction score.

**Figure A8:**
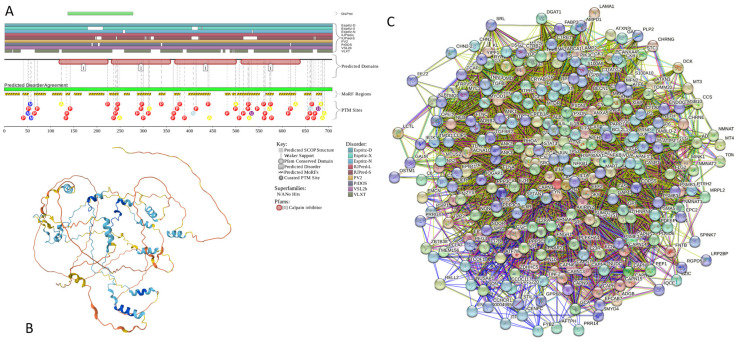
Functional disorder analysis of human Calpastatin (UniProt ID: P20810). (**A**) Functional disorder profile generated by D^2^P^2^. (**B**) 3D structural model generated by AlphaFold. (**C**) CAST-centered PPI network generated by STRING. This network was generated using a custom confidence value of 0.200 for minimum required interaction score.

**Figure A9:**
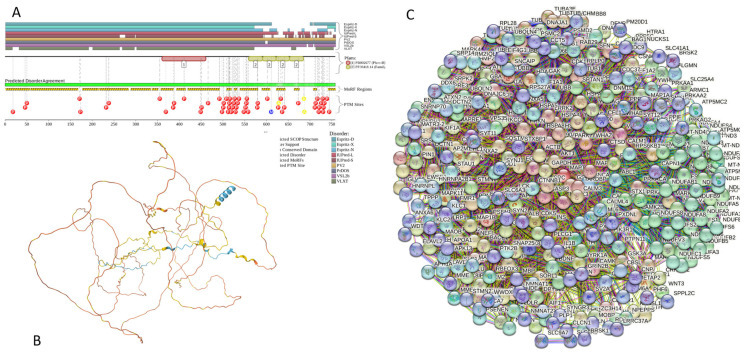
Functional disorder analysis of human MAPT (UniProt ID: P10636). (**A**) Functional disorder profile generated by D^2^P^2^. (**B**) 3D structural model generated by AlphaFold. (**C**) MAPT-centered PPI network generated by STRING. This network was generated using a custom confidence value of 0.500 for minimum required interaction score.

**Figure A10:**
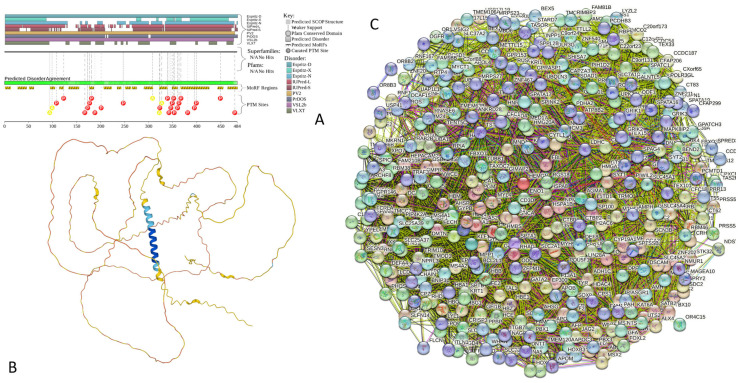
Functional disorder analysis of human hemogen (UniProt ID: Q9BXL5). (**A**) Functional disorder profile generated by D^2^P^2^. (**B**) 3D structural model generated by AlphaFold. (**C**) HEMGN-centered PPI network generated by STRING. This network was generated using a low confidence of 0.150 for minimum required interaction score.

**Figure A11:**
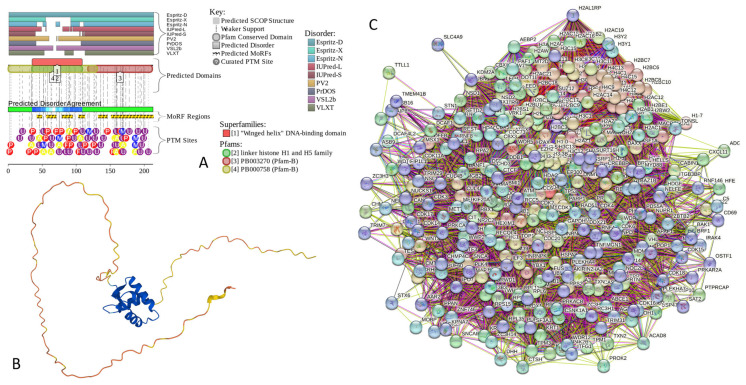
Functional disorder analysis of human Histone H1.2 (UniProt ID: P16403). (**A**) Functional disorder profile generated by D^2^P^2^. (**B**) 3D structural model generated by AlphaFold. (**C**) H1.2-centered PPI network generated by STRING. This network was generated using a custom confidence value of 0.250 for minimum required interaction score.

**Figure A12:**
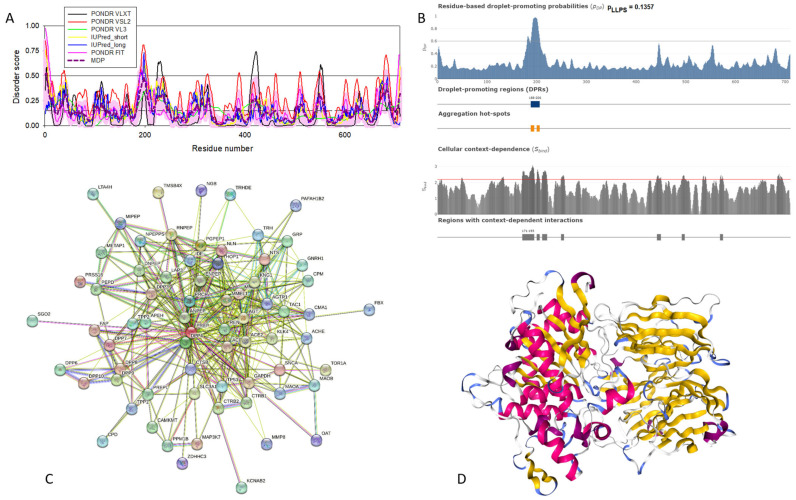
Structural and functional characterization of human prolyl endopeptidase (UniProt ID: P48147). (**A**) Intrinsic disorder profile generated by RIDAO. (**B**) FuzDrop output. (**C**) PREP-centered PPI network generated by STRING. This network was generated using the custom confidence value of 0.400 for the minimum required interaction score. (**D**) X-ray crystal structure of human prolyl endopeptidase (PDB ID: 3DDU).

**Figure A13:**
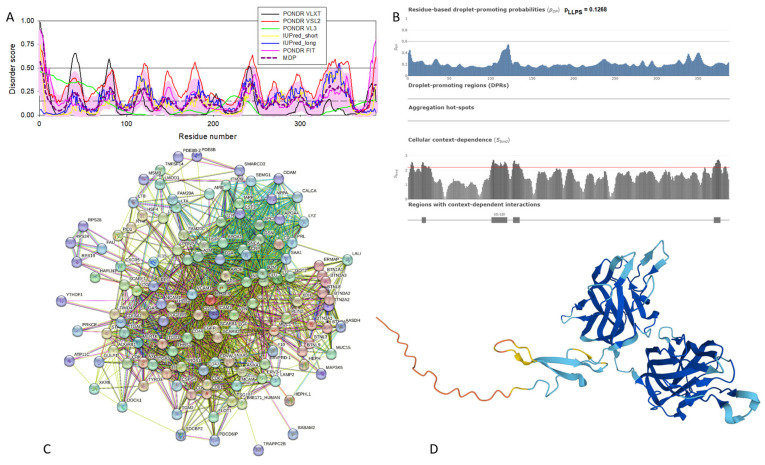
Structural and functional characterization of human lactadherin (UniProt ID: Q08431). (**A**) Intrinsic disorder profile generated by RIDAO. (**B**) FuzDrop output. (**C**) Lactadherin-centered PPI network generated by STRING. This network was generated using the custom confidence value of 0.400 for the minimum required interaction score. (**D**) A 3D structural model generated by AlphaFold.

**Figure A14:**
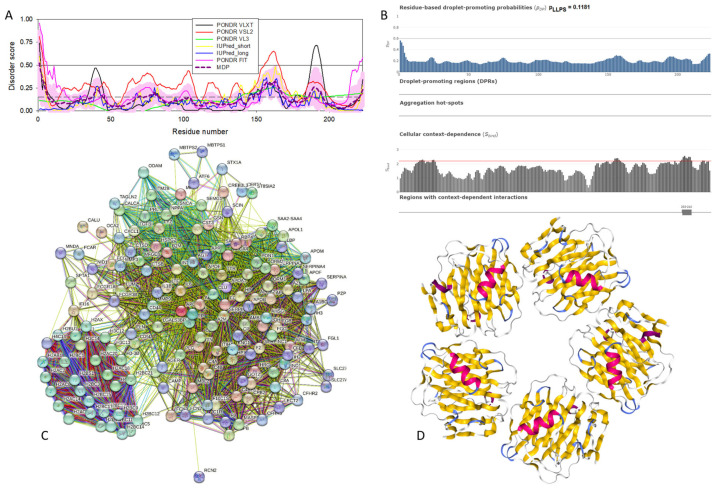
Structural and functional characterization of human SAP (UniProt ID: P02743). (**A**) Intrinsic disorder profile generated by RIDAO. (**B**) FuzDrop output. (**C**) SAP-centered PPI network generated by STRING. This network was generated using the custom confidence value of 0.400 for the minimum required interaction score. (**D**) X-ray crystal structure of the pentameric human serum amyloid P-component (PDB ID: 4AVS).

**Figure A15:**
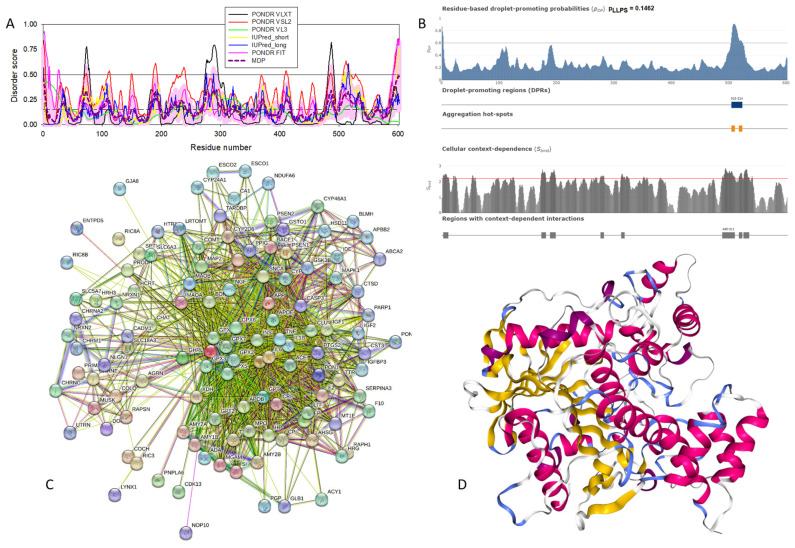
Structural and functional characterization of human cholinesterase (UniProt ID: P06276). (**A**) Intrinsic disorder profile generated by RIDAO. (**B**) FuzDrop output. (**C**) Cholinesterase-centered PPI network generated by STRING. This network was generated using the custom confidence value of 0.400 for the minimum required interaction score. (**D**) X-ray crystal structure of human cholinesterase (PDB ID: 1P0I).

**Figure A16:**
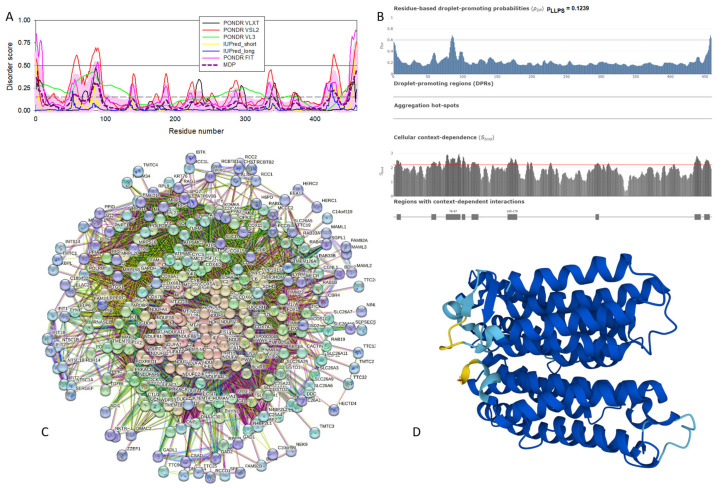
Structural and functional characterization of human NADH-ubiquinone oxidoreductase chain 4 (MT-ND4, UniProt ID: P03905). (**A**) Intrinsic disorder profile generated by RIDAO. (**B**) FuzDrop output. (**C**) MT-ND4-centered PPI network generated by STRING. This network was generated using the custom confidence value of 0.400 for the minimum required interaction score. (**D**) 3D structural model generated by AlphaFold.

## Data Availability

The data are contained within the article and Supplementary Materials.

## Supplementary Materials

The following supporting information can be downloaded at: https://www.mdpi.com/article/10.3390/ijms25158399/s1.

## Appendix A. Functionality of Disorder in 11 Most Disordered Proteins from the Joint α-β-γ Synuclein Interactome

Results of the PONDR^®^ VSL2-based analysis of intrinsic disorder predisposition of the proteins from the joint α-β-γ synuclein interactome revealed that among the 467 members of this set, 144 (i.e., 30.8%) were mostly disordered, being predicted to have PPIDR of at least 50%. Furthermore, 24 of these proteins had a PPIDR exceeding 90%. In other words, these almost entirely disordered proteins accounted for 5.1% of the whole joint α-β-γ synuclein interactome or constituted 16.7% of the mostly disordered set of α-β-γ interactors. Furthermore, nine proteins (MT3, CHMP2B, NRGN, CPLX1, CPLX2, NUCKS1, SNCG, MBP, and CAST) were predicted to be completely disordered (they have a PPIDR of 100%). In agreement with these observations, PONDR^®^ VL3 (a tool specifically designed for finding long disordered regions and fully disordered proteins) confirmed the 100% disorder status of these proteins and predicted four more proteins (MAPT, HEMGN, H1–2, and SNCA) to have a PPIDR of 100%. Since the disorder-centric functionality of SNCA (α-synuclein) and SNCG (γ-synuclein) was already introduced, the sections below provide a brief description of the 11 remaining completely disordered proteins.

### Appendix A.1. MT3 (Metallothionein-3; UniProt ID: P25713; PPIDR_PONDR_^®^ _VSL2_ = 100.0%; ADS _PONDR_^®^ _VSL2_ = 0.9952)

Metallothionein-3 (MT3) is one of the major intracellular zinc-binding proteins that play a number of important regulatory roles in the uptake, distribution, storage, and release of zinc [203]. In mammals, the family of metallothioneins includes four members with specific tissue distributions, where MT1 and MT2 are found in all organs, whereas MT-3 is expressed mainly in the brain and MT-4 is mostly found in the stratified squamous epithelial tissues [203,204,205]. MT3 is also known as human neuronal growth inhibitory factor (hGIF), since it is known to inhibit the outgrowth of embryonic cortical neurons [206]. Based on the analysis of the native MT3 purified from the human brain, it was established that a single molecule of this protein contains seven metal ions: three Zn^2+^ and four Cu^+^ ions, which are bound in the form of homo metal-thiolate clusters to two specific domains: a cooper-binding N-terminal β-domain (residues 1–30) and a zinc-binding C-terminal α-domain (residues 31–68) [207]. The neuron inhibitory activity of the MT3/hGIH is driven by the Cys6-Pro7-Cys8-Pro9 motif located within the β-domain of this protein, with a crucial role being played by its two proline residues, as their substitution entirely abolishes the activity of this domain [208,209,210].

Importantly, it was established that MT3 is deficient in Alzheimer’s disease brain [211], as well as in other neurodegenerative diseases, such as multiple-system atrophy, Parkinson’s disease, progressive supranuclear palsy, and amyotrophic lateral sclerosis [212,213,214], with the reduced levels of this protein in the subset of reactive astrocytes in lesioned areas associated with the aforementioned diseases being correlated with the neuronal loss [215]. Altogether, MT3 was reported as a multifunctional player in the control of cellular processes and diseases [214]. In fact, MT3 is not only responsible for maintaining the homeostasis of copper and zinc in cells and acts as a neuronal growth-inhibitory factor, but it also plays a role in the protection of cells from oxidative stress and regulates a broad spectrum of cellular processes, such as cell growth and differentiation [214,216].

Structural information is available for the metal-bound forms of the α-domain (e.g., [217]), whereas no sufficient long- and medium-range Nuclear Overhauser effect (NOE) signals are available for the NMR-based structural determination of the β-domain of hGIF due to extensive internal dynamics [217,218]. Almost no structural information is available for the highly dynamic apo-MT3, which was shown to exist in a compact conformation (likely resembling a molten globule form) under physiological conditions [219].

MT3 is a 68-residue-long protein with a very unusual amino acid composition: it does not have any arginine, asparagine, histidine, isoleucine, leucine, phenylalanine, tryptophan, or tyrosine residues but includes 20 (29.4%) cysteine residues, 8 (11.8%) of each glutamic acid and lysine residues, as well as 7 (10.3%) of each alanine and serine residues. Because of this high cysteine content, many disorder predictors do not classify MT3 as a disordered protein since cysteines are typically considered the strongest order-promoting residues. However, Figure A1A shows that PONDR^®^ VSL2 identifies this protein as completely disordered. Although both short and long forms of the IUPred classifier showed PPIDR of 0%, the use of the context-dependent mode of the IUPred2A predictor [220] revealed that the entire protein represents a redox-sensitive region that is expected to be completely ordered in the oxidized form and completely disordered in the reduced form (see Figure A1B). Figure A1C represents the AlphaFold-modeled 3D structure of human MT3 and shows that this protein almost does not contain regular secondary structure elements. The MT3-centered PPI network generated by STRING is shown in Figure A1D, which illustrates that this protein forms a densely connected network containing 415 proteins connected by 25,163 interactions (the expected number of edges is 3897). The network is characterized by an average node degree of 121, an average local clustering coefficient of 0.668, and a PPI enrichment *p*-value of <1.0 × 10^−16^. Among the members of this network are α-synuclein (SNCA) and β-synuclein (SNCB), which are involved in interactions with MT3, 156, and 212 other proteins. In line with these observations, MT3 was shown to co-localize with α-synuclein glial cytoplasmic inclusions (GCIs), which are multiple system atrophy-related intracytoplasmic inclusion bodies found in the oligodendrocytes [221]. Finally, human MT3 was predicted to have a low p_LLPS_ of 0.3717, indicating that this protein is not capable of spontaneous LLPS and cannot act as a droplet driver. Since MT3 also does not have DPRs, it also cannot operate as a droplet client.

### Appendix A.2. CHMP2B (Charged Multivesicular Body Protein 2b; UniProt ID: Q9UQN3; PPIDR_PONDR_^®^ _VSL2_ = 100.0%; ADS _PONDR_^®^ _VSL2_ = 0.8144)

As it follows from its name, charged multivesicular body protein 2b (CHMP2B) is involved in the formation of multivesicular bodies (MVBs). This 213 residue-long protein is evolutionary conserved and represents a core component of the Endosomal Sorting Complex Required for Transport III (ESCRT-III) machinery that plays a crucial role in the MVB biogenesis and sorting of the endosomal cargo [222], as well as controlling a number of other fundamental cellular processes, such as autophagy [223], cytokinesis [224,225], endo/lysosomal repair [226], and viral exocytosis [227]. Importantly, mutations in CHMP2B (I29V, T104N, D148Y, Q165X, M178V, and Q206H [228], as well as the *CHMP2B^Intron5^* mutation leading to the production of the truncated form of the protein with missing C-terminal residues 179–213 [229]) are linked to the pathogenesis of frontotemporal dementia (FTD) associated with frontotemporal lobar degeneration (FTLD) and amyotrophic lateral sclerosis (ALS) [230,231]. Since there is a significant clinical, genetic, and neuropathological overlap between ALS and FTD, this represents a continuum of a single ALS-FTD spectrum disorder [222]. Furthermore, Parkinsonian syndrome was described in familial FTD in families with mutations in the *CHMP2B*, as well as chromosome 9 open reading frame 72 (C9ORF72), fused in sarcoma (FUS), microtubule-associated protein tau (*MAPT*), progranulin (*PRGN*), transactive DNA-binding protein (TARDBP), and valosin-containing protein (VCP) [232] genes.

CHMP2B includes two N-terminal coiled–coil regions (residues 1–50 and 120–150) and a C-terminal MIT-interacting motif (MIM, residues 201–211) critical for interacting with vacuolar protein sorting-associated protein 4 (Vps4) and other proteins containing microtubule interacting and transport (MIT) domains [222,233]. It was shown that CHMP2B can self-polymerize into helical complexes (likely via coiled–coil regions) capable of deforming membranes [234]. However, the polymerization is typically autoinhibited via interaction between the MIM-containing acidic C-terminus and the basic N-terminus [234]. Importantly, binding of Vps4 to the MIM of CHMP2B releases autoinhibition of the protein, thereby initiating its polymerization [234].

Despite the crucial importance of this protein for various physiological and pathological processes and conditions, structural information on human CHMP2B is limited to the NMR structure of the MIM motif (residues 195–213) bound to human VPS4B (PDB ID: 2JQK [235]). Figure A2A,B provide a logical explanation of this phenomenon by showing that human CHMP2B is expected to be mostly disordered. However, this disorder could be of functional importance, as CHMP2B is predicted to have 6 MoRFs (residues 1–7, 54–69, 96–101, 141–151, 162–180, and 206–213), with the last MoRF overlapping with the MIM motif. In other words, 67 of the 213 residues of CHMP2B (31.5%) form disorder-based binding platforms, indicating that this protein can be a promiscuous binder. This hypothesis is supported by Figure A2C, which shows the CHMP2B-centered PPI network that includes 139 nodes (proteins) connected by 2694 edges (interactions) and is characterized by an average node degree of 38.8 and an average local clustering coefficient of 0.732. With an expected number of edges of 425, this network has significantly more interactions than expected (PPI enrichment *p*-value: <1.0 × 10^−16^). One of the members of this CHMP2B-centered PPI network is α-synuclein, which itself is involved in interaction with 46 CHMP2B interactors. Importantly, the FuzDrop-based analysis revealed that CHMP2B cannot undergo spontaneous LLPS but acts as a droplet client, since although it is characterized by the probability of spontaneous liquid–liquid phase separation below the 0.6 threshold (p_LLPS_ = 0.4588), it has two droplet-promoting regions (DRPs, residues 107–118 and 184–198).

### Appendix A.3. NRGN (Neurogranin, UniProt ID: Q92686; PPIDR_PONDR_^®^ _VSL2_ = 100.0%; ADS _PONDR_^®^ _VSL2_ = 0.8643)

Neurogranin (NRGN), a 78-residue-long multifunctional protein from the calpacitin family, which is also known as b50-immunoreactive C kinase substrate (BICKS), RC3, and P17, is involved in the plasticity and regeneration of synapse mediated by the calcium- and calmodulin-signaling pathways [236]. This protein is preferentially found in the perikarya and dendrites of advanced differentiated neurons, as well as in the neuronal nuclei of the cerebral cortex [236]. Similar to another member of the calpacitin protein family, growth-associated protein-43 (GAP-43), NRGN is involved in long-term potentiation (LTP) and the elaboration of pre- and postsynaptic structures. The protein got its name “neurogranin” based on the fact that it is typically expressed in granule-like structures in pyramidal cells of the hippocampus and cortex [237]. This forebrain-enriched, postnatal-onset, thyroid hormone-dependent protein is known to serve as a selective substrate for protein kinase C (PKC) [238]. Unphosphorylated NRGN/RC3 interacts with calmodulin (CaM) in a Ca^2+^-dependent manner [239] and plays a role in adult neural plasticity and neonatal synaptogenesis, being involved in the Ca^2+^-mediated second messenger cascades [240]. It was hypothesized that such bimodality of Ca^2+^-“sensitive” interaction between RC3 and CaM modulates Ca^2+^/CaM availability, thereby regulating the transduction of the postsynaptic Ca^2+^ fluxes into the physiological responses [240]. Interaction between NRGN and CaM is driven by the IQ motif (residues 26–47) containing the PKC target residue Ser36, phosphorylation of which abrogates NRGN–CaM interaction [241,242].

Deregulation of this protein is linked to the pathogenesis of multiple neurological and mental diseases, such as Alzheimer’s disease (AD), acute ischemic stroke (AIS), Creutzfeldt–Jakob disease (CJD), depression, first episode psychosis (FEP), Huntington disease (HD), mild cognitive impairment (MCI), neuro-HIV, neurosyphilis (NS), Parkinson’s disease (PD), traumatic brain injury (TBI), and schizophrenia [236]. For example, both AD and MCI are characterized by high NRGN levels in cerebrospinal fluid (CSF) [243,244,245,246,247]. The CSF levels of this protein were shown to correlate with the cognitive decline in AD [248], and higher CSF NRGN levels were shown to positively correlate with the higher scores of tau tangle pathology and Aβ neuritic plaques [249]. In the progressive MCI group, accelerated cognitive deterioration was shown to correlate with elevated CSF NRGN levels [247]. In CJD as well, highly elevated NRGN levels were found in CSF [250]. On the contrary, the CSF NRGN levels were significantly decreased in PD, PD with MCI, and PD with dementia (PDD) [249]. Similarly, *NRGN* was shown to be one of the most robustly down-regulated genes in HD [251,252]. Curiously, NRGN together with α- and β-synuclein, as well as visinin-like protein 1 (VILIP-1) and neuronal pentraxin 2, are now considered fluid AD biomarkers [253,254], with neurogranin, α-synuclein, and β-synuclein being considered potential biomarkers for synaptic dysfunction in neurodegenerative diseases [255,256].

Multiparametric experimental analysis of the NRGN fragment (residues 28–43) corresponding to the CaM binding IQ motif and containing Ser36 residue targeted by PKC revealed that in aqueous solution, this peptide existed preferentially in the random coil state but underwent transition to a-helical form in the presence of sodium dodecyl sulfate (SDS) micelles or organic solvents [257]. Using triple resonance NMR techniques, it was shown that in the unbound form, the full-length rat NRGN is mostly unfolded in the unbound form and contains a residual structure in the form of the nascent local α-helical region between residues 25–42 [258]. In line with these observations, Figure A3A,B shows that human NRGN is predicted as a mostly disordered protein containing four MoRFs (residues 1–6, 14–24, 26–47, and 64–72), one of which overlaps with the CaM binding IQ motif (see Figure A3A) and is predicted as an α-helix by AlphaFold (Figure A3B). In other words, 48 residues (61.5%) of this protein are expected to be engaged in disorder-based protein–protein interactions, which can be controlled by PTMs (see Figure A3A). Therefore, it is not surprising to find that NRGN forms a very dense PPI network containing 418 nodes connected by 25,430 edges (an expected number of edges is 8111) (see Figure A3C). The average node degree of this network is very high, as, on average, each member is expected to interact with 122 in-network partners. Furthermore, this network contains 82 members that interact with more than 200 partners each, and 212 members have at least 122 partners each. Importantly, human neurogranin was predicted to have a very high probability of spontaneous liquid–liquid phase separation (p_LLPS_ = 0.9722) and possess a long C-terminally located DPR (residues 38–78), which also includes an aggregation hotspot (residues 38–48), suggesting that this protein is capable of spontaneous LLPS and can potentially drive the aggregation of condensates.

### Appendix A.4. CPLX1 (Complexin-1; UniProt ID: O14810; PPIDR_PONDR_^®^ _VSL2_ = 100.0%; ADS _PONDR_^®^ _VSL2_ = 0.8819)

Complexin-1 (CPLX1 or CPX1, also known as synaphin-2) is a member of a family of two closely related proteins (complexins 1 and 2) that were originally discovered as proteins interacting with SNARE (soluble N-ethylmaleimide sensitive factor attachment protein receptor) [259,260,261]. The soluble and insoluble forms of complexins are enriched in synapses [261,262,263], where they may act as negative regulators of neurotransmitter release [262,264] at a step immediately preceding vesicle fusion [265]. The interaction of complexins (together with synaptotagmins) with SNAREs regulates conformational changes within the SNAR proteins associated with Ca^2+^-triggered exocytosis [266]. This study also revealed that for synaptotagmin-Ca^2+^ to trigger synaptic fusion, the conformational switch from open to closed in complexin is required [266].

Similar to many other intrinsically disordered proteins (IDPs), complexins are characterized by broad multifunctionality. This prompted Justine A. Lottermoser and Jeremy S. Dittman to state in their recent review that one of the key synaptic proteins, complexin, lives up to its name, being a small but complex and poorly understood protein with a variety of functional roles in synapses and fusion machinery [267]. In relation to the subject of this study, it was shown that changes in the brain levels of CPLX1 are associated with the α-synuclein pathology in the mouse brain [268].

Despite its diminutive size (human CPLX1 contains 134 residues), this protein has four functional domains [267,269]: N-terminal domain (NT, residues 1–28), which is involved in interaction with SNAP25 and membrane binding as well as may support the fusogenic activity of CPLX1 [269,270,271,272,273,274]; the accessory helix domain (AH, residues 29–47) required for CPLX1-driven inhibition of fusion [269,274]; central helical domain (CH, residues 48–69), responsible for tight binding to the assembled SANARE proteins and required for all known CPLX1 functions [275,276,277]; and the poorly conserved C-terminal domain (CT, residues 70–134) involved in membrane interactions required for the proper localization of CPLX1 relative to SNAP and syntaxin-1 and related to membrane fusion [267,278,279,280,281,282,283].

Solution NMR analysis revealed that purified recombinant rat complexin-1 lacks a tertiary structure but contains a conserved α-helical middle region, where a stable α-helix is found in the 29–64 region, whereas residues 65–86 contain a substantial but lower population of α-helix [275]. In line with these observations, Figure A4A,B show that a highly disordered human CPLX1 contains 6 MoRFs (residues 1–20, 51–57, 64–72, 84–89, 100–105, and 115–134), three of which overlap with the aforementioned helical regions. STRING-generated PPI-network centered at human CPLX1 includes 378 proteins connected by 14,779 interactions and is characterized by an average node degree of 78.2 and an average local clustering coefficient of 0.604 (see Figure A4C). Based on the outputs of FuzDrop, human complexin-1 is characterized by a p_LLPS_ of 0.9678, three DPRs (residues 1–35, 42–69, 86–104, 38–78), and five aggregation hotspots (residues 30–35, 49–55, 62–68, 86–91, and 94–99), suggesting that this protein is capable of spontaneous LLPS and can potentially drive the aggregation of condensates.

### Appendix A.5. CPLX2 (Complexin-2; UniProt ID: Q6PUV4; PPIDR_PONDR_^®^ _VSL2_ = 100.0%; ADS _PONDR_^®^ _VSL2_ = 0.9135)

Complexin-2 is a second member of the human complexin family. These proteins share 84.3% of their sequence identity and show a sequence similarity of 91.8%. Therefore, it is not surprising that CPLX2 was shown to interact with the SNARE complex and thereby regulate the Ca^2+^-triggered fusion between vesicles and the plasma membrane [284]. However, although CPLX1 is preferentially expressed in the brain, CPLX2 is found in the brain and in some secretory cells [260,261], including pancreatic secretory cells [285] and peripheral mast cells [286], where it participates in the Ca^2+^-dependent degranulation through syntaxin 3 [286]. Furthermore, CPLX2 can be expressed in B lymphocytes and regulates the secretion of immunoglobulin in antibody-secreting cells [287]. It was also shown that CPLX2 participates in docking, locking, and unlocking of different SNARE complexes during sperm capacitation and induced acrosomal exocytosis [288]. Immunocytochemical analyses of the frontal cortex of HD patients revealed a significant reduction in CPLX2 levels in comparison with the HD presymptomatic patients, which seemed to correlate with the pathological grade of the disease [289].

Comparison of the data in Figure A4A and Figure A5A, as well as Figure A4B and Figure A5B, indicates that, in line with their high sequence similarity, human CPLX1 and CPLX2 possess similar levels of disorder. Being highly disordered, human CPLX2 has 5 MoRFs (residues 1–21, 65–72, 85–94, 97–104, and 115–134) and several PTMs. Figure A5C represents the PPI network centered at the human CPLX2. This STRING-generated network includes 348 proteins connected by 17,425 interactions. It is characterized by an average node degree of 100 and an average local clustering coefficient of 0.649. Finally, FuzDrop analysis showed that human CPLX2 is a bit more prone to spontaneous LLPS than CPLX1, as its p_LLPS_ is 0.9811. It has one long DPR (residues 1–110) that covers more than 82% of its sequence and two aggregation hot spots (residues 59–66 and 83–109).

### Appendix A.6. NUCKS1 (Nuclear Ubiquitous Casein and Cyclin-Dependent Kinase Substrate 1; UniProt ID: Q9H1E3; PPIDR_PONDR_^®^ _VSL2_ = 100.0%; ADS _PONDR_^®^ _VSL2_ = 0.9879)

Nuclear ubiquitous casein and cyclin-dependent kinase substrate 1 is a 243-residue-long chromatin-associated protein that is involved in DNA repair by homologous recombination (HR, a DNA repair pathway critical for tumor suppression) and chromosome stability [290]. This protein is known to bind to double-stranded DNA (dsDNA) and can also interact with secondary DNA structures, such as D-loop structures [290]. NUCKS1 is highly expressed in a variety of malignant tumors, such as breast cancer [291,292], hepatocellular carcinoma [293,294], ovarian cancer [295], gastric cancer [296], and cervical squamous cell carcinoma [297], and is believed to function as an oncogen [298]. This protein was shown to promote the progression of colorectal cancer by activating the PI3K/AKT/mTOR signaling pathway [298]. In osteosarcoma, NUCKS1 elevates asparagine synthesis by transcriptionally upregulating asparagine synthetase (ASNS) expression, thereby promoting osteosarcoma progression and metastasis [299]. In lung adenocarcinoma, upregulation of NUCKS1 is associated with a poor prognosis [300].

In HIV-1 infection, NUCKS1 acts as a Tat activator and plays a crucial role in HIV-1 replication by enhancing Tat-mediated viral transcription on the HIV-1 LTR promoter [301]. This protein can also serve as a biomarker of metabolic disease since NUCKS protein levels are inversely correlated with body mass index in humans [302]. Some genetic variants in NUCKS1 are associated with sporadic Parkinson’s disease in Han Chinese [303]. Being located within the PARK16 gene locus, which possibly regulates PD risk, NUCKS1 represents a potential PD susceptibility biomarker [304]. Genetic polymorphism of NUCKS1 is associated with the susceptibility of adolescent idiopathic scoliosis [305].

NUCKS1, being similar to the HMG (high-mobility group) protein family, is one of the most modified proteins in the mammalian proteome [302]. In fact, it was shown that the NUCKS1 protein can be phosphorylated at ~25 different residues [306,307]. It was also shown that in solution, NUCKS1 does not contain defined structure and shows a very low content of α-helical and β-structural, instead containing a relatively high proportion of β-turns [308].

In agreement with the aforementioned experimental structural analysis of human NUCKS1, Figure A6A,B shows that this protein is predicted to be almost completely disordered. It contains 5 MoRFs (residues 1–27, 32–40, 89–113, 124–157, and 170–197) and is heavily decorated by numerous various PTMs (see Figure A6A). According to AlphaFold, it almost does not contain elements of an ordered secondary structure. The exception is given by residues 13–19 and 96–108, which show some helical propensity and overlap with two MoRFs (Figure A6B). Based on the results of STRING analysis, NUCKS1 is involved in the formation of a PPI network containing 366 proteins connected by 9275 interactions (see Figure A6C). Since the expected number of edges is 5028, this NUCKS1-centered network has significantly more interactions than expected (PPI enrichment *p*-value <1.0 × 10^−16^). The average node degree of this network is 50.7, and its average local clustering coefficient is 0.51. FuzDrop analysis revealed that human NUCKS1 has a probability of spontaneous liquid–liquid phase separation of 0.9945, with the entire sequence acting as one long DPR. Furthermore, this protein has an impressive set of aggregation hotspots that are located at residues 8–13, 20–27, 31–40, 54–72, 79–110, 115–128, 135–140, 144–159, 184–190, and 205–213.

### Appendix A.7. MBP (Myelin Basic Protein; UniProt ID: P02686; PPIDR_PONDR_^®^ _VSL2_ = 100.0%; ADS _PONDR_^®^ _VSL2_ = 0.8706)

Myelin basic protein (MBP) and proteolipid protein (PLP) are two major protein components of the myelin sheath of the central nervous system (CNS) [309,310,311,312], which represents an insulation of the nerve fibers formed by the membranes extending from oligodendrocytes and wrapping multiple times around the nerve fibers required for facilitation of the rapid transmission of nerve impulses [313]. Deficiencies in myelin assembly and structure are associated with various neurological diseases [312,313,314]. For example, MBP immunoreactivity was found in the core of LBs in the brainstem, cingulate cortex, and sympathetic ganglia of patients with PD and dementia in LBs patients [315]. Multiple system atrophy (MSA) pathogenesis is linked in part to the dysfunction of α-synuclein and myelin proteins [316].

In humans, differential splicing of a single mRNA transcript generates four MBP isoforms: 21.5, 20.2, 18.5, and 17.2 kDa [313]. Although MBP exists in multiple isoforms, in humans, one of the most abundant proteins of the myelin sheath is known as the “classic” 18.5 kDa isoform [313,317]. All isoforms of MBP are IDPs [313,318,319]. The intrinsically disordered nature of this protein made it non-crystallizable. In fact, in their comprehensive search for a suitable composition of a crystallization medium, Jan Sedzik and Daniel A. Kirschner tried 4600 different conditions but failed to induce MBP crystallization [320]. Based on these observations, the authors concluded that 18.5 kDa MBP and its isoforms represent proteins that cannot be crystallized [320].

Figure A7A,B shows that human MBP is predicted to be almost completely disordered. It contains 10 MoRFs that cover 73% of its sequence (residues 1–16, 30–52, 59–67, 70–101, 116–139, 141–185, 198–209, 218–229, 240–255, and 258–292) and is heavily modified by phosphorylation and methylation (see Figure A7A). According to AlphaFold, human MBP contains a very limited amount of the elements of ordered secondary structure. In fact, there are two short α-helical segments (residues 171–180 and 218–228), one of which is included in the MoRF spanning residues 141–185, and another overlaps with 218–229 MoRF (Figure A7B). The STRING-generated PPI network centered at MBP includes 422 proteins connected by 19,398 interactions (see Figure A7C). An expected number of interactions for a random set of proteins of the same size and degree distribution drawn from the genome is 8971, indicating that this network has significantly more interactions than expected (PPI enrichment *p*-value <1.0 × 10^−16^). The average node degree of this network is 91.9, and its average local clustering coefficient is 0.646. FuzDrop analysis revealed that human MBP is characterized by a p_LLPS_ of 0.9903 and contains 4 DPRs (residues 1–89, 135–145, 165–219, and 228–304) and 7 aggregation hotspots (residues 36–46, 179–184, 192–200, 208–218, 232–238, 259–267, and 278–298).

### Appendix A.8. CAST (Calpastatin; UniProt ID: P20810; PPIDR_PONDR_^®^ _VSL2_ = 100.0%; ADS _PONDR_^®^ _VSL2_ = 0.9547)

Calpastatin (also known as Calpain inhibitor or Sperm BS-17 component) is a 708-residue-long protein acting as a specific inhibitor of the Ca^2+^-dependent cysteine protease calpain. The interest in CAST is determined by its ability to act as a specific endogenous protein inhibitor, modulating calpain activity. Due to their critical involvement in apoptosis, aging, and neurodegeneration (e.g., AD pathogenesis), the non-lysosomal cysteine proteases calpains are studied very well (as of April 2024, there were more than 10,720 papers dedicated to calpain in PubMed). One of the peculiar features of the proteolytic activity of calpains is their dependence on the tertiary structure features of the protein substrates rather than their specific primary amino acid motifs. As a result, instead of breaking down target proteins into small fragments or amino acids, calpains cleave at highly selective sites [321]. This feature helps calpains regulate specific enzymes (such as Ca^2+^-dependent kinases and phosphatases, calcineurin, calcium ATPase, Ca^2+^-dependent cyclic nucleotide phosphodiesterase, tyrosine hydroxylase, CAMP-dependent protein kinases, phosphorylase kinase, and glycogen synthase) by specific cleavage between regulatory and catalytic domains, both in a Ca^2+^-dependent and Ca^2+^-independent manner [321]. In humans, there are more than a dozen calpain isoforms, some with multiple splice variants [322,323]. Depending on their calcium requirements for physiological functions, calpains are grouped into two major types, μcalpain (μCANP or Calpain I) and mcalpain (mCANP or Calpain II), that have optimal activities at calcium concentrations in the low micromolar or nearly millimolar levels, respectively [324,325,326,327]. Furthermore, calpains have a crucial Ca^2+^ level-dependent transition from regulators to destroyers [321], acting at the physiologic calcium levels as coordinators of a multitude of signaling pathways that control diverse intracellular proteins and organelles at the membrane-cytoskeleton interface [328,329,330] or as vicious destructors capable of cleaving more than half of the cell’s protein pools in 1 h [331].

Calpastatins represent a family of isoforms derived from a single *CAST* gene by alternative mRNA splicing [332], PTMs (preferentially phosphorylation) [333], and proteolysis [334]. These isoforms show a tissue-specific distribution and range in molecular mass from 7 to 140 kDa [321]. Typical CAST contains four equivalent inhibitory domains (I, II, III, and IV), each capable of inhibiting a separate calpain molecule [335]. As a result, calpastatin was defined as a multiheaded inhibitor capable of inhibiting more than one calpain molecule [336]. In the canonical form of human CAST, these inhibitory domains are located at residues 137–277, 278–326, 427–563, and 564–708. Each of these inhibitory domains contains three conserved subdomains, A, B, and C, which are located at residues 170–222, 304–356, 446–499, and 583–636, and are primarily responsible for the calpain inhibition. Inhibition of calpain is potentiated by the subdomains A and C that interact with the enzyme in a Ca^2+^-dependent fashion [337,338].

Combined analysis of the inhibitory domain I of human CAST using ^1^H-NMR and circular dichroism (CD) revealed that this domain did not have any ordered structure in solution [339]. Similarly, a comprehensive structural characterization of the pig calpastatin domain I revealed that at neutral pH, this domain is in an expanded and flexible conformation without secondary and tertiary structures [340]. A full NMR assignment of the CAST inhibitory domain I (residues 137–277) revealed that although this domain is mostly disordered in the unbound form, it retains some residual transient structure [341]. In fact, regions with helical propensity were found within all three subdomains of domain I: residues 18–25 within subdomain A (residues 12–30), 51–59 and 68–75 within subdomain B (residues 50–70), and residues 91–104 within subdomain C (residues 87–105) [341].

Our bioinformatics analysis of human CAST provides strong support for the idea that this important protein has very high levels of intrinsic disorder. Figure A8A shows that CAST is predicted to be mostly disordered by all the predictors included in the D^2^P^2^ platform. Furthermore, this protein is predicted to have 20 MoRFs covering 64.5% of its sequence (residues 1–33, 39–52, 64–89, 103–124, 132–141, 150–166, 182–215, 226–246, 258–270, 286–316, 333–347, 366–379, 397–444, 476–488, 491–499, 502–551, 564–581, 593–629, 647–662, and 673–685) and a multitude of various PTMs (see Figure A8A). The AlphaFold-predicted 3D structural model of this protein includes several short α-helices that do not form a hydrophobic core (Figure A8B). The CAST-centered PPI network generated by STRING includes 279 proteins engaged in 9487 interactions. The averaged node degree of this network is 68, and it has an average local clustering coefficient of 0.66 (see Figure A8C). High levels of intrinsic disorder combined with the prevalence of disorder-based interaction sites are likely related to the extremely high probability of spontaneous liquid–liquid phase separation (p_LLPS_ = 0.9989). With its three very long DPRs (residues 1–414, 415–532, and 537–708) and with 22 aggregation hotspots spread through the entire sequence, CAST is not only expected to be extremely prone to spontaneous LLPS but can also trigger the formation of aggregates within phase-separated droplets.

### Appendix A.9. MAPT (Microtubule-Associated Protein Tau; UniProt ID: P10636; PPIDR_PONDR_^®^ _VSL2_ = 99.1%; ADS _PONDR_^®^ _VSL2_ = 0.8612)

The microtubule-associated protein (MAP) tau is one of a group of MAPs that, in addition to the presence of various tubulin isoforms subjected to a broad spectrum of different PTMs, are involved in controlling the assembly/disassembly, functionality, morphology, and stability of the essential constituents of the cytoskeleton in eukaryotic cells, microtubules (MTs) [342]. The primary function of tau is MT stabilization via its binding to MTs in a tau phosphorylation-dependent manner [343]. Under pathological conditions, hyperphosphorylation of tau reduces its affinity to MTs, causing the abnormal detachment of tau from the MTs that leads to axonal transport defects [344] and triggers misfolding and aggregation of tau [345,346] that eventually results in the formation of the intracellular filamentous inclusions neurofibrillary tangles (NFTs) found in AD and other neurodegenerative disorders [347]. Therefore, to everyone who is even very superficially familiar with AD, the microtubule-associated protein tau does not require a special introduction, as this IDP is one of the most studied molecular drivers of AD. This fact is supported by more than 37,500 papers dedicated to this protein found in PubMed (as of April 2024). Importantly, AD is not the only neurodegenerative pathology associated with the misbehavior of tau. These neurodegenerative maladies are known as primary tauopathies. They represent a heterogeneous group of disorders that are driven by the misfolding and abnormal aggregation of filamentous tau to form different pathological inclusions [347,348,349,350,351,352]. The accumulation of these inclusions leads to degeneration within the afflicted brain regions. This gives rise to the specific clinical impairments reflected in a broad spectrum of behavioral, cognitive, and motor symptoms [350,353]. Similar to prion protein, α-synuclein, and many other proteins related to the various forms of amyloidosis, tau is capable of the formation of conformationally distinct pathological protein aggregates, or strains. Furthermore, these pathological aggregates can be transmitted between the anatomically connected brain regions, resulting in the spread of pathological protein inclusions [354,355]. Histopathological characteristics, such as the temporal distribution, morphology, and affected cell types, form the foundation for subcategorization of tauopathies into several diseases [354]. Furthermore, depending on the tau isoform found in the inclusions, the major tauopathies can also be subdivided into 3R, 4R, and 3R/4R tauopathies [354].

Human tau protein is known to exist as a mixture of multiple isoforms, which are produced by alternative splicing and which differ from each other by the presence or absence of up to 5 of the 15 exons. The longest isoform includes 758 residues and has been chosen as a canonical form. This isoform includes a long IDR (residues 1–573) and a microtubule-binding domain (residues 561–685) possessing four tandem repeats of a conserved tubulin-binding domain (residues 561–591, 592–622, 623–653, 654–685) [356]. The most common isoform of tau is known as Tau-F (also known as Tau-4 or 2N4R [357], which contains 441 residues and is different from the canonical form by missing residues 125–375 and 395–460). Solution NMR analysis revealed that this tau isoform, as well as two shorter isoforms (with 383 and 352 residues), are all typical IDPs with a very limited and highly dynamic residual secondary structure [358]. It was also shown that the structure of the 2N4R is highly dynamic and polymorphic but yet shows a distinct domain character with a sophisticated network of transient long-range contacts crucial for pathogenic aggregation [359].

In agreement with these experimental observations, Figure A9 shows that the canonical isoform of tau is predicted to be a highly disordered protein (Figure A9A) with almost non-existent elements of flexible secondary structure (Figure A9B). Furthermore, in line with the aforementioned crucial dependence of the physiological and pathological behavior of this protein on phosphorylation, Figure A9A shows that human tau (especially its microtubule-binding domain) is heavily decorated by PTMs. Figure A9A also shows that almost the entire sequence of this protein can serve as a disorder-based platform for protein–protein interactions, suggesting its high binding promiscuity. This hypothesis is supported by Figure A9C, which shows that the tau-centered PPI network includes 337 proteins connected by 5794 interactions. This STRING-generated network is characterized by an average node degree of 34.4 and an average local clustering coefficient of 0.611. Recently, it was shown that human tau is capable of spontaneous LLPS and preserves its mostly disordered nature in the droplet state, with repeat regions attaining transient β-hairpin propensity upon LLPS [360,361,362]. It was also pointed out that, similar to α-synuclein, fused in sarcoma (FUS), and the transactive response DNA-binding protein of 43 kDa (TDP-43), the biomolecular condensates formed by tau may “mature”/”age,” i.e., undergo a liquid-to-solid phase transition accompanied by the formation of amyloid fibrils, oligomeric species, or amorphous aggregates, thereby contributing to the pathology of various forms of neurodegeneration [363]. Our sequence-based bioinformatics analysis using the FuzDrop platform revealed that tau is indeed characterized by very high LLPS potential (p_LLPS_ = 0.9985) and has four DPRs (residues 1–300, 309–589, 608–622, and 719–739) that include numerous aggregation hotspots (residues 2–12, 113–125, 128–133, 139–145, 186–195, 265–278, and 290–295 within the DPR1, residues 309–315, 454–461, 539–546, 562–574, and 578–589 within the DPR2, as well as residues 608–620 and 719–739 within the DPRs 3 and 4, respectively).

Finally, in line with the well-established pathological and functional cross-talks of α-synuclein and tau in the central nervous system (e.g., co-occurrence of α-synuclein and tau aggregates in the post-mortem brains with synucleinopathies and tauopathies and the overlapping clinical symptoms of dementia and parkinsonism [364,365,366,367], molecular interactions and cross-seeding between α-synuclein and tau in neurodegenerative diseases [368,369,370], as well as functional cooperation of α-synuclein and tau during proper brain development via maintenance of progenitor cells [371]), STRING analysis indicated that the tau-centered PPI network includes α- and β-synucleins that interact with 161 and 17 members of this network, respectively.

### Appendix A.10. HEMGN (Hemogen; UniProt ID: Q9BXL5; PPIDR_PONDR_^®^ _VSL2_ = 98.3%; ADS _PONDR_^®^ _VSL2_ = 0.8304)

Hemogen, also known as erythroid differentiation-associated gene protein (EDAG-1, which was previously designated as embryonic development-associated gene 1), hemopoietic gene protein, or negative differentiation regulator (NDR) protein, is a 484-resodue-long hematopoietic tissue-specific transcription regulator involved, which is specifically expressed in hematopoietic cells and in regulation of proliferation, differentiation, and apoptosis of hematopoietic cells [372,373,374,375,376]. It was shown that alternative promoters and polyadenylation of the *HEMGN* gene lead to the biosynthesis of at least two distinct splicing variants in hematopoietic cells and in round spermatids in the testis, suggesting a role for this protein in spermatogenesis [377]. Furthermore, hemogen was shown to be related to the pathogenesis of erythroleukemia and megakaryoblast leukemia [378], its overexpression is associated with poor prognosis in de novo acute myeloid leukemia (AML) [376], and it can promote proliferation and invasion of human thyroid cancer cells by activating MAPK/Erk and AKT signal pathways [379].

No structural information is available for hemogen as of yet. Therefore, the results of the bioinformatics analysis reported in Figure A10 provide a unique description of this important protein. Figure A10A shows that hemogen is expected to be mostly disordered by the most predictors utilized in D^2^P^2^ and is predicted to contain 17 MoRFs. The fact that this protein contains multiple PTM sites suggests that its activity is controlled by posttranslational modifications. Figure A10B provides further support for the mostly disordered nature of human hemogen and shows that, as per AlpgaFold, this protein is expected to have just one N-terminally located α-helix (residues 30–66), which is included in the region necessary for nuclear localization (residues 7–87). The hemogen-centered PPI network generated by STRING includes 373 proteins engaged in 7813 interactions. The averaged node degree of this network is 41.9, and it has an average local clustering coefficient of 0.52 (see Figure A10C). One of the members of this network is α-synuclein, which is involved in interaction with hemogen and 127 hemogen interactors. Finally, FuzDrop-based analysis showed that human hemogen has a high LLPS potential (p_LLPS_ = 0.9935) and is predicted to have four DPRs (residues 1–31, 41–92, 97–126, and 261–475) that include numerous aggregation hotspots (residues 86–92, 120–126, 194–202, 261–288, 291–300, 307–344, 348–358, 392–401, 404–414, 442–251, and 454–464) mostly concentrated with the C-terminally located longest DPR. However, DPRs 2 and 3 have short aggregation hotspots as well.

### Appendix A.11. H1.2 (Histone H1.2; UniProt ID: P16403; PPIDR_PONDR_^®^ _VSL2_ = 97.7%; ADS _PONDR_^®^ _VSL2_ = 0.8947)

Histone H1.2 is a 213-residue long linker histone that is responsible for condensation of the nucleosome chains into the higher-order structured chromatin fibers. It is one of the seven H1 variants found in human somatic cells (H1.1 to H1.5, H1.0, and H1X) [380,381,382]. It is believed that H1 variants are specifically distributed among different cell lines [382,383,384,385] and have different functional repertoires, being able to act as general repressors and also possessing variant-specific functional diversity in chromatin regulation [380,381,382]. It was emphasized that the proportions of H1 variants present in a specific cell (i.e., H1 cell complement) differ between the cell types and are also dynamically changed throughout differentiation and cancer [382]. In fact, chromatin structural defects are known to be caused by compromised H1 content [386]. A recent super-resolution microscopy-based analysis of several human cell lines revealed that somatic H1 histones are characterized by a differential nuclear distribution [382]. The authors pointed out that the nuclear periphery and more compacted DNA are enriched in H1.2, H1.3, H1.5, and, to a lesser extent, H1.0, whereas H1X and H1.4 are distributed throughout the nucleus, with nucleoli being significantly enriched in H1X [382]. Based on these observations, they concluded that different H1 variants have diverse implications for genome functionality. It was also emphasized that the chromatin structure can be affected by H1 variant depletion in a variant-specific manner, with a global chromatin decompaction being triggered by a H1.2 knockdown [382]. A detailed description of H1.2 functionality and its crucial roles in the maintenance of genome stability, apoptosis, and cell cycle regulation, as well as its association with disease, is provided in a recent comprehensive review [387].

Structurally, human H1.2 histone is characterized by the presence of disordered N- and C-terminal regions (residues 1–41 and 92–213, respectively) and a linker histone H1/H5 globular (H15) domain (residues 36–109). In line with these observations, circular dichroism-based analysis of the murine H1.2 linker histone revealed that although its structure was dominated by the random coil, some α-helical components belonging to the stably folded globular domain were also present [388]. An earlier comprehensive bioinformatics analysis of 2007 histones from 746 species revealed that all the analyzed members of the histone family are intrinsically disordered proteins and that their copious intrinsic disorder is absolutely necessary for various functions of these proteins [389].

Figure A11A shows that according to the predictive tools assembled into the D^2^P^2^ platform, the human linker H1.2 histone is predicted to have high levels of intrinsic disorder in the N- and C-terminal regions and also possess a more ordered structure in the central region. This is further supported by Figure A11B, which shows the modeled 3D structure of this protein, where the presence of a helical globular domain (residues 40–107) is evident. Similar to other histones, the linker histone H1.2 is heavily decorated by a wide spectrum of different PTMs, such as acetylation, formylation, methylation, PARylation, phosphorylation, and ubiquitination [387,390,391,392,393,394]. Figure A11A supports these observations and shows that the entire protein is densely covered by various PTMs. Furthermore, human H1.2 histone is predicted to have 6 MoRFs (residues 42–49, 56–70, 79–94, 103–112, 132–145, and 151–213) (see Figure A11A).

Involvement of this protein in multiple interactions is illustrated by Figure A11C showing the STRING-generated PPI network, where 339 proteins, connected by 16,477 interactions, form a dense interactome with an average node degree of 97.2 and an average local clustering coefficient of 0.622. Importantly, it was established that incubation of the murine H1.2 with the 1–42 Amyloid-β peptide helped the folding of Aβ monomers, promoted the formation of laminar aggregates and thick bundles, and stabilized the parallel association of fibrils [388]. Similarly, it was shown that the linker histone H1 (as well as other histones) can interact with human α-synuclein, forming a tight 2:1 complex and dramatically accelerating the fibrillation rate of α-synuclein in vitro [395]. Furthermore, in mice exposed to a toxic insult (i.e., injections of the herbicide paraquat), α-synuclein was found in the nucleus, where it was co-localized with the histones in the nuclei of nigral neurons [395].

The analysis of the human linker H1.2 histone by FuzDrop revealed that this protein has a very strong predisposition to spontaneous LLPS (it is characterized by a p_LLPS_ of 0.9966). Both the N- and C-terminal regions of this protein were also predicted as DPRs (residues 1–53 and 112–213, respectively). Furthermore, the human linker H1.2 histone was predicted to have five aggregation hotspots: residues 21–27, 38–43, 96–103, 112–124, and 161–171. Curiously, with the exception of the last hotspot, which is located within the last MoRFs, other aggregation hotspots are mostly positioned between the MoRFs.

## Appendix B. Functionality and Intrinsic Disorder of Some Most Ordered Proteins from the Joint α-β-γ-Synuclein Interactome

### Appendix B.1. Prolyl Endopeptidase (PREP, UniProt ID: P48147)

Prolyl endopeptidase (PREP) is a cytosolic, 710-residue-long member of the family of serine peptidases. Since it cleaves peptide bonds on the C-terminal side of prolyl residues, it is also known as a post-proline-cleaving enzyme. It also has the name prolyl oligopeptidase, as it targets oligopeptides of less than 10 kDa. PREP is involved in the maturation and degradation of various peptide hormones and neuropeptides, such as α-melanocyte-stimulating hormone, angiotensin, luteinizing hormone-releasing hormone (LH-RH), neurotensin, oxytocin, substance P, thyrotropin-releasing hormone, and vasopressin [396,397]. Although various peptides are targeted by this endopeptidase, an intriguing feature of PREP is the absolute requirement for the trans-configuration of the peptide bond preceding proline [398]. Figure A12 shows that humans are highly ordered proteins, which is evident from the outputs of RIDAO analysis (Figure A12A) and the well-defined 3D structure solved to a high resolution of 1.56 Å (PDB ID: 3DDU) [399] (see Figure A12D). Figure A12B shows that human PREP shows low LLPS potential but contains one DPR (residues 188–206), suggesting that this protein might act as a droplet client. The PREP-centered PPI network generated by STRING includes 70 nodes connected by 418 edges (see Figure A12C). Therefore, this network is characterized by an average node degree of 11.9 and an average local clustering coefficient of 0.693. The most statistically significant biological processes, molecular functions, and cellular components ascribed to the members of this network are listed in Table A1.

**Table A1:** Functional enrichment of the five mostly ordered proteins from the STRING-generated joint PPI network centered at human α-, β-, and γ-synucleins.

Protein	ID	Description	Order of Magnitude of the *p*-Value
**Prolyl endopeptidase**	**Biological Process (Gene Ontology)**		
	GO:0006508	Proteolysis	−30
	GO:0019538	Protein metabolic process	−17
	GO:1901564	Organonitrogen-compound metabolic process	−17
	GO:0006807	Nitrogen compound metabolic process	−12
	GO:0043170	Macromolecule metabolic process	−12
	**Molecular Function (Gene Ontology)**		
	GO:0008233	Peptidase activity	−42
	GO:0008238	Exopeptidase activity	−35
	GO:0140096	Catalytic activity, acting on a protein	−25
	GO:0004177	Aminopeptidase activity	−24
	GO:0008237	Metallopeptidase activity	−24
	**Cellular Component (Gene Ontology)**		
	GO:0005615	Extracellular space	−11
	GO:0005576	Extracellular region	−11
	GO:0031982	Vesicle	−7
	GO:0070062	Extracellular exosome	−7
	GO:0030141	Secretory granule	−6
	**Local Network Cluster (STRING)**		
	CL:31766	Aminopeptidase and Meprin A complex	
	CL:31763	Mixed, incl. Aminopeptidase and Dipeptidase activity	−23
	CL:31768	Mixed, incl. Dipeptidyl-peptidase activity, and Creatinase/Aminopeptidase P/Spt16, N-terminal	−21
	CL:31769	Dipeptidyl-peptidase activity and Meprin A complex	−12
	CL:31770	Dipeptidyl peptidase IV (DPP IV) N-terminal region, and Pleurisy	−9
	**KEGG Pathways**		
	hsa04614	Renin-angiotensin system	−21
	hsa04974	Protein digestion and absorption	−5
	hsa04924	Renin secretion	−4
	hsa00330	Arginine and proline metabolism	−3
	hsa04080	Neuroactive ligand-receptor interaction	−3
**Lactadherin**	**Biological Process (Gene Ontology)**		
	GO:0002376	Immune system process	−21
	GO:0043277	Apoptotic cell clearance	−18
	GO:0002682	Regulation of immune system process	−17
	GO:0051239	Regulation of multicellular organismal process	−17
	GO:0001817	Regulation of cytokine production	−16
	**Molecular Function (Gene Ontology)**		
	GO:0005102	Signaling receptor binding	−23
	GO:0044877	Protein-containing complex binding	−11
	GO:0005515	Protein binding	−10
	GO:0001618	Virus receptor activity	−10
	GO:0005178	Integrin binding	−10
	**Cellular Component (Gene Ontology)**		
	GO:0005615	Extracellular space	−30
	GO:0005576	Extracellular region	−28
	GO:0009986	Cell surface	−26
	GO:0009897	External side of plasma membrane	−22
	GO:0071944	Cell periphery	−21
	**Local Network Cluster (STRING)**		
	CL:31956	Mixed, incl. Butyrophilin (BTN) family interactions and milk protein	−20
	CL:31958	Butyrophilin (BTN) family interactions, and milk protein	−16
	CL:19587	Protein complex involved in cell adhesion and junctional epidermolysis bullosa	−14
	CL:31959	Butyrophilin (BTN) family interactions and blepharitis	−14
	CL:19588	Integrin domain superfamily and cerebral malaria	−14
	**KEGG Pathways**		
	hsa04512	ECM-receptor interaction	−10
	hsa04510	Focal adhesion	−7
	hsa05144	Malaria	−7
	hsa04810	Regulation of actin cytoskeleton	−6
	hsa04145	Phagosome	−5
**Serum amyloid P-component**	**Biological Process (Gene Ontology)**		
	GO:0006952	Defense response	−35
	GO:0006959	Humoral immune response	−29
	GO:0006950	Response to stress	−27
	GO:0006953	Acute-phase response	−25
	GO:0002526	Acute inflammatory response	−23
	**Molecular Function (Gene Ontology)**		
	GO:0030527	Structural constituent of chromatin	−30
	GO:0046982	Protein heterodimerization activity	−22
	GO:0004866	Endopeptidase inhibitor activity	−19
	GO:0061134	Peptidase regulator activity	−18
	GO:0005198	Structural molecule activity	−18
	**Cellular Component (Gene Ontology)**		
	GO:0005615	Extracellular space	−79
	GO:0005576	Extracellular region	−72
	GO:0072562	Blood microparticle	−63
	GO:0070062	Extracellular exosome	−61
	GO:0031982	Vesicle	−46
	**Local Network Cluster (STRING)**		
	CL:18726	Complement and coagulation cascades and protein-lipid complex	−78
	CL:18723	Mixed, incl. Complement and coagulation cascades and protein-lipid complex	−76
	CL:18727	Complement and coagulation cascades and positive regulation of opsonization	−58
	CL:18728	Complement and coagulation cascades and positive regulation of opsonization	−56
	CL:18730	Hemostasis and Dissolution of Fibrin Clot	−29
	**KEGG Pathways**		
	hsa05322	Systemic lupus erythematosus	−46
	hsa04610	Complement and coagulation cascades	−35
	hsa05034	Alcoholism	−26
	hsa05150	Staphylococcus aureus infection	−13
	hsa05203	Viral carcinogenesis	−13
**Cholinesterase**	**Biological Process (Gene Ontology)**		
	GO:0065008	Regulation of biological quality	−15
	GO:0001505	Regulation of neurotransmitter levels	−12
	GO:0007268	Chemical synaptic transmission	−12
	GO:0099536	Synaptic signaling	−12
	GO:0009636	Response to toxic substance	−11
	**Molecular Function (Gene Ontology)**		
	GO:0016209	Antioxidant activity	−10
	GO:0004601	Peroxidase activity	−6
	GO:0016491	Oxidoreductase activity	−6
	GO:0005102	Signaling receptor binding	−6
	GO:0004602	Glutathione peroxidase activity	−6
	**Cellular Component (Gene Ontology)**		
	GO:0005788	Endoplasmic reticulum lumen	−12
	GO:0045202	Synapse	−12
	GO:0031983	Vesicle lumen	−11
	GO:0005576	Extracellular region	−11
	GO:0060205	Cytoplasmic vesicle lumen	−11
	**Local Network Cluster (STRING)**		
	CL:23578	Mixed, incl. Synaptic transmission, cholinergic, and monoamine GPCRs	−18
	CL:23678	Acetylcholine-gated channel complex and myasthenia gravis	−12
	CL:23710	Congenital myasthenic syndrome	−8
	CL:23680	Acetylcholine-gated channel complex and myasthenia gravis	−8
	CL:9665	Glutathione peroxidase, and glutathione-disulfide reductase (NADPH) activity	−7
	**KEGG Pathways**		
	hsa05010	Alzheimer disease	−9
	hsa01100	Metabolic pathways	−9
	hsa04918	Thyroid hormone synthesis	−6
	hsa00480	Glutathione metabolism	−6
	hsa00590	Arachidonic acid metabolism	−5
**NADH-ubiquinone oxidoreductase chain 4**	**Biological Process (Gene Ontology)**		
	GO:0006119	Oxidative phosphorylation	−102
	GO:0009060	Aerobic respiration	−99
	GO:0045333	Cellular respiration	−98
	GO:0042775	Mitochondrial ATP synthesis-coupled electron transport	−94
	GO:0022904	Respiratory electron transport chain	−92
	**Molecular Function (Gene Ontology)**		
	GO:0009055	Electron transfer activity	−74
	GO:0015453	Oxidoreduction-driven active transmembrane transporter activity	−71
	GO:0015399	Primary active transmembrane transporter activity	−58
	GO:0003955	NAD(P)H dehydrogenase (quinone) activity	−50
	GO:0008137	NADH dehydrogenase (ubiquinone) activity	−50
	**Cellular Component (Gene Ontology)**		
	GO:0005743	Mitochondrial inner membrane	−108
	GO:0019866	Organelle inner membrane	−104
	GO:0098800	Inner mitochondrial membrane protein complex	−104
	GO:0070469	Respirasome	−102
	GO:0098798	Mitochondrial protein-containing complex	−101
	**Local Network Cluster (STRING)**		
	CL:11061	Respiratory electron transport, ATP synthesis by chemiosmotic coupling, heat production by uncoupling proteins, and cytochrome complex	−128
	CL:11066	Respiratory electron transport, ATP synthesis by chemiosmotic coupling, heat production by uncoupling proteins, and respiratory chain complex IV	−123
	CL:11068	Respiratory electron transport, ATP synthesis by chemiosmotic coupling, heat production by uncoupling proteins, proton-transporting ATP synthase complex	−123
	CL:11069	Respiratory electron transport, ATP synthesis by chemiosmotic coupling, and heat production by uncoupling proteins	−123
	CL:11070	Respiratory chain complex and Complex I biogenesis	−115
	**KEGG Pathways**		
	hsa00190	Oxidative phosphorylation	−105
	hsa04714	Thermogenesis	−100
	hsa05012	Parkinson disease	−90
	hsa05020	Prion disease	−83
	hsa05016	Huntington disease	−79

### Appendix B.2. Lactadherin (UniProt ID: Q08431)

Lactadherin is a 387-residue-long protein known by several other names, such as breast epithelial antigen BA46, human milk fat globule (HMFG), milk fat globule-epidermal growth factor 8 (MFG-E8), and a sperm-associated protein SED1. Based on this variety of names, one can conclude that lactadherin is a multifunctional protein. In light of this hypothesis, lactadherin, a secreted glycoprotein associated with the milk fat globule membrane (MFGM), was shown to be involved in the regulation of many biological and physiological processes, such as angiogenesis, atherosclerosis, hemostasis, phagocytosis, and tissue remodeling [400]. This protein is known to be overexpressed in breast tumors [401], as well as in melanoma, ovarian, colorectal, and other types of cancer [402]. Importantly, this protein is found in extracellular vesicles derived from cancer cell lines and cancer patients, and its presence there was associated with cancer aggressiveness and a worse prognosis [402]. Lactadherin is expressed in phagocytes and contributes to the removal of apoptotic cells [403]. Furthermore, it was reported that in the brains of patients with Alzheimer’s disease, there is a noticeable reduction in the lactadherin mRNA expression levels, suggesting that altered production or function of lactadherin may contribute to the initiation and/or progression of AD [403]. Although lactadherin can be isolated from the MFGM found in the milk of healthy donors, this protein is also expressed on the surface of acrosome-intact human sperm and in the anterior caput of the human epididymis [404].

Lactadherin is a mostly ordered protein (see Figure A13A,D) consisting of a signal peptide (residues 1–23, which is predicted to be a mostly disordered segment), an N-terminal EGF-like domain that includes residues 24–67, followed by two repeated C-domains (residues 70–225 and 230–387) of the F5/8 type C that mediate high-affinity binding to phosphatidylserine-containing membranes. Because of its highly ordered nature, human lactadherin is characterized by a low LLPS probability and does not contain DPRs (Figure A13B), indicating that this protein does not have obvious roles in the biogenesis of membrane-less organelles and biomolecular condensates. The STRING-generated PPI network centered at lactadherin includes 130 proteins engaged in 1468 interactions (see Figure A13C) and is characterized by an average node degree of 22.6 and an average local clustering coefficient of 0.664. The most statistically significant biological processes, molecular functions, and cellular components ascribed to the members of this network are listed in Table A1.

### Appendix B.3. Serum Amyloid P-Component (SAP, UniProt ID: P02743)

Serum amyloid P-component (SAP) is a 223-residue-long protein that is known to bind to all forms of amyloid fibrils [405] and is recognized as a universal constituent of human amyloid deposits, including the cerebral amyloids found in AD [406]. SAP is biosynthesized in hepatocytes and secreted into the blood [407,408]. This protein also acts as one of the acute phase proteins induced by various insults [409]. In fact, serum levels of SAP can increase up to 1000-fold in response to infection, neoplasia, rheumatoid arthritis, and trauma [409]. SAP is involved in the regulation of various aspects of the innate immune response, being able to inhibit fibrocyte differentiation and neutrophil adhesion to extracellular matrix proteins as well as promote the formation of immuno-regulatory macrophages [410].

Human SAP contains a signal peptide (residues 1–23, which is predicted to be a mostly disordered segment; see Figure A14A) and a pentraxin (PTV) domain (residues 24–223). Although this protein cannot phase separate (Figure A14B), it is involved in a multitude of protein–protein interactions, acting as the center of a dense PPI network containing 162 proteins connected by 3272 interactions. This network is characterized by an average node degree of 40.4 and an average local clustering coefficient of 0.719. The most statistically significant biological processes, molecular functions, cellular components, local network clusters (STRING), and KEGG pathways ascribed to the members of this network are listed in Table A1. SAP, being one of the members of a multifunctional and highly conserved protein superfamily of pentraxins, exists as a pentamer, where mostly β-structured monomers are assembled in a flat disk with a hole in the middle (see Figure A14D; PDB ID: 4AVS [411]).

### Appendix B.4. Cholinesterase (UniProt ID: P06276)

Being one of the most efficient enzymes, cholinesterases (butyrylcholinesterase, BChE, and its “sister” enzyme acetylcholinesterase, AChE) can hydrolyze compounds that contain ester, amide, and thioester bonds, including toxic esters such as cocaine or scavenge organophosphorus pesticides and nerve agents, and thereby play important pharmacological and toxicological roles [412,413]. Furthermore, cholinesterases serve as an illustration of moonlighting proteins, being involved in roles that are independent of their catalytic activities, such as cell differentiation and neuronal and muscular development [414,415,416]. Furthermore, these proteins are associated with the pathogenesis of several diseases, such as AD, cardiovascular and cerebrovascular diseases, hepatic disorders, inflammatory conditions, insulin resistance, and type 2 diabetes mellitus [417]. In fact, compounds that inhibit the AChE and BChE enzymes are prescribed to prevent the progression of AD [418].

Human BChE is a highly ordered protein (see Figure A15A) with low LLPS potential and one DPR (see Figure A15B). This protein emerges at the center of a dense PPI network containing 119 proteins involved in 1329 interactions. This network is characterized by an average node degree of 22.3 and an average local clustering coefficient of 0.708. The most statistically significant biological processes, molecular functions, cellular components, local network clusters (STRING), and KEGG pathways ascribed to the members of this network are listed in Table A1. BChE is a 340 kDa disulfide-linked homo-tetrameric glycoprotein [419]. Figure A15D represents the X-ray crystal structure of human BChE solved at a resolution of 2.00 Å (PDB ID: 1P0I [420]).

### Appendix B.5. NADH-Ubiquinone Oxidoreductase Chain 4 (MT-ND4, UniProt ID: P03905)

NADH-ubiquinone oxidoreductase chain 4 (MT-ND4), also known as NADH dehydrogenase subunit 4, is a 459-residue-long core subunit of Complex I, i.e., the mitochondrial membrane respiratory chain NADH dehydrogenase, that uses ubiquinone as an electron acceptor while catalyzing electron transfer from NADH through the respiratory chain [421]. It was shown that the development of neurodegenerative diseases is frequently associated with Complex I dysfunction [422,423,424,425,426,427]. For example, degeneration of the dopaminergic *substantia nigra pars compacta* in idiopathic Parkinson’s disease (iPD) is characterized by the presence of α-synuclein-based Lewy pathology [32] and mitochondrial respiratory complex I deficiency [428]. Recent analysis of samples from individuals with PD revealed that early forms of α-synuclein aggregates (and not Lewy bodies, LBs) are associated with neuronal Complex I deficiency [429].

Figure A16A,D show that human NADH-ubiquinone oxidoreductase chain 4 is a highly ordered protein that contains several short IDRs. Furthermore, this protein is characterized by a low p_LLPS_ value of 0.1239, indicating that MT-ND4 is not capable of spontaneous LLPS. Since it also does not contain DPRs, it seems that this protein is not related to the biogenesis of membrane-less organelles and biomolecular condensates (see Figure A16B). Figure A16C shows that MT-ND4 forms a very dense PPI network containing 305 nodes linked by 9377 edges. With an average node degree of 61.5 and an average local clustering coefficient of 0.759, this network is one of the most connected among the networks considered in this study. Table A1 lists the most statistically significant biological processes, molecular functions, cellular components, local network clusters (STRING), and KEGG pathways ascribed to the members of this network.

## References

[1] Uversky V.N. (2017). Looking at the recent advances in understanding alpha-synuclein and its aggregation through the proteoform prism. F1000Research.

[2] Stefanis L. (2012). α-Synuclein in Parkinson’s disease. Cold Spring Harb. Perspect. Med..

[3] Weinreb P.H., Zhen W., Poon A.W., Conway K.A., Lansbury P.T. (1996). NACP, a protein implicated in Alzheimer’s disease and learning, is natively unfolded. Biochemistry.

[4] Uversky V.N., Li J., Fink A.L. (2001). Evidence for a partially folded intermediate in alpha-synuclein fibril formation. J. Biol. Chem..

[5] Eliezer D., Kutluay E., Bussell R., Browne G. (2001). Conformational properties of α-synuclein in its free and lipid-associated states. J. Mol. Biol..

[6] Uversky V.N., Li J., Souillac P., Millett I.S., Doniach S., Jakes R., Goedert M., Fink A.L. (2002). Biophysical properties of the synucleins and their propensities to fibrillate: Inhibition of α-synuclein assembly by β- and γ-synucleins. J. Biol. Chem..

[7] Uversky V.N. (2003). A protein-chameleon: Conformational plasticity of α-synuclein, a disordered protein involved in neurodegenerative disorders. J. Biomol. Struct. Dyn..

[8] Sung Y.H., Eliezer D. (2006). Secondary structure and dynamics of micelle bound β- and γ-synuclein. Protein Sci..

[9] Sung Y.H., Eliezer D. (2007). Residual structure, backbone dynamics, and interactions within the synuclein family. J. Mol. Biol..

[10] Binolfi A., Theillet F.X., Selenko P. (2012). Bacterial in-cell NMR of human α-synuclein: A disordered monomer by nature?. Biochem. Soc. Trans..

[11] Limatola A., Eichmann C., Jacob R.S., Ben-Nissan G., Sharon M., Binolfi A., Selenko P. (2018). Time-Resolved NMR Analysis of Proteolytic α-Synuclein Processing in vitro and in cellulo. Proteomics.

[12] Lopez J., Schneider R., Cantrelle F.X., Huvent I., Lippens G. (2016). Studying Intrinsically Disordered Proteins under True In Vivo Conditions by Combined Cross-Polarization and Carbonyl-Detection NMR Spectroscopy. Angew. Chem. Int. Ed. Engl..

[13] Sciolino N., Burz D.S., Shekhtman A. (2019). In-Cell NMR Spectroscopy of Intrinsically Disordered Proteins. Proteomics.

[14] Smith A.E., Zhou L.Z., Pielak G.J. (2015). Hydrogen exchange of disordered proteins in *Escherichia coli*. Protein Sci..

[15] Theillet F.X., Binolfi A., Bekei B., Martorana A., Rose H.M., Stuiver M., Verzini S., Lorenz D., van Rossum M., Goldfarb D. (2016). Structural disorder of monomeric α-synuclein persists in mammalian cells. Nature.

[16] Waudby C.A., Camilloni C., Fitzpatrick A.W., Cabrita L.D., Dobson C.M., Vendruscolo M., Christodoulou J. (2013). In-cell NMR characterization of the secondary structure populations of a disordered conformation of α-synuclein within *E. coli* cells. PLoS ONE.

[17] Galvin J.E., Lee V.M., Trojanowski J.Q. (2001). Synucleinopathies: Clinical and pathological implications. Arch. Neurol..

[18] Goedert M. (1999). Filamentous nerve cell inclusions in neurodegenerative diseases: Tauopathies and α-synucleinopathies. Philos. Trans. R. Soc. Lond. B Biol. Sci..

[19] Goedert M. (2001). α-synuclein and neurodegenerative diseases. Nat. Rev. Neurosci..

[20] Goedert M. (2001). Parkinson’s disease and other α-synucleinopathies. Clin. Chem. Lab. Med..

[21] Goedert M., Falcon B., Clavaguera F., Tolnay M. (2014). Prion-like mechanisms in the pathogenesis of tauopathies and synucleinopathies. Curr. Neurol. Neurosci. Rep..

[22] Goedert M., Jakes R., Spillantini M.G. (2017). The Synucleinopathies: Twenty Years On. J. Park. Dis..

[23] Spillantini M.G., Goedert M. (2000). The α-synucleinopathies: Parkinson’s disease, dementia with Lewy bodies, and multiple system atrophy. Ann. N. Y. Acad. Sci..

[24] McKeith I.G., Dickson D.W., Lowe J., Emre M., O’Brien J.T., Feldman H., Cummings J., Duda J.E., Lippa C., Perry E.K. (2005). Diagnosis and management of dementia with Lewy bodies: Third report of the DLB Consortium. Neurology.

[25] Wakabayashi K., Yoshimoto M., Tsuji S., Takahashi H. (1998). α-synuclein immunoreactivity in glial cytoplasmic inclusions in multiple system atrophy. Neurosci. Lett..

[26] Spillantini M.G., Crowther R.A., Jakes R., Cairns N.J., Lantos P.L., Goedert M. (1998). Filamentous α-synuclein inclusions link multiple system atrophy with Parkinson’s disease and dementia with Lewy bodies. Neurosci. Lett..

[27] Gai W.P., Power J.H., Blumbergs P.C., Blessing W.W. (1998). Multiple-system atrophy: A new α-synuclein disease?. Lancet.

[28] Trojanowski J.Q., Goedert M., Iwatsubo T., Lee V.M. (1998). Fatal attractions: Abnormal protein aggregation and neuron death in Parkinson’s disease and Lewy body dementia. Cell Death Differ..

[29] Takeda A., Mallory M., Sundsmo M., Honer W., Hansen L., Masliah E. (1998). Abnormal accumulation of NACP/α-synuclein in neurodegenerative disorders. Am. J. Pathol..

[30] Lucking C.B., Brice A. (2000). α-synuclein and Parkinson’s disease. Cell Mol. Life Sci..

[31] Arawaka S., Saito Y., Murayama S., Mori H. (1998). Lewy body in neurodegeneration with brain iron accumulation type 1 is immunoreactive for α-synuclein. Neurology.

[32] Spillantini M.G., Schmidt M.L., Lee V.M., Trojanowski J.Q., Jakes R., Goedert M. (1997). α-synuclein in Lewy bodies. Nature.

[33] Wakabayashi K., Matsumoto K., Takayama K., Yoshimoto M., Takahashi H. (1997). NACP, a presynaptic protein, immunoreactivity in Lewy bodies in Parkinson’s disease. Neurosci. Lett..

[34] Burre J., Sharma M., Sudhof T.C. (2018). Cell Biology and Pathophysiology of α-Synuclein. Cold Spring Harb. Perspect. Med..

[35] Goedert M., Spillantini M.G. (2012). Synucleinopathies and tauopathies. Basic Neurochemistry.

[36] Surguchov A., Surguchev A. (2022). Synucleins: New Data on Misfolding, Aggregation and Role in Diseases. Biomedicines.

[37] Spillantini M.G., Crowther R.A., Jakes R., Hasegawa M., Goedert M. (1998). α-Synuclein in filamentous inclusions of Lewy bodies from Parkinson’s disease and dementia with lewy bodies. Proc. Natl. Acad. Sci. USA.

[38] Trojanowski J.Q., Lee V.M. (2003). Parkinson’s disease and related α-synucleinopathies are brain amyloidoses. Ann. N. Y. Acad. Sci..

[39] Lundvig D., Lindersson E., Jensen P.H. (2005). Pathogenic effects of alpha-synuclein aggregation. Brain Res. Mol. Brain Res..

[40] Kosaka K. (1978). Lewy bodies in cerebral cortex, report of three cases. Acta Neuropathol..

[41] Kosaka K., Mehraein P. (1979). Dementia-Parkinsonism syndrome with numerous Lewy bodies and senile plaques in cerebral cortex. Arch. Psychiatr. Nervenkr.

[42] Seidel K., Mahlke J., Siswanto S., Kruger R., Heinsen H., Auburger G., Bouzrou M., Grinberg L.T., Wicht H., Korf H.W. (2015). The brainstem pathologies of Parkinson’s disease and dementia with Lewy bodies. Brain Pathol..

[43] Lerner A., Bagic A. (2008). Olfactory pathogenesis of idiopathic Parkinson disease revisited. Mov. Disord..

[44] Visanji N.P., Brooks P.L., Hazrati L.N., Lang A.E. (2013). The prion hypothesis in Parkinson’s disease: Braak to the future. Acta Neuropathol. Commun..

[45] Melki R. (2015). Role of Different Alpha-Synuclein Strains in Synucleinopathies, Similarities with other Neurodegenerative Diseases. J. Park. Dis..

[46] Peelaerts W., Bousset L., Van der Perren A., Moskalyuk A., Pulizzi R., Giugliano M., Van den Haute C., Melki R., Baekelandt V. (2015). α-Synuclein strains cause distinct synucleinopathies after local and systemic administration. Nature.

[47] Tofaris G.K., Spillantini M.G. (2007). Physiological and pathological properties of α-synuclein. Cell Mol. Life Sci..

[48] Li J., Uversky V.N., Fink A.L. (2001). Effect of familial Parkinson’s disease point mutations A30P and A53T on the structural properties, aggregation, and fibrillation of human α-synuclein. Biochemistry.

[49] Conway K.A., Harper J.D., Lansbury P.T. (1998). Accelerated in vitro fibril formation by a mutant α-synuclein linked to early-onset Parkinson disease. Nat. Med..

[50] Conway K.A., Harper J.D., Lansbury P.T. (2000). Fibrils formed in vitro from alpha-synuclein and two mutant forms linked to Parkinson’s disease are typical amyloid. Biochemistry.

[51] Conway K.A., Lee S.J., Rochet J.C., Ding T.T., Williamson R.E., Lansbury P.T. (2000). Acceleration of oligomerization, not fibrillization, is a shared property of both alpha-synuclein mutations linked to early-onset Parkinson’s disease: Implications for pathogenesis and therapy. Proc. Natl. Acad. Sci. USA.

[52] Lashuel H.A., Hartley D., Petre B.M., Walz T., Lansbury P.T. (2002). Neurodegenerative disease: Amyloid pores from pathogenic mutations. Nature.

[53] Lashuel H.A., Petre B.M., Wall J., Simon M., Nowak R.J., Walz T., Lansbury P.T. (2002). α-synuclein, especially the Parkinson’s disease-associated mutants, forms pore-like annular and tubular protofibrils. J. Mol. Biol..

[54] Proukakis C., Dudzik C.G., Brier T., MacKay D.S., Cooper J.M., Millhauser G.L., Houlden H., Schapira A.H. (2013). A novel α-synuclein missense mutation in Parkinson disease. Neurology.

[55] Appel-Cresswell S., Vilarino-Guell C., Encarnacion M., Sherman H., Yu I., Shah B., Weir D., Thompson C., Szu-Tu C., Trinh J. (2013). α-synuclein p.H50Q, a novel pathogenic mutation for Parkinson’s disease. Mov. Disord..

[56] Khalaf O., Fauvet B., Oueslati A., Dikiy I., Mahul-Mellier A.L., Ruggeri F.S., Mbefo M.K., Vercruysse F., Dietler G., Lee S.J. (2014). The H50Q mutation enhances α-synuclein aggregation, secretion, and toxicity. J. Biol. Chem..

[57] Dev K.K., Hofele K., Barbieri S., Buchman V.L., van der Putten H. (2003). Part II: α-synuclein and its molecular pathophysiological role in neurodegenerative disease. Neuropharmacology.

[58] da Costa C.A., Ancolio K., Checler F. (2000). Wild-type but not Parkinson’s disease-related ala-53 → Thr mutant α-synuclein protects neuronal cells from apoptotic stimuli. J. Biol. Chem..

[59] Uversky V.N., Li J., Fink A.L. (2001). Metal-triggered structural transformations, aggregation, and fibrillation of human α-synuclein. A possible molecular NK between Parkinson’s disease and heavy metal exposure. J. Biol. Chem..

[60] Santner A., Uversky V.N. (2010). Metalloproteomics and metal toxicology of α-synuclein. Metallomics.

[61] Ahmad A., Burns C.S., Fink A.L., Uversky V.N. (2012). Peculiarities of copper binding to α-synuclein. J. Biomol. Struct. Dyn..

[62] Carboni E., Lingor P. (2015). Insights on the interaction of α-synuclein and metals in the pathophysiology of Parkinson’s disease. Metallomics.

[63] Uversky V.N., Li J., Bower K., Fink A.L. (2002). Synergistic effects of pesticides and metals on the fibrillation of α-synuclein: Implications for Parkinson’s disease. Neurotoxicology.

[64] Uversky V.N., Li J., Fink A.L. (2001). Pesticides directly accelerate the rate of α-synuclein fibril formation: A possible factor in Parkinson’s disease. FEBS Lett..

[65] Maturana M.G., Pinheiro A.S., de Souza T.L., Follmer C. (2015). Unveiling the role of the pesticides paraquat and rotenone on α-synuclein fibrillation in vitro. Neurotoxicology.

[66] Ottolini D., Cali T., Szabo I., Brini M. (2017). α-synuclein at the intracellular and the extracellular side: Functional and dysfunctional implications. Biol. Chem..

[67] Emanuele M., Chieregatti E. (2015). Mechanisms of α-synuclein action on neurotransmission: Cell-autonomous and non-cell autonomous role. Biomolecules.

[68] Uversky V.N. (2008). α-synuclein misfolding and neurodegenerative diseases. Curr. Protein Pept. Sci..

[69] Payton J.E., Perrin R.J., Clayton D.F., George J.M. (2001). Protein-protein interactions of α-synuclein in brain homogenates and transfected cells. Brain Res. Mol. Brain Res..

[70] Jin J., Li G.J., Davis J., Zhu D., Wang Y., Pan C., Zhang J. (2007). Identification of novel proteins associated with both alpha-synuclein and DJ-1. Mol. Cell Proteom..

[71] Iwai A., Masliah E., Yoshimoto M., Ge N., Flanagan L., de Silva H.A., Kittel A., Saitoh T. (1995). The precursor protein of non-Aβ component of Alzheimer’s disease amyloid is a presynaptic protein of the central nervous system. Neuron.

[72] Hayashi J., Carver J.A. (2022). beta-Synuclein: An Enigmatic Protein with Diverse Functionality. Biomolecules.

[73] Ji H., Liu Y.E., Jia T., Wang M., Liu J., Xiao G., Joseph B.K., Rosen C., Shi Y.E. (1997). Identification of a breast cancer-specific gene, BCSG1, by direct differential cDNA sequencing. Cancer Res..

[74] Dunker A.K., Obradovic Z., Romero P., Garner E.C., Brown C.J. (2000). Intrinsic protein disorder in complete genomes. Genome Inform. Ser. Workshop Genome Inform..

[75] Uversky V.N. (2010). The mysterious unfoldome: Structureless, underappreciated, yet vital part of any given proteome. J. Biomed. Biotechnol..

[76] Ward J.J., Sodhi J.S., McGuffin L.J., Buxton B.F., Jones D.T. (2004). Prediction and functional analysis of native disorder in proteins from the three kingdoms of life. J. Mol. Biol..

[77] Uversky V.N., Gillespie J.R., Fink A.L. (2000). Why are “natively unfolded” proteins unstructured under physiologic conditions?. Proteins.

[78] Xue B., Dunker A.K., Uversky V.N. Orderly order in protein intrinsic disorder distribution: Disorder in thirty five hundred proteomes from viruses and the three domains of life. J. Biomol. Struct. Dyn..

[79] Dunker A.K., Garner E., Guilliot S., Romero P., Albrecht K., Hart J., Obradovic Z., Kissinger C., Villafranca J.E. (1998). Protein disorder and the evolution of molecular recognition: Theory, predictions and observations. Pac. Symp. Biocomput..

[80] Wright P.E., Dyson H.J. (1999). Intrinsically unstructured proteins: Re-assessing the protein structure-function paradigm. J. Mol. Biol..

[81] Dunker A.K., Lawson J.D., Brown C.J., Williams R.M., Romero P., Oh J.S., Oldfield C.J., Campen A.M., Ratliff C.M., Hipps K.W. (2001). Intrinsically disordered protein. J. Mol. Graph. Model..

[82] Tompa P. (2002). Intrinsically unstructured proteins. Trends Biochem. Sci..

[83] Daughdrill G.W., Pielak G.J., Uversky V.N., Cortese M.S., Dunker A.K., Buchner J., Kiefhaber T. (2005). Natively disordered proteins. Handbook of Protein Folding.

[84] Uversky V.N., Dunker A.K. (2010). Understanding protein non-folding. Biochim. Biophys. Acta.

[85] Dunker A.K., Obradovic Z. (2001). The protein trinity-linking function and disorder. Nat. Biotechnol..

[86] Uversky V.N. (2002). Natively unfolded proteins: A point where biology waits for physics. Protein Sci..

[87] Uversky V.N. (2013). Unusual biophysics of intrinsically disordered proteins. Biochim. Biophys. Acta.

[88] Uversky V.N. (2013). Intrinsic disorder-based protein interactions and their modulators. Curr. Pharm. Des..

[89] Uversky V.N. (2015). Functional roles of transiently and intrinsically disordered regions within proteins. FEBS J..

[90] Uversky V.N. (2016). p53 Proteoforms and Intrinsic Disorder: An Illustration of the Protein Structure-Function Continuum Concept. Int. J. Mol. Sci..

[91] Uversky V.N. (2019). Protein intrinsic disorder and structure-function continuum. Prog. Mol. Biol. Transl. Sci..

[92] Fonin A.V., Darling A.L., Kuznetsova I.M., Turoverov K.K., Uversky V.N. (2019). Multi-functionality of proteins involved in GPCR and G protein signaling: Making sense of structure-function continuum with intrinsic disorder-based proteoforms. Cell Mol. Life Sci..

[93] Gupta M.N., Uversky V.N. (2024). Protein structure-function continuum model: Emerging nexuses between specificity, evolution, and structure. Protein Sci..

[94] Iakoucheva L.M., Brown C.J., Lawson J.D., Obradovic Z., Dunker A.K. (2002). Intrinsic disorder in cell-signaling and cancer-associated proteins. J. Mol. Biol..

[95] Dunker A.K., Cortese M.S., Romero P., Iakoucheva L.M., Uversky V.N. (2005). Flexible nets: The roles of intrinsic disorder in protein interaction networks. FEBS J..

[96] Uversky V.N., Oldfield C.J., Dunker A.K. (2005). Showing your ID: Intrinsic disorder as an ID for recognition, regulation and cell signaling. J. Mol. Recognit..

[97] Radivojac P., Iakoucheva L.M., Oldfield C.J., Obradovic Z., Uversky V.N., Dunker A.K. (2007). Intrinsic disorder and functional proteomics. Biophys. J..

[98] Vucetic S., Xie H., Iakoucheva L.M., Oldfield C.J., Dunker A.K., Obradovic Z., Uversky V.N. (2007). Functional anthology of intrinsic disorder. 2. Cellular components, domains, technical terms, developmental processes, and coding sequence diversities correlated with long disordered regions. J. Proteome Res..

[99] Xie H., Vucetic S., Iakoucheva L.M., Oldfield C.J., Dunker A.K., Uversky V.N., Obradovic Z. (2007). Functional anthology of intrinsic disorder. 1. Biological processes and functions of proteins with long disordered regions. J. Proteome Res..

[100] Xie H., Vucetic S., Iakoucheva L.M., Oldfield C.J., Dunker A.K., Obradovic Z., Uversky V.N. (2007). Functional anthology of intrinsic disorder. 3. Ligands, post-translational modifications, and diseases associated with intrinsically disordered proteins. J. Proteome Res..

[101] Habchi J., Tompa P., Longhi S., Uversky V.N. (2014). Introducing protein intrinsic disorder. Chem. Rev..

[102] van der Lee R., Buljan M., Lang B., Weatheritt R.J., Daughdrill G.W., Dunker A.K., Fuxreiter M., Gough J., Gsponer J., Jones D.T. (2014). Classification of intrinsically disordered regions and proteins. Chem. Rev..

[103] Uversky V.N. (2011). Multitude of binding modes attainable by intrinsically disordered proteins: A portrait gallery of disorder-based complexes. Chem. Soc. Rev..

[104] Iakoucheva L.M., Radivojac P., Brown C.J., O’Connor T.R., Sikes J.G., Obradovic Z., Dunker A.K. (2004). The importance of intrinsic disorder for protein phosphorylation. Nucleic Acids Res..

[105] Pejaver V., Hsu W.L., Xin F., Dunker A.K., Uversky V.N., Radivojac P. (2014). The structural and functional signatures of proteins that undergo multiple events of post-translational modification. Protein Sci..

[106] Uversky V.N. (2017). Intrinsically disordered proteins in overcrowded milieu: Membrane-less organelles, phase separation, and intrinsic disorder. Curr. Opin. Struct. Biol..

[107] Uversky V.N. (2017). Protein intrinsic disorder-based liquid-liquid phase transitions in biological systems: Complex coacervates and membrane-less organelles. Adv. Colloid Interface Sci..

[108] Uversky V.N. (2021). Recent Developments in the Field of Intrinsically Disordered Proteins: Intrinsic Disorder–Based Emergence in Cellular Biology in Light of the Physiological and Pathological Liquid–Liquid Phase Transitions. Annu. Rev. Biophys..

[109] Mohan A., Sullivan W.J., Radivojac P., Dunker A.K., Uversky V.N. (2008). Intrinsic disorder in pathogenic and non-pathogenic microbes: Discovering and analyzing the unfoldomes of early-branching eukaryotes. Mol. Biosyst..

[110] Lee H., Mok K.H., Muhandiram R., Park K.H., Suk J.E., Kim D.H., Chang J., Sung Y.C., Choi K.Y., Han K.H. (2000). Local structural elements in the mostly unstructured transcriptional activation domain of human p53. J. Biol. Chem..

[111] Adkins J.N., Lumb K.J. (2002). Intrinsic structural disorder and sequence features of the cell cycle inhibitor p57Kip2. Proteins.

[112] Chang B.S., Minn A.J., Muchmore S.W., Fesik S.W., Thompson C.B. (1997). Identification of a novel regulatory domain in Bcl-X(L) and Bcl-2. EMBO J..

[113] Campbell K.M., Terrell A.R., Laybourn P.J., Lumb K.J. (2000). Intrinsic structural disorder of the C-terminal activation domain from the bZIP transcription factor Fos. Biochemistry.

[114] Sunde M., McGrath K.C., Young L., Matthews J.M., Chua E.L., Mackay J.P., Death A.K. (2004). TC-1 is a novel tumorigenic and natively disordered protein associated with thyroid cancer. Cancer Res..

[115] Glenner G.G., Wong C.W. (1984). Alzheimer’s disease and Down’s syndrome: Sharing of a unique cerebrovascular amyloid fibril protein. Biochem. Biophys. Res. Commun..

[116] Masters C.L., Multhaup G., Simms G., Pottgiesser J., Martins R.N., Beyreuther K. (1985). Neuronal origin of a cerebral amyloid: Neurofibrillary tangles of Alzheimer’s disease contain the same protein as the amyloid of plaque cores and blood vessels. EMBO J..

[117] Lee V.M., Balin B.J., Otvos L., Trojanowski J.Q. (1991). A68: A major subunit of paired helical filaments and derivatized forms of normal Tau. Science.

[118] Ueda K., Fukushima H., Masliah E., Xia Y., Iwai A., Yoshimoto M., Otero D.A., Kondo J., Ihara Y., Saitoh T. (1993). Molecular cloning of cDNA encoding an unrecognized component of amyloid in Alzheimer disease. Proc. Natl. Acad. Sci. USA.

[119] Wisniewski K.E., Dalton A.J., McLachlan C., Wen G.Y., Wisniewski H.M. (1985). Alzheimer’s disease in Down’s syndrome: Clinicopathologic studies. Neurology.

[120] Prusiner S.B. (2001). Shattuck lecture—Neurodegenerative diseases and prions. N. Engl. J. Med..

[121] Zoghbi H.Y., Orr H.T. (1999). Polyglutamine diseases: Protein cleavage and aggregation. Curr. Opin. Neurobiol..

[122] Cheng Y., LeGall T., Oldfield C.J., Dunker A.K., Uversky V.N. (2006). Abundance of intrinsic disorder in protein associated with cardiovascular disease. Biochemistry.

[123] Uversky V.N. (2008). Amyloidogenesis of natively unfolded proteins. Curr. Alzheimer Res..

[124] Uversky V.N., Oldfield C.J., Midic U., Xie H., Xue B., Vucetic S., Iakoucheva L.M., Obradovic Z., Dunker A.K. (2009). Unfoldomics of human diseases: Linking protein intrinsic disorder with diseases. BMC Genom..

[125] Uversky V.N. (2009). Intrinsic disorder in proteins associated with neurodegenerative diseases. Front. Biosci..

[126] Midic U., Oldfield C.J., Dunker A.K., Obradovic Z., Uversky V.N. Protein disorder in the human diseasome: Unfoldomics of human genetic diseases. PLoS Comput. Biol..

[127] Tompa P., Fuxreiter M., Oldfield C.J., Simon I., Dunker A.K., Uversky V.N. (2009). Close encounters of the third kind: Disordered domains and the interactions of proteins. Bioessays.

[128] George J.M., Jin H., Woods W.S., Clayton D.F. (1995). Characterization of a novel protein regulated during the critical period for song learning in the zebra finch. Neuron.

[129] Tanner C.M. (2003). Is the cause of Parkinson’s disease environmental or hereditary? Evidence from twin studies. Adv. Neurol..

[130] Farrer M.J. (2006). Genetics of Parkinson disease: Paradigm shifts and future prospects. Nat. Rev. Genet..

[131] Olanow C.W., Tatton W.G. (1999). Etiology and pathogenesis of Parkinson’s disease. Annu. Rev. Neurosci..

[132] Moghal S., Rajput A.H., D’Arcy C., Rajput R. (1994). Prevalence of movement disorders in elderly community residents. Neuroepidemiology.

[133] Fahn S. (2003). Description of Parkinson’s disease as a clinical syndrome. Ann. N. Y. Acad. Sci..

[134] Mizuno Y., Hattori N., Kitada T., Matsumine H., Mori H., Shimura H., Kubo S., Kobayashi H., Asakawa S., Minoshima S. (2001). Familial Parkinson’s disease. α-synuclein and parkin. Adv. Neurol..

[135] Van Den Eeden S.K., Tanner C.M., Bernstein A.L., Fross R.D., Leimpeter A., Bloch D.A., Nelson L.M. (2003). Incidence of Parkinson’s disease: Variation by age, gender, and race/ethnicity. Am. J. Epidemiol..

[136] Forno L.S. (1996). Neuropathology of Parkinson’s disease. J. Neuropathol. Exp. Neurol..

[137] Zarranz J.J., Alegre J., Gomez-Esteban J.C., Lezcano E., Ros R., Ampuero I., Vidal L., Hoenicka J., Rodriguez O., Atares B. (2004). The new mutation, E46K, of α-synuclein causes Parkinson and Lewy body dementia. Ann. Neurol..

[138] Polymeropoulos M.H., Lavedan C., Leroy E., Ide S.E., Dehejia A., Dutra A., Pike B., Root H., Rubenstein J., Boyer R. (1997). Mutation in the α-synuclein gene identified in families with Parkinson’s disease. Science.

[139] Kruger R., Kuhn W., Muller T., Woitalla D., Graeber M., Kosel S., Przuntek H., Epplen J.T., Schols L., Riess O. (1998). Ala30Pro mutation in the gene encoding α-synuclein in Parkinson’s disease. Nat. Genet..

[140] Singleton A., Gwinn-Hardy K., Sharabi Y., Li S.T., Holmes C., Dendi R., Hardy J., Crawley A., Goldstein D.S. (2004). Association between cardiac denervation and parkinsonism caused by α-synuclein gene triplication. Brain.

[141] Singleton A.B., Farrer M., Johnson J., Singleton A., Hague S., Kachergus J., Hulihan M., Peuralinna T., Dutra A., Nussbaum R. (2003). α-Synuclein locus triplication causes Parkinson’s disease. Science.

[142] Farrer M., Kachergus J., Forno L., Lincoln S., Wang D.S., Hulihan M., Maraganore D., Gwinn-Hardy K., Wszolek Z., Dickson D. (2004). Comparison of kindreds with parkinsonism and α-synuclein genomic multiplications. Ann. Neurol..

[143] Morar A.S., Olteanu A., Young G.B., Pielak G.J. (2001). Solvent-induced collapse of α-synuclein and acid-denatured cytochrome c. Protein Sci..

[144] Bussell R., Eliezer D. (2001). Residual structure and dynamics in Parkinson’s disease-associated mutants of α-synuclein. J. Biol. Chem..

[145] Dedmon M.M., Lindorff-Larsen K., Christodoulou J., Vendruscolo M., Dobson C.M. (2005). Mapping long-range interactions in α-synuclein using spin-label NMR and ensemble molecular dynamics simulations. J. Am. Chem. Soc..

[146] Bertoncini C.W., Jung Y.S., Fernandez C.O., Hoyer W., Griesinger C., Jovin T.M., Zweckstetter M. (2005). Release of long-range tertiary interactions potentiates aggregation of natively unstructured α-synuclein. Proc. Natl. Acad. Sci. USA.

[147] Hardenberg M., Horvath A., Ambrus V., Fuxreiter M., Vendruscolo M. (2020). Widespread occurrence of the droplet state of proteins in the human proteome. Proc. Natl. Acad. Sci. USA.

[148] Vendruscolo M., Fuxreiter M. (2022). Sequence Determinants of the Aggregation of Proteins within Condensates Generated by Liquid-liquid Phase Separation. J. Mol. Biol..

[149] Hardenberg M.C., Sinnige T., Casford S., Dada S.T., Poudel C., Robinson E.A., Fuxreiter M., Kaminksi C.F., Kaminski Schierle G.S., Nollen E.A.A. (2021). Observation of an α-synuclein liquid droplet state and its maturation into Lewy body-like assemblies. J. Mol. Cell Biol..

[150] Huang S., Mo X., Wang J., Ye X., Yu H., Liu Y. (2022). α-Synuclein phase separation and amyloid aggregation are modulated by C-terminal truncations. FEBS Lett..

[151] Huang S., Xu B., Liu Y. (2022). Calcium promotes α-synuclein liquid-liquid phase separation to accelerate amyloid aggregation. Biochem. Biophys. Res. Commun..

[152] Ray S., Singh N., Kumar R., Patel K., Pandey S., Datta D., Mahato J., Panigrahi R., Navalkar A., Mehra S. (2020). α-Synuclein aggregation nucleates through liquid-liquid phase separation. Nat. Chem..

[153] Sawner A.S., Ray S., Yadav P., Mukherjee S., Panigrahi R., Poudyal M., Patel K., Ghosh D., Kummerant E., Kumar A. (2021). Modulating α-Synuclein Liquid-Liquid Phase Separation. Biochemistry.

[154] Vendruscolo M., Fuxreiter M. (2022). Protein condensation diseases: Therapeutic opportunities. Nat. Commun..

[155] Chandra S., Chen X., Rizo J., Jahn R., Sudhof T.C. (2003). A broken α-helix in folded α-Synuclein. J. Biol. Chem..

[156] Davidson W.S., Jonas A., Clayton D.F., George J.M. (1998). Stabilization of α-synuclein secondary structure upon binding to synthetic membranes. J. Biol. Chem..

[157] Zhu M., Fink A.L. (2003). Lipid binding inhibits α-synuclein fibril formation. J. Biol. Chem..

[158] Munishkina L.A., Phelan C., Uversky V.N., Fink A.L. (2003). Conformational behavior and aggregation of α-synuclein in organic solvents: Modeling the effects of membranes. Biochemistry.

[159] Rao J.N., Jao C.C., Hegde B.G., Langen R., Ulmer T.S. (2010). A combinatorial NMR and EPR approach for evaluating the structural ensemble of partially folded proteins. J. Am. Chem. Soc..

[160] Ulmer T.S., Bax A., Cole N.B., Nussbaum R.L. (2005). Structure and dynamics of micelle-bound human α-synuclein. J. Biol. Chem..

[161] Marsh J.A., Singh V.K., Jia Z., Forman-Kay J.D. (2006). Sensitivity of secondary structure propensities to sequence differences between α- and γ-synuclein: Implications for fibrillation. Protein Sci..

[162] Ogata H., Goto S., Sato K., Fujibuchi W., Bono H., Kanehisa M. (1999). KEGG: Kyoto Encyclopedia of Genes and Genomes. Nucleic Acids Res..

[163] Kanehisa M., Furumichi M., Tanabe M., Sato Y., Morishima K. (2017). KEGG: New perspectives on genomes, pathways, diseases and drugs. Nucleic Acids Res..

[164] El-Agnaf O.M., Jakes R., Curran M.D., Wallace A. (1998). Effects of the mutations Ala30 to Pro and Ala53 to Thr on the physical and morphological properties of alpha-synuclein protein implicated in Parkinson’s disease. FEBS Lett..

[165] Choi W., Zibaee S., Jakes R., Serpell L.C., Davletov B., Crowther R.A., Goedert M. (2004). Mutation E46K increases phospholipid binding and assembly into filaments of human alpha-synuclein. FEBS Lett..

[166] Kiely A.P., Asi Y.T., Kara E., Limousin P., Ling H., Lewis P., Proukakis C., Quinn N., Lees A.J., Hardy J. (2013). α-Synucleinopathy associated with G51D SNCA mutation: A link between Parkinson’s disease and multiple system atrophy?. Acta Neuropathol..

[167] Lesage S., Anheim M., Letournel F., Bousset L., Honore A., Rozas N., Pieri L., Madiona K., Durr A., Melki R. (2013). G51D α-synuclein mutation causes a novel parkinsonian-pyramidal syndrome. Ann. Neurol..

[168] Pasanen P., Myllykangas L., Siitonen M., Raunio A., Kaakkola S., Lyytinen J., Tienari P.J., Poyhonen M., Paetau A. (2014). Novel α-synuclein mutation A53E associated with atypical multiple system atrophy and Parkinson’s disease-type pathology. Neurobiol. Aging.

[169] Lemkau L.R., Comellas G., Kloepper K.D., Woods W.S., George J.M., Rienstra C.M. (2012). Mutant protein A30P α-synuclein adopts wild-type fibril structure, despite slower fibrillation kinetics. J. Biol. Chem..

[170] Fredenburg R.A., Rospigliosi C., Meray R.K., Kessler J.C., Lashuel H.A., Eliezer D., Lansbury P.T. (2007). The impact of the E46K mutation on the properties of α-synuclein in its monomeric and oligomeric states. Biochemistry.

[171] Pandey N., Schmidt R.E., Galvin J.E. (2006). The α-synuclein mutation E46K promotes aggregation in cultured cells. Exp. Neurol..

[172] Rutherford N.J., Moore B.D., Golde T.E., Giasson B.I. (2014). Divergent effects of the H50Q and G51D SNCA mutations on the aggregation of α-synuclein. J. Neurochem..

[173] Ghosh D., Sahay S., Ranjan P., Salot S., Mohite G.M., Singh P.K., Dwivedi S., Carvalho E., Banerjee R., Kumar A. (2014). The newly discovered Parkinson’s disease associated Finnish mutation (A53E) attenuates α-synuclein aggregation and membrane binding. Biochemistry.

[174] Rutherford N.J., Giasson B.I. (2015). The A53E α-synuclein pathological mutation demonstrates reduced aggregation propensity in vitro and in cell culture. Neurosci. Lett..

[175] Rajagopalan K., Mooney S.M., Parekh N., Getzenberg R.H., Kulkarni P. (2011). A majority of the cancer/testis antigens are intrinsically disordered proteins. J. Cell Biochem..

[176] Uversky V.N., Kragelund B.B., Skriver K. (2020). Analyzing IDPs in interactomes. Intrinsically Disordered Proteins.

[177] Madeira F., Pearce M., Tivey A.R.N., Basutkar P., Lee J., Edbali O., Madhusoodanan N., Kolesnikov A., Lopez R. (2022). Search and sequence analysis tools services from EMBL-EBI in 2022. Nucleic Acids Res..

[178] Yuan J., Zhao Y. (2013). Evolutionary aspects of the synuclein super-family and sub-families based on large-scale phylogenetic and group-discrimination analysis. Biochem. Biophys. Res. Commun..

[179] Bonaccorsi di Patti M.C., Angiulli E., Casini A., Vaccaro R., Cioni C., Toni M. (2022). Synuclein Analysis in Adult *Xenopus laevis*. Int. J. Mol. Sci..

[180] Yuan Z., Zhao X., Yan F., Zhao J., Liu H., Xiong S., Li J., Chen L., Wei Y. (2007). Beta-synuclein protein from *Xenopus laevis*: Overexpression in Escherichia coli of the GST-tagged protein and production of polyclonal antibodies. Biochemistry.

[181] Liu H.Y., Cao K., Zhao X.Y., Yuan Z. (2011). Cloning, subcellular localization and in situ detection of *Xenopus laevis* beta-synnclein gene. Sichuan Da Xue Xue Bao Yi Xue Ban..

[182] Wang C., Liu Y., Chan W.Y., Chan S.O., Grunz H., Zhao H. (2011). Characterization of three synuclein genes in *Xenopus laevis*. Dev. Dyn..

[183] Seleem A.A. (2019). Teratogenicity and neurotoxicity effects induced by methomyl insecticide on the developmental stages of Bufo arabicus. Neurotoxicol. Teratol..

[184] Tiunova A.A., Anokhin K.V., Saha A.R., Schmidt O., Hanger D.P., Anderton B.H., Davies A.M., Ninkina N.N., Buchman V.L. (2000). Chicken synucleins: Cloning and expression in the developing embryo. Mech. Dev..

[185] Hartman V.N., Miller M.A., Clayton D.F., Liu W.C., Kroodsma D.E., Brenowitz E.A. (2001). Testosterone regulates α-synuclein mRNA in the avian song system. Neuroreport.

[186] Li M., Zhou S., Wang X.S., Liu C., Li S. (2017). Effects of in vitro and in vivo avermectin exposure on α synuclein expression and proteasomal activity in pigeons. Ecotoxicol. Environ. Saf..

[187] Maroteaux L., Campanelli J.T., Scheller R.H. (1988). Synuclein: A neuron-specific protein localized to the nucleus and presynaptic nerve terminal. J. Neurosci..

[188] Yoshida H., Craxton M., Jakes R., Zibaee S., Tavare R., Fraser G., Serpell L.C., Davletov B., Crowther R.A., Goedert M. (2006). Synuclein proteins of the pufferfish *Fugu rubripes*: Sequences and functional characterization. Biochemistry.

[189] Toni M., Cioni C. (2015). Fish Synucleins: An Update. Mar. Drugs.

[190] Vaccaro R., Toni M., Casini A., Vivacqua G., Yu S., D’Este L., Cioni C. (2015). Localization of α-synuclein in teleost central nervous system: Immunohistochemical and Western blot evidence by 3D5 monoclonal antibody in the common carp, *Cyprinus carpio*. J. Comp. Neurol..

[191] Matsui H., Kenmochi N., Namikawa K. (2019). Age- and α-Synuclein-Dependent Degeneration of Dopamine and Noradrenaline Neurons in the Annual Killifish *Nothobranchius furzeri*. Cell Rep..

[192] Toni M., Cioni C., De Angelis F., di Patti M.C. (2016). Synuclein expression in the lizard Anolis carolinensis. J. Comp. Physiol. A Neuroethol. Sens. Neural Behav. Physiol..

[193] Sun Z., Gitler A.D. (2008). Discovery and characterization of three novel synuclein genes in zebrafish. Dev. Dyn..

[194] Sun X., Xue B., Jones W.T., Rikkerink E., Dunker A.K., Uversky V.N. (2011). A functionally required unfoldome from the plant kingdom: Intrinsically disordered N-terminal domains of GRAS proteins are involved in molecular recognition during plant development. Plant Mol. Biol..

[195] Xue B., Oldfield C.J., Van Y.Y., Dunker A.K., Uversky V.N. (2012). Protein intrinsic disorder and induced pluripotent stem cells. Mol. Biosyst..

[196] Huang F., Oldfield C., Meng J., Hsu W.L., Xue B., Uversky V.N., Romero P., Dunker A.K. (2012). Subclassifying disordered proteins by the CH-CDF plot method. Pac. Symp. Biocomput..

[197] UniProt_Consortium (2023). UniProt: The Universal Protein Knowledgebase in 2023. Nucleic Acids Res.

[198] Jumper J., Evans R., Pritzel A., Green T., Figurnov M., Ronneberger O., Tunyasuvunakool K., Bates R., Zidek A., Potapenko A. (2021). Highly accurate protein structure prediction with AFold. Nature.

[199] Dayhoff G.W., Uversky V.N. (2022). Rapid prediction and analysis of protein intrinsic disorder. Protein Sci..

[200] Oates M.E., Romero P., Ishida T., Ghalwash M., Mizianty M.J., Xue B., Dosztanyi Z., Uversky V.N., Obradovic Z., Kurgan L. (2013). D^2^P^2^: Database of disordered protein predictions. Nucleic Acids Res..

[201] Hatos A., Tosatto S.C.E., Vendruscolo M., Fuxreiter M. (2022). FuzDrop on AFold: Visualizing the sequence-dependent propensity of liquid-liquid phase separation and aggregation of proteins. Nucleic Acids Res..

[202] Szklarczyk D., Franceschini A., Wyder S., Forslund K., Heller D., Huerta-Cepas J., Simonovic M., Roth A., Santos A., Tsafou K.P. (2015). STRING v10: Protein-protein interaction networks, integrated over the tree of life. Nucleic Acids Res..

[203] Vasak M., Hasler D.W. (2000). Metallothioneins: New functional and structural insights. Curr. Opin. Chem. Biol..

[204] Quaife C.J., Findley S.D., Erickson J.C., Froelick G.J., Kelly E.J., Zambrowicz B.P., Palmiter R.D. (1994). Induction of a new metallothionein isoform (MT-IV) occurs during differentiation of stratified squamous epithelia. Biochemistry.

[205] Moffatt P., Denizeau F. (1997). Metallothionein in physiological and physiopathological processes. Drug Metab. Rev..

[206] Ding Z.C., Zheng Q., Cai B., Ni F.Y., Yu W.H., Teng X.C., Gao Y., Liu F., Chen D., Wang Y. (2008). Study on structure-property-reactivity-function relationship of human neuronal growth inhibitory factor (hGIF). J. Inorg. Biochem..

[207] Bogumil R., Faller P., Binz P.A., Vasak M., Charnock J.M., Garner C.D. (1998). Structural characterization of Cu(I) and Zn(II) sites in neuronal-growth-inhibitory factor by extended X-ray absorption fine structure (EXAFS). Eur. J. Biochem..

[208] Sewell A.K., Jensen L.T., Erickson J.C., Palmiter R.D., Winge D.R. (1995). Bioactivity of metallothionein-3 correlates with its novel beta domain sequence rather than metal binding properties. Biochemistry.

[209] Hasler D.W., Jensen L.T., Zerbe O., Winge D.R., Vasak M. (2000). Effect of the two conserved prolines of human growth inhibitory factor (metallothionein-3) on its biological activity and structure fluctuation: Comparison with a mutant protein. Biochemistry.

[210] Romero-Isart N., Jensen L.T., Zerbe O., Winge D.R., Vasak M. (2002). Engineering of metallothionein-3 neuroinhibitory activity into the inactive isoform metallothionein-1. J. Biol. Chem..

[211] Uchida Y., Takio K., Titani K., Ihara Y., Tomonaga M. (1991). The growth inhibitory factor that is deficient in the Alzheimer’s disease brain is a 68 amino acid metallothionein-like protein. Neuron.

[212] Vasak M., Meloni G. (2017). Mammalian Metallothionein-3: New Functional and Structural Insights. Int. J. Mol. Sci..

[213] Jiang Z., Shen B., Xiang J. (2019). Metal-dependent interactions of metallothionein-3 beta-domain with amyloid-beta peptide and related physiological implications. J. Inorg. Biochem..

[214] Koh J.Y., Lee S.J. (2020). Metallothionein-3 as a multifunctional player in the control of cellular processes and diseases. Mol. Brain.

[215] Uchida Y. (1994). Growth-inhibitory factor, metallothionein-like protein, and neurodegenerative diseases. Biol. Signals.

[216] Howells C., West A.K., Chung R.S. (2010). Neuronal growth-inhibitory factor (metallothionein-3): Evaluation of the biological function of growth-inhibitory factor in the injured and neurodegenerative brain. FEBS J..

[217] Wang H., Zhang Q., Cai B., Li H., Sze K.H., Huang Z.X., Wu H.M., Sun H. (2006). Solution structure and dynamics of human metallothionein-3 (MT-3). FEBS Lett..

[218] Oz G., Zangger K., Armitage I.M. (2001). Three-dimensional structure and dynamics of a brain specific growth inhibitory factor: Metallothionein-3. Biochemistry.

[219] Yuan A.T., Korkola N.C., Stillman M.J. (2023). Apo-metallothionein-3 cooperatively forms tightly compact structures under physiological conditions. J. Biol. Chem..

[220] Meszaros B., Erdos G., Dosztanyi Z. (2018). IUPred2A: Context-dependent prediction of protein disorder as a function of redox state and protein binding. Nucleic Acids Res..

[221] Pountney D.L., Dickson T.C., Power J.H., Vickers J.C., West A.J., Gai W.P. (2011). Association of metallothionein-III with oligodendroglial cytoplasmic inclusions in multiple system atrophy. Neurotox. Res..

[222] Ugbode C., West R.J.H. (2021). Lessons learned from CHMP2B, implications for frontotemporal dementia and amyotrophic lateral sclerosis. Neurobiol. Dis..

[223] Rusten T.E., Stenmark H. (2009). How do ESCRT proteins control autophagy?. J. Cell Sci..

[224] Bhutta M.S., McInerny C.J., Gould G.W. (2014). ESCRT function in cytokinesis: Location, dynamics and regulation by mitotic kinases. Int. J. Mol. Sci..

[225] Caballe A., Martin-Serrano J. (2011). ESCRT machinery and cytokinesis: The road to daughter cell separation. Traffic.

[226] Radulovic M., Schink K.O., Wenzel E.M., Nahse V., Bongiovanni A., Lafont F., Stenmark H. (2018). ESCRT-mediated lysosome repair precedes lysophagy and promotes cell survival. EMBO J..

[227] Lata S., Schoehn G., Solomons J., Pires R., Gottlinger H.G., Weissenhorn W. (2009). Structure and function of ESCRT-III. Biochem. Soc. Trans..

[228] Krasniak C.S., Ahmad S.T. (2016). The role of CHMP2B(Intron5) in autophagy and frontotemporal dementia. Brain Res..

[229] Skibinski G., Parkinson N.J., Brown J.M., Chakrabarti L., Lloyd S.L., Hummerich H., Nielsen J.E., Hodges J.R., Spillantini M.G., Thusgaard T. (2005). Mutations in the endosomal ESCRTIII-complex subunit CHMP2B in frontotemporal dementia. Nat. Genet..

[230] Bugiani O. (2007). The many ways to frontotemporal degeneration and beyond. Neurol. Sci..

[231] Urwin H., Ghazi-Noori S., Collinge J., Isaacs A. (2009). The role of CHMP2B in frontotemporal dementia. Biochem. Soc. Trans..

[232] Siuda J., Fujioka S., Wszolek Z.K. (2014). Parkinsonian syndrome in familial frontotemporal dementia. Park. Relat. Disord..

[233] Babst M., Wendland B., Estepa E.J., Emr S.D. (1998). The Vps4p AAA ATPase regulates membrane association of a Vps protein complex required for normal endosome function. EMBO J..

[234] Bodon G., Chassefeyre R., Pernet-Gallay K., Martinelli N., Effantin G., Hulsik D.L., Belly A., Goldberg Y., Chatellard-Causse C., Blot B. (2011). Charged multivesicular body protein 2B (CHMP2B) of the endosomal sorting complex required for transport-III (ESCRT-III) polymerizes into helical structures deforming the plasma membrane. J. Biol. Chem..

[235] Stuchell-Brereton M.D., Skalicky J.J., Kieffer C., Karren M.A., Ghaffarian S., Sundquist W.I. (2007). ESCRT-III recognition by VPS4 ATPases. Nature.

[236] Xiang Y., Xin J., Le W., Yang Y. (2020). Neurogranin: A Potential Biomarker of Neurological and Mental Diseases. Front. Aging Neurosci..

[237] Represa A., Deloulme J.C., Sensenbrenner M., Ben-Ari Y., Baudier J. (1990). Neurogranin: Immunocytochemical localization of a brain-specific protein kinase C substrate. J. Neurosci..

[238] Chen S.J., Klann E., Gower M.C., Powell C.M., Sessoms J.S., Sweatt J.D. (1993). Studies with synthetic peptide substrates derived from the neuronal protein neurogranin reveal structural determinants of potency and selectivity for protein kinase C. Biochemistry.

[239] Baudier J., Deloulme J.C., Van Dorsselaer A., Black D., Matthes H.W. (1991). Purification and characterization of a brain-specific protein kinase C substrate, neurogranin (p17). Identification of a consensus amino acid sequence between neurogranin and neuromodulin (GAP43) that corresponds to the protein kinase C phosphorylation site and the calmodulin-binding domain. J. Biol. Chem..

[240] Gerendasy D.D., Herron S.R., Watson J.B., Sutcliffe J.G. (1994). Mutational and biophysical studies suggest RC3/neurogranin regulates calmodulin availability. J. Biol. Chem..

[241] Chakravarthy B., Morley P., Whitfield J. (1999). Ca^2+^-calmodulin and protein kinase Cs: A hypothetical synthesis of their conflicting convergences on shared substrate domains. Trends Neurosci..

[242] Gerendasy D. (1999). Homeostatic tuning of Ca^2+^ signal transduction by members of the calpacitin protein family. J. Neurosci. Res..

[243] Fyfe I. (2015). Alzheimer disease: Neurogranin in the CSF signals early Alzheimer disease and predicts disease progression. Nat. Rev. Neurol..

[244] Hellwig K., Kvartsberg H., Portelius E., Andreasson U., Oberstein T.J., Lewczuk P., Blennow K., Kornhuber J., Maler J.M., Zetterberg H. (2015). Neurogranin and YKL-40: Independent markers of synaptic degeneration and neuroinflammation in Alzheimer’s disease. Alzheimer’s Res. Ther..

[245] Kester M.I., Teunissen C.E., Crimmins D.L., Herries E.M., Ladenson J.H., Scheltens P., van der Flier W.M., Morris J.C., Holtzman D.M., Fagan A.M. (2015). Neurogranin as a Cerebrospinal Fluid Biomarker for Synaptic Loss in Symptomatic Alzheimer Disease. JAMA Neurol..

[246] Tarawneh R., D’Angelo G., Crimmins D., Herries E., Griest T., Fagan A.M., Zipfel G.J., Ladenson J.H., Morris J.C., Holtzman D.M. (2016). Diagnostic and Prognostic Utility of the Synaptic Marker Neurogranin in Alzheimer Disease. JAMA Neurol..

[247] Portelius E., Zetterberg H., Skillback T., Tornqvist U., Andreasson U., Trojanowski J.Q., Weiner M.W., Shaw L.M., Mattsson N., Blennow K. (2015). Cerebrospinal fluid neurogranin: Relation to cognition and neurodegeneration in Alzheimer’s disease. Brain.

[248] Kvartsberg H., Duits F.H., Ingelsson M., Andreasen N., Ohrfelt A., Andersson K., Brinkmalm G., Lannfelt L., Minthon L., Hansson O. (2015). Cerebrospinal fluid levels of the synaptic protein neurogranin correlates with cognitive decline in prodromal Alzheimer’s disease. Alzheimer’s Dement..

[249] Portelius E., Olsson B., Hoglund K., Cullen N.C., Kvartsberg H., Andreasson U., Zetterberg H., Sandelius A., Shaw L.M., Lee V.M.Y. (2018). Cerebrospinal fluid neurogranin concentration in neurodegeneration: Relation to clinical phenotypes and neuropathology. Acta Neuropathol..

[250] Blennow K., Diaz-Lucena D., Zetterberg H., Villar-Pique A., Karch A., Vidal E., Hermann P., Schmitz M., Ferrer Abizanda I., Zerr I. (2019). CSF neurogranin as a neuronal damage marker in CJD: A comparative study with AD. J. Neurol. Neurosurg. Psychiatry.

[251] Hodges A., Strand A.D., Aragaki A.K., Kuhn A., Sengstag T., Hughes G., Elliston L.A., Hartog C., Goldstein D.R., Thu D. (2006). Regional and cellular gene expression changes in human Huntington’s disease brain. Hum. Mol. Genet..

[252] Runne H., Kuhn A., Wild E.J., Pratyaksha W., Kristiansen M., Isaacs J.D., Regulier E., Delorenzi M., Tabrizi S.J., Luthi-Carter R. (2007). Analysis of potential transcriptomic biomarkers for Huntington’s disease in peripheral blood. Proc. Natl. Acad. Sci. USA.

[253] Lista S., Santos-Lozano A., Emanuele E., Mercuri N.B., Gabelle A., Lopez-Ortiz S., Martin-Hernandez J., Maisto N., Imbimbo C., Caraci F. (2024). Monitoring synaptic pathology in Alzheimer’s disease through fluid and PET imaging biomarkers: A comprehensive review and future perspectives. Mol. Psychiatry.

[254] Dong R., Lu Q., Kang H., Suridjan I., Kollmorgen G., Wild N., Deming Y., Van Hulle C.A., Anderson R.M., Zetterberg H. (2023). CSF metabolites associated with biomarkers of Alzheimer’s disease pathology. Front. Aging Neurosci..

[255] Nilsson J., Gobom J., Sjodin S., Brinkmalm G., Ashton N.J., Svensson J., Johansson P., Portelius E., Zetterberg H., Blennow K. (2021). Cerebrospinal fluid biomarker panel for synaptic dysfunction in Alzheimer’s disease. Alzheimer’s Dement..

[256] Piccoli T., Blandino V., Maniscalco L., Matranga D., Graziano F., Guajana F., Agnello L., Lo Sasso B., Gambino C.M., Giglio R.V. (2022). Biomarkers Related to Synaptic Dysfunction to Discriminate Alzheimer’s Disease from Other Neurological Disorders. Int. J. Mol. Sci..

[257] Chang D.K., Chien W.J., Arunkumar A.I. (1997). Conformation of a protein kinase C substrate NG(28-43), and its analog in aqueous and sodium dodecyl sulfate micelle solutions. Biophys. J..

[258] Ran X., Miao H.H., Sheu F.S., Yang D. (2003). Structural and dynamic characterization of a neuron-specific protein kinase C substrate, neurogranin. Biochemistry.

[259] Ishizuka T., Saisu H., Odani S., Abe T. (1995). Synaphin: A protein associated with the docking/fusion complex in presynaptic terminals. Biochem. Biophys. Res. Commun..

[260] McMahon H.T., Missler M., Li C., Sudhof T.C. (1995). Complexins: Cytosolic proteins that regulate SNAP receptor function. Cell.

[261] Takahashi S., Yamamoto H., Matsuda Z., Ogawa M., Yagyu K., Taniguchi T., Miyata T., Kaba H., Higuchi T., Okutani F. (1995). Identification of two highly homologous presynaptic proteins distinctly localized at the dendritic and somatic synapses. FEBS Lett..

[262] Ono S., Baux G., Sekiguchi M., Fossier P., Morel N.F., Nihonmatsu I., Hirata K., Awaji T., Takahashi S., Takahashi M. (1998). Regulatory roles of complexins in neurotransmitter release from mature presynaptic nerve terminals. Eur. J. Neurosci..

[263] Yamada M., Saisu H., Ishizuka T., Takahashi H., Abe T. (1999). Immunohistochemical distribution of the two isoforms of synaphin/complexin involved in neurotransmitter release: Localization at the distinct central nervous system regions and synaptic types. Neuroscience.

[264] Itakura M., Misawa H., Sekiguchi M., Takahashi S., Takahashi M. (1999). Transfection analysis of functional roles of complexin I and II in the exocytosis of two different types of secretory vesicles. Biochem. Biophys. Res. Commun..

[265] Reim K., Mansour M., Varoqueaux F., McMahon H.T., Sudhof T.C., Brose N., Rosenmund C. (2001). Complexins regulate a late step in Ca^2+^-dependent neurotransmitter release. Cell.

[266] Krishnakumar S.S., Radoff D.T., Kummel D., Giraudo C.G., Li F., Khandan L., Baguley S.W., Coleman J., Reinisch K.M., Pincet F. (2011). A conformational switch in complexin is required for synaptotagmin to trigger synaptic fusion. Nat. Struct. Mol. Biol..

[267] Lottermoser J.A., Dittman J.S. (2023). Complexin Membrane Interactions: Implications for Synapse Evolution and Function. J. Mol. Biol..

[268] Gispert S., Kurz A., Brehm N., Rau K., Walter M., Riess O., Auburger G. (2015). Complexin-1 and Foxp1 Expression Changes Are Novel Brain Effects of A-Synuclein Pathology. Mol. Neurobiol..

[269] Xue M., Reim K., Chen X., Chao H.T., Deng H., Rizo J., Brose N., Rosenmund C. (2007). Distinct domains of complexin I differentially regulate neurotransmitter release. Nat. Struct. Mol. Biol..

[270] Hobson R.J., Liu Q., Watanabe S., Jorgensen E.M. (2011). Complexin maintains vesicles in the primed state in C. elegans. Curr. Biol..

[271] Martin J.A., Hu Z., Fenz K.M., Fernandez J., Dittman J.S. (2011). Complexin has opposite effects on two modes of synaptic vesicle fusion. Curr. Biol..

[272] Lai Y., Choi U.B., Zhang Y., Zhao M., Pfuetzner R.A., Wang A.L., Diao J., Brunger A.T. (2016). N-terminal domain of complexin independently activates calcium-triggered fusion. Proc. Natl. Acad. Sci. USA.

[273] Zdanowicz R., Kreutzberger A., Liang B., Kiessling V., Tamm L.K., Cafiso D.S. (2017). Complexin Binding to Membranes and Acceptor t-SNAREs Explains Its Clamping Effect on Fusion. Biophys. J..

[274] Bera M., Ramakrishnan S., Coleman J., Krishnakumar S.S., Rothman J.E. (2022). Molecular determinants of complexin clamping and activation function. eLife.

[275] Pabst S., Hazzard J.W., Antonin W., Sudhof T.C., Jahn R., Rizo J., Fasshauer D. (2000). Selective interaction of complexin with the neuronal SNARE complex. Determination of the binding regions. J. Biol. Chem..

[276] Chen X., Tomchick D.R., Kovrigin E., Arac D., Machius M., Sudhof T.C., Rizo J. (2002). Three-dimensional structure of the complexin/SNARE complex. Neuron.

[277] Bowen M.E., Weninger K., Ernst J., Chu S., Brunger A.T. (2005). Single-molecule studies of synaptotagmin and complexin binding to the SNARE complex. Biophys. J..

[278] Malsam J., Seiler F., Schollmeier Y., Rusu P., Krause J.M., Sollner T.H. (2009). The carboxy-terminal domain of complexin I stimulates liposome fusion. Proc. Natl. Acad. Sci. USA.

[279] Kaeser-Woo Y.J., Yang X., Sudhof T.C. (2012). C-terminal complexin sequence is selectively required for clamping and priming but not for Ca2+ triggering of synaptic exocytosis. J. Neurosci..

[280] Wragg R.T., Snead D., Dong Y., Ramlall T.F., Menon I., Bai J., Eliezer D., Dittman J.S. (2013). Synaptic vesicles position complexin to block spontaneous fusion. Neuron.

[281] Snead D., Wragg R.T., Dittman J.S., Eliezer D. (2014). Membrane curvature sensing by the C-terminal domain of complexin. Nat. Commun..

[282] Gong J., Lai Y., Li X., Wang M., Leitz J., Hu Y., Zhang Y., Choi U.B., Cipriano D., Pfuetzner R.A. (2016). C-terminal domain of mammalian complexin-1 localizes to highly curved membranes. Proc. Natl. Acad. Sci. USA.

[283] Courtney K.C., Wu L., Mandal T., Swift M., Zhang Z., Alaghemandi M., Wu Z., Bradberry M.M., Deo C., Lavis L.D. (2022). The complexin C-terminal amphipathic helix stabilizes the fusion pore open state by sculpting membranes. Nat. Struct. Mol. Biol..

[284] Trimbuch T., Rosenmund C. (2016). Should I stop or should I go? The role of complexin in neurotransmitter release. Nat. Rev. Neurosci..

[285] Falkowski M.A., Thomas D.D., Groblewski G.E. (2010). Complexin 2 modulates vesicle-associated membrane protein (VAMP) 2-regulated zymogen granule exocytosis in pancreatic acini. J. Biol. Chem..

[286] Tadokoro S., Nakanishi M., Hirashima N. (2005). Complexin II facilitates exocytotic release in mast cells by enhancing Ca^2+^ sensitivity of the fusion process. J. Cell Sci..

[287] Tsuru E., Oryu K., Sawada K., Nishihara M., Tsuda M. (2019). Complexin 2 regulates secretion of immunoglobulin in antibody-secreting cells. Immun. Inflamm. Dis..

[288] Tsai P.S., Brewis I.A., van Maaren J., Gadella B.M. (2012). Involvement of complexin 2 in docking, locking and unlocking of different SNARE complexes during sperm capacitation and induced acrosomal exocytosis. PLoS ONE.

[289] DiProspero N.A., Chen E.Y., Charles V., Plomann M., Kordower J.H., Tagle D.A. (2004). Early changes in Huntington’s disease patient brains involve alterations in cytoskeletal and synaptic elements. J. Neurocytol..

[290] Parplys A.C., Zhao W., Sharma N., Groesser T., Liang F., Maranon D.G., Leung S.G., Grundt K., Dray E., Idate R. (2015). NUCKS1 is a novel RAD51AP1 paralog important for homologous recombination and genome stability. Nucleic Acids Res..

[291] Symonowicz K., Dus-Szachniewicz K., Wozniak M., Murawski M., Kolodziej P., Osiecka B., Jurczyszyn K., Ziolkowski P. (2014). Immunohistochemical study of nuclear ubiquitous casein and cyclin-dependent kinase substrate 1 in invasive breast carcinoma of no special type. Exp. Ther. Med..

[292] Drosos Y., Kouloukoussa M., Ostvold A.C., Grundt K., Goutas N., Vlachodimitropoulos D., Havaki S., Kollia P., Kittas C., Marinos E. (2009). NUCKS overexpression in breast cancer. Cancer Cell Int..

[293] Cheong J.Y., Kim Y.B., Woo J.H., Kim D.K., Yeo M., Yang S.J., Yang K.S., Soon S.K., Wang H.J., Kim B.W. (2016). Identification of NUCKS1 as a putative oncogene and immunodiagnostic marker of hepatocellular carcinoma. Gene.

[294] Zhang X., Zhang X., Li X., Bao H., Li G., Li N., Li H., Dou J. (2023). NUCKS1 Acts as a Promising Novel Biomarker for the Prognosis of Patients with Hepatocellular Carcinoma. Cancer Biother. Radiopharm..

[295] Shi C., Qin L., Gao H., Gu L., Yang C., Liu H., Liu T. (2017). NUCKS nuclear elevated expression indicates progression and prognosis of ovarian cancer. Tumour Biol..

[296] Huang Y.K., Kang W.M., Ma Z.Q., Liu Y.Q., Zhou L., Yu J.C. (2019). NUCKS1 promotes gastric cancer cell aggressiveness by upregulating IGF-1R and subsequently activating the PI3K/Akt/mTOR signaling pathway. Carcinogenesis.

[297] Gu L., Xia B., Zhong L., Ma Y., Liu L., Yang L., Lou G. (2014). NUCKS1 overexpression is a novel biomarker for recurrence-free survival in cervical squamous cell carcinoma. Tumour Biol..

[298] Zhu L.L., Shi J.J., Guo Y.D., Yang C., Wang R.L., Li S.S., Gan D.X., Ma P.X., Li J.Q., Su H.C. (2023). NUCKS1 promotes the progression of colorectal cancer via activating PI3K/AKT/mTOR signaling pathway. Neoplasma.

[299] Zheng S., Ji R., He H., Li N., Han C., Han J., Li X., Zhang L., Wang Y., Zhao W. (2023). NUCKS1, a LINC00629-upregulated gene, facilitated osteosarcoma progression and metastasis by elevating asparagine synthesis. Cell Death Dis..

[300] Ma H., Xu J., Zhao R., Qi Y., Ji Y., Ma K. (2021). Upregulation of NUCKS1 in Lung Adenocarcinoma is Associated with a Poor Prognosis. Cancer Investig..

[301] Kim H.Y., Choi B.S., Kim S.S., Roh T.Y., Park J., Yoon C.H. (2014). NUCKS1, a novel Tat coactivator, plays a crucial role in HIV-1 replication by increasing Tat-mediated viral transcription on the HIV-1 LTR promoter. Retrovirology.

[302] Qiu B., Han W., Tergaonkar V. (2015). NUCKS: A potential biomarker in cancer and metabolic disease. Clin. Sci..

[303] Wang L., Cheng L., Lu Z.J., Sun X.Y., Li J.Y., Peng R. (2016). Association of three candidate genetic variants in RAB7L1/NUCKS1, MCCC1 and STK39 with sporadic Parkinson’s disease in Han Chinese. J. Neural. Transm..

[304] Singh S., Seth P.K. (2019). Functional association between NUCKS1 gene and Parkinson disease: A potential susceptibility biomarker. Bioinformation.

[305] Xu L., Xia C., Sun W., Qin X., Qiu Y., Zhu Z. (2017). Genetic Polymorphism of NUCKS1 Is Associated with the Susceptibility of Adolescent Idiopathic Scoliosis. Spine.

[306] Ostvold A.C., Holtlund J., Laland S.G. (1985). A novel, highly phosphorylated protein, of the high-mobility group type, present in a variety of proliferating and non-proliferating mammalian cells. Eur. J. Biochem..

[307] Maelandsmo G.M., Ostvold A.C., Laland S.G. (1989). Phosphorylation of the high-mobility-group-like protein P1 by casein kinase-2. Eur. J. Biochem..

[308] Ostvold A.C., Norum J.H., Mathiesen S., Wanvik B., Sefland I., Grundt K. (2001). Molecular cloning of a mammalian nuclear phosphoprotein NUCKS, which serves as a substrate for Cdk1 in vivo. Eur. J. Biochem..

[309] Arroyo E.J., Scherer S.S. (2000). On the molecular architecture of myelinated fibers. Histochem. Cell Biol..

[310] Rosenbluth J., Mierzwa A., Shroff S. (2013). Molecular architecture of myelinated nerve fibers: Leaky paranodal junctions and paranodal dysmyelination. Neuroscientist.

[311] Tzimourakas A., Giasemi S., Mouratidou M., Karagogeos D. (2007). Structure-function analysis of protein complexes involved in the molecular architecture of juxtaparanodal regions of myelinated fibers. Biotechnol. J..

[312] Baumann N., Pham-Dinh D. (2001). Biology of oligodendrocyte and myelin in the mammalian central nervous system. Physiol. Rev..

[313] Harauz G., Ishiyama N., Hill C.M., Bates I.R., Libich D.S., Fares C. (2004). Myelin basic protein-diverse conformational states of an intrinsically unstructured protein and its roles in myelin assembly and multiple sclerosis. Micron.

[314] Kramer E.M., Schardt A., Nave K.A. (2001). Membrane traffic in myelinating oligodendrocytes. Microsc. Res. Technol..

[315] Kon T., Tanji K., Mori F., Kimura A., Kakita A., Wakabayashi K. (2019). Immunoreactivity of myelin-associated oligodendrocytic basic protein in Lewy bodies. Neuropathology.

[316] Wong J.H., Halliday G.M., Kim W.S. (2014). Exploring myelin dysfunction in multiple system atrophy. Exp. Neurobiol..

[317] Smith R. (1992). The basic protein of CNS myelin: Its structure and ligand binding. J. Neurochem..

[318] Hill C.M., Bates I.R., White G.F., Hallett F.R., Harauz G. (2002). Effects of the osmolyte trimethylamine-N-oxide on conformation, self-association, and two-dimensional crystallization of myelin basic protein. J. Struct. Biol..

[319] Hill C.M., Haines J.D., Antler C.E., Bates I.R., Libich D.S., Harauz G. (2003). Terminal deletion mutants of myelin basic protein: New insights into self-association and phospholipid interactions. Micron.

[320] Sedzik J., Kirschner D.A. (1992). Is myelin basic protein crystallizable?. Neurochem. Res..

[321] Nixon R.A., Saito K.I., Grynspan F., Griffin W.R., Katayama S., Honda T., Mohan P.S., Shea T.B., Beermann M. (1994). Calcium-activated neutral proteinase (calpain) system in aging and Alzheimer’s disease. Ann. N. Y. Acad. Sci..

[322] Huang Y., Wang K.K. (2001). The calpain family and human disease. Trends Mol. Med..

[323] Suzuki K., Hata S., Kawabata Y., Sorimachi H. (2004). Structure, activation, and biology of calpain. Diabetes.

[324] Mellgren R.L., Rozanov C.B. (1990). Calpain II-dependent solubilization of a nuclear protein kinase at micromolar calcium concentrations. Biochem. Biophys. Res. Commun..

[325] Chakrabarti A.K., Dasgupta S., Banik N.L., Hogan E.L. (1990). Regulation of the calcium-activated neutral proteinase (CANP) of bovine brain by myelin lipids. Biochim. Biophys. Acta.

[326] Saido T.C., Shibata M., Takenawa T., Murofushi H., Suzuki K. (1992). Positive regulation of mu-calpain action by polyphosphoinositides. J. Biol. Chem..

[327] Salamino F., De Tullio R., Mengotti P., Viotti P.L., Melloni E., Pontremoli S. (1993). Site-directed activation of calpain is promoted by a membrane-associated natural activator protein. Biochem. J..

[328] Suzuki K., Ohno S. (1990). Calcium activated neutral protease—Structure-function relationship and functional implications. Cell Struct. Funct..

[329] Murachi T. (1989). Intracellular regulatory system involving calpain and calpastatin. Biochem. Int..

[330] Nixon R.A. (1989). Calcium-activated neutral proteinases as regulators of cellular function. Implications for Alzheimer’s disease pathogenesis. Ann. N. Y. Acad. Sci..

[331] Nixon R.A., Quackenbush R., Vitto A. (1986). Multiple calcium-activated neutral proteinases (CANP) in mouse retinal ganglion cell neurons: Specificities for endogenous neuronal substrates and comparison to purified brain CANP. J. Neurosci..

[332] Lee W.J., Ma H., Takano E., Yang H.Q., Hatanaka M., Maki M. (1992). Molecular diversity in amino-terminal domains of human calpastatin by exon skipping. J. Biol. Chem..

[333] Adachi Y., Ishida-Takahashi A., Takahashi C., Takano E., Murachi T., Hatanaka M. (1991). Phosphorylation and subcellular distribution of calpastatin in human hematopoietic system cells. J. Biol. Chem..

[334] Nakamura M., Inomata M., Imajoh S., Suzuki K., Kawashima S. (1989). Fragmentation of an endogenous inhibitor upon complex formation with high- and low-Ca^2+^-requiring forms of calcium-activated neutral proteases. Biochemistry.

[335] Emori Y., Kawasaki H., Imajoh S., Minami Y., Suzuki K. (1988). All four repeating domains of the endogenous inhibitor for calcium-dependent protease independently retain inhibitory activity. Expression of the cDNA fragments in *Escherichia coli*. J. Biol. Chem..

[336] Wendt A., Thompson V.F., Goll D.E. (2004). Interaction of calpastatin with calpain: A review. Biol. Chem..

[337] Takano E., Ma H., Yang H.Q., Maki M., Hatanaka M. (1995). Preference of calcium-dependent interactions between calmodulin-like domains of calpain and calpastatin subdomains. FEBS Lett..

[338] Tompa P., Mucsi Z., Orosz G., Friedrich P. (2002). Calpastatin subdomains A and C are activators of calpain. J. Biol. Chem..

[339] Uemori T., Shimojo T., Asada K., Asano T., Kimizuka F., Kato I., Maki M., Hatanaka M., Murachi T., Hanzawa H. (1990). Characterization of a functional domain of human calpastatin. Biochem. Biophys. Res. Commun..

[340] Konno T., Tanaka N., Kataoka M., Takano E., Maki M. (1997). A circular dichroism study of preferential hydration and alcohol effects on a denatured protein, pig calpastatin domain I. Biochim. Biophys. Acta.

[341] Kiss R., Kovacs D., Tompa P., Perczel A. (2008). Local structural preferences of calpastatin, the intrinsically unstructured protein inhibitor of calpain. Biochemistry.

[342] Ballatore C., Brunden K.R., Huryn D.M., Trojanowski J.Q., Lee V.M., Smith A.B. (2012). Microtubule stabilizing agents as potential treatment for Alzheimer’s disease and related neurodegenerative tauopathies. J. Med. Chem..

[343] Drechsel D.N., Hyman A.A., Cobb M.H., Kirschner M.W. (1992). Modulation of the dynamic instability of tubulin assembly by the microtubule-associated protein tau. Mol. Biol. Cell.

[344] Roy S., Zhang B., Lee V.M., Trojanowski J.Q. (2005). Axonal transport defects: A common theme in neurodegenerative diseases. Acta Neuropathol..

[345] Kuret J., Congdon E.E., Li G., Yin H., Yu X., Zhong Q. (2005). Evaluating triggers and enhancers of tau fibrillization. Microsc. Res. Technol..

[346] Kuret J., Chirita C.N., Congdon E.E., Kannanayakal T., Li G., Necula M., Yin H., Zhong Q. (2005). Pathways of tau fibrillization. Biochim. Biophys. Acta.

[347] Lee V.M., Goedert M., Trojanowski J.Q. (2001). Neurodegenerative tauopathies. Annu. Rev. Neurosci..

[348] Spillantini M.G., Goedert M., Crowther R.A., Murrell J.R., Farlow M.R., Ghetti B. (1997). Familial multiple system tauopathy with presenile dementia: A disease with abundant neuronal and glial tau filaments. Proc. Natl. Acad. Sci. USA.

[349] Lee V.M., Trojanowski J.Q. (1999). Neurodegenerative tauopathies: Human disease and transgenic mouse models. Neuron.

[350] Josephs K.A., Hodges J.R., Snowden J.S., Mackenzie I.R., Neumann M., Mann D.M., Dickson D.W. (2011). Neuropathological background of phenotypical variability in frontotemporal dementia. Acta Neuropathol..

[351] Arendt T., Stieler J.T., Holzer M. (2016). Tau and tauopathies. Brain Res. Bull..

[352] Gotz J., Halliday G., Nisbet R.M. (2019). Molecular Pathogenesis of the Tauopathies. Annu. Rev. Pathol..

[353] Kneynsberg A., Combs B., Christensen K., Morfini G., Kanaan N.M. (2017). Axonal Degeneration in Tauopathies: Disease Relevance and Underlying Mechanisms. Front. Neurosci..

[354] Uemura N., Uemura M.T., Luk K.C., Lee V.M., Trojanowski J.Q. (2020). Cell-to-Cell Transmission of Tau and α-Synuclein. Trends Mol. Med..

[355] Gibbons G.S., Lee V.M.Y., Trojanowski J.Q. (2019). Mechanisms of Cell-to-Cell Transmission of Pathological Tau: A Review. JAMA Neurol..

[356] Drewes G., Trinczek B., Illenberger S., Biernat J., Schmitt-Ulms G., Meyer H.E., Mandelkow E.M., Mandelkow E. (1995). Microtubule-associated protein/microtubule affinity-regulating kinase (p110mark). A novel protein kinase that regulates tau-microtubule interactions and dynamic instability by phosphorylation at the Alzheimer-specific site serine 262. J. Biol. Chem..

[357] Clark L.N., Poorkaj P., Wszolek Z., Geschwind D.H., Nasreddine Z.S., Miller B., Li D., Payami H., Awert F., Markopoulou K. (1998). Pathogenic implications of mutations in the tau gene in pallido-ponto-nigral degeneration and related neurodegenerative disorders linked to chromosome 17. Proc. Natl. Acad. Sci. USA.

[358] Narayanan R.L., Durr U.H., Bibow S., Biernat J., Mandelkow E., Zweckstetter M. (2010). Automatic assignment of the intrinsically disordered protein Tau with 441-residues. J. Am. Chem. Soc..

[359] Mukrasch M.D., Bibow S., Korukottu J., Jeganathan S., Biernat J., Griesinger C., Mandelkow E., Zweckstetter M. (2009). Structural polymorphism of 441-residue tau at single residue resolution. PLoS Biol..

[360] Ambadipudi S., Reddy J.G., Biernat J., Mandelkow E., Zweckstetter M. (2019). Residue-specific identification of phase separation hot spots of Alzheimer’s-related protein tau. Chem. Sci..

[361] Ukmar-Godec T., Wegmann S., Zweckstetter M. (2020). Biomolecular condensation of the microtubule-associated protein tau. Semin. Cell Dev. Biol..

[362] Rai S.K., Savastano A., Singh P., Mukhopadhyay S., Zweckstetter M. (2021). Liquid-liquid phase separation of tau: From molecular biophysics to physiology and disease. Protein Sci..

[363] Chakraborty P., Zweckstetter M. (2022). Phase separation of the microtubule-associated protein tau. Essays Biochem..

[364] Pan L., Li C., Meng L., Tian Y., He M., Yuan X., Zhang G., Zhang Z., Xiong J., Chen G. (2022). Tau accelerates α-synuclein aggregation and spreading in Parkinson’s disease. Brain.

[365] Robinson J.L., Lee E.B., Xie S.X., Rennert L., Suh E., Bredenberg C., Caswell C., Van Deerlin V.M., Yan N., Yousef A. (2018). Neurodegenerative disease concomitant proteinopathies are prevalent, age-related and APOE4-associated. Brain.

[366] Twohig D., Nielsen H.M. (2019). α-synuclein in the pathophysiology of Alzheimer’s disease. Mol. Neurodegener..

[367] Jin M., Wang S., Gao X., Zou Z., Hirotsune S., Sun L. (2024). Pathological and physiological functional cross-talks of α-synuclein and tau in the central nervous system. Neural Regen. Res..

[368] Kayed R., Dettmer U., Lesne S.E. (2020). Soluble endogenous oligomeric α-synuclein species in neurodegenerative diseases: Expression, spreading, and cross-talk. J. Park. Dis..

[369] Lu J., Zhang S., Ma X., Jia C., Liu Z., Huang C., Liu C., Li D. (2020). Structural basis of the interplay between α-synuclein and Tau in regulating pathological amyloid aggregation. J. Biol. Chem..

[370] Williams T., Sorrentino Z., Weinrich M., Giasson B.I., Chakrabarty P. (2020). Differential cross-seeding properties of tau and α-synuclein in mouse models of tauopathy and synucleinopathy. Brain Commun..

[371] Wang S., Fu Y., Miyata T., Matsumoto S., Shinoda T., Itoh K., Harada A., Hirotsune S., Jin M. (2022). Functional Cooperation of α-Synuclein and Tau Is Essential for Proper Corticogenesis. J. Neurosci..

[372] Lu J., Xu W.X., Wang S.Y., Zhan Y.Q., Jiang Y., Cai W.M., Yang X.M. (2001). Isolation and Characterization of EDAG-1, A Novel Gene Related to Regulation in Hematopoietic System. Sheng Wu Hua Xue Yu Sheng Wu Wu Li Xue Bao.

[373] Yang L.V., Nicholson R.H., Kaplan J., Galy A., Li L. (2001). Hemogen is a novel nuclear factor specifically expressed in mouse hematopoietic development and its human homologue EDAG maps to chromosome 9q22, a region containing breakpoints of hematological neoplasms. Mech. Dev..

[374] Liu C.C., Chou Y.L., Ch’ang L.Y. (2004). Down-regulation of human NDR gene in megakaryocytic differentiation of erythroleukemia K562 cells. J. Biomed. Sci..

[375] Li C.Y., Zhan Y.Q., Xu C.W., Xu W.X., Wang S.Y., Lv J., Zhou Y., Yue P.B., Chen B., Yang X.M. (2004). EDAG regulates the proliferation and differentiation of hematopoietic cells and resists cell apoptosis through the activation of nuclear factor-κB. Cell Death Differ..

[376] An L.L., Li G., Wu K.F., Ma X.T., Zheng G.G., Qiu L.G., Song Y.H. (2005). High expression of EDAG and its significance in AML. Leukemia.

[377] Yang L.V., Heng H.H., Wan J., Southwood C.M., Gow A., Li L. (2003). Alternative promoters and polyadenylation regulate tissue-specific expression of Hemogen isoforms during hematopoiesis and spermatogenesis. Dev. Dyn..

[378] Zhou Y., Xu W.X., Zhan Y.Q., Li C.Y., Xu C.W., Zheng H., Li F.F., Yang X.M., Wang S.Y. (2004). Expression of EDAG-1 gene in human leukemia and lymphoma cell lines. Ai Zheng.

[379] Chen D.L., Hu Z.Q., Zheng X.F., Wang X.Y., Xu Y.Z., Li W.Q., Fang H.S., Kan L., Wang S.Y. (2016). EDAG-1 promotes proliferation and invasion of human thyroid cancer cells by activating MAPK/Erk and AKT signal pathways. Cancer Biol. Ther..

[380] Millan-Arino L., Izquierdo-Bouldstridge A., Jordan A. (2016). Specificities and genomic distribution of somatic mammalian histone H1 subtypes. Biochim. Biophys. Acta.

[381] Fyodorov D.V., Zhou B.R., Skoultchi A.I., Bai Y. (2018). Emerging roles of linker histones in regulating chromatin structure and function. Nat. Rev. Mol. Cell Biol..

[382] Salinas-Pena M., Rebollo E., Jordan A. (2024). Imaging analysis of six human histone H1 variants reveals universal enrichment of H1.2, H1.3, and H1.5 at the nuclear periphery and nucleolar H1X presence. eLife.

[383] Cao K., Lailler N., Zhang Y., Kumar A., Uppal K., Liu Z., Lee E.K., Wu H., Medrzycki M., Pan C. (2013). High-resolution mapping of h1 linker histone variants in embryonic stem cells. PLoS Genet..

[384] Izzo A., Kamieniarz-Gdula K., Ramirez F., Noureen N., Kind J., Manke T., van Steensel B., Schneider R. (2013). The genomic landscape of the somatic linker histone subtypes H1.1 to H1.5 in human cells. Cell Rep..

[385] Millan-Arino L., Islam A.B., Izquierdo-Bouldstridge A., Mayor R., Terme J.M., Luque N., Sancho M., Lopez-Bigas N., Jordan A. (2014). Mapping of six somatic linker histone H1 variants in human breast cancer cells uncovers specific features of H1.2. Nucleic Acids Res..

[386] Serna-Pujol N., Salinas-Pena M., Mugianesi F., Le Dily F., Marti-Renom M.A., Jordan A. (2022). Coordinated changes in gene expression, H1 variant distribution and genome 3D conformation in response to H1 depletion. Nucleic Acids Res..

[387] Lai S., Jia J., Cao X., Zhou P.K., Gao S. (2021). Molecular and Cellular Functions of the Linker Histone H1.2. Front. Cell Dev. Biol..

[388] Roque A., Sortino R., Ventura S., Ponte I., Suau P. (2015). Histone H1 Favors Folding and Parallel Fibrillar Aggregation of the 1-42 Amyloid-beta Peptide. Langmuir.

[389] Peng Z., Mizianty M.J., Xue B., Kurgan L., Uversky V.N. (2012). More than just tails: Intrinsic disorder in histone proteins. Mol. Biosyst..

[390] Wisniewski J.R., Zougman A., Kruger S., Mann M. (2007). Mass spectrometric mapping of linker histone H1 variants reveals multiple acetylations, methylations, and phosphorylation as well as differences between cell culture and tissue. Mol. Cell Proteom..

[391] Jiang T., Zhou X., Taghizadeh K., Dong M., Dedon P.C. (2007). N-formylation of lysine in histone proteins as a secondary modification arising from oxidative DNA damage. Proc. Natl. Acad. Sci. USA.

[392] Wisniewski J.R., Zougman A., Mann M. (2008). Nepsilon-formylation of lysine is a widespread post-translational modification of nuclear proteins occurring at residues involved in regulation of chromatin function. Nucleic Acids Res..

[393] Weiss T., Hergeth S., Zeissler U., Izzo A., Tropberger P., Zee B.M., Dundr M., Garcia B.A., Daujat S., Schneider R. (2010). Histone H1 variant-specific lysine methylation by G9a/KMT1C and Glp1/KMT1D. Epigenet. Chromatin.

[394] Izzo A., Schneider R. (2016). The role of linker histone H1 modifications in the regulation of gene expression and chromatin dynamics. Biochim. Biophys. Acta.

[395] Goers J., Manning-Bog A.B., McCormack A.L., Millett I.S., Doniach S., Di Monte D.A., Uversky V.N., Fink A.L. (2003). Nuclear localization of α-synuclein and its interaction with histones. Biochemistry.

[396] Wilk S. (1983). Prolyl endopeptidase. Life Sci..

[397] Mentlein R. (1988). Proline residues in the maturation and degradation of peptide hormones and neuropeptides. FEBS Lett..

[398] Lin L.N., Brandts J.F. (1983). Evidence showing that a proline-specific endopeptidase has an absolute requirement for a trans peptide bond immediately preceding the active bond. Biochemistry.

[399] Haffner C.D., Diaz C.J., Miller A.B., Reid R.A., Madauss K.P., Hassell A., Hanlon M.H., Porter D.J., Becherer J.D., Carter L.H. (2008). Pyrrolidinyl pyridone and pyrazinone analogues as potent inhibitors of prolyl oligopeptidase (POP). Bioorg. Med. Chem. Lett..

[400] Sabha B.H., Alzahrani F., Almehdar H.A., Uversky V.N., Redwan E.M. (2018). Disorder in Milk Proteins: Lactadherin Multifunctionality and Structure. Curr. Protein Pept. Sci..

[401] Larocca D., Peterson J.A., Urrea R., Kuniyoshi J., Bistrain A.M., Ceriani R.L. (1991). A Mr 46,000 human milk fat globule protein that is highly expressed in human breast tumors contains factor VIII-like domains. Cancer Res..

[402] Duran-Jara E., Vera-Tobar T., Lobos-Gonzalez L.L. (2022). Lactadherin: From a Well-Known Breast Tumor Marker to a Possible Player in Extracellular Vesicle-Mediated Cancer Progression. Int. J. Mol. Sci..

[403] Boddaert J., Kinugawa K., Lambert J.C., Boukhtouche F., Zoll J., Merval R., Blanc-Brude O., Mann D., Berr C., Vilar J. (2007). Evidence of a role for lactadherin in Alzheimer’s disease. Am. J. Pathol..

[404] Copland S.D., Murphy A.A., Shur B.D. (2009). The mouse gamete adhesin, SED1, is expressed on the surface of acrosome-intact human sperm. Fertil. Steril..

[405] Pepys M.B., Dyck R.F., de Beer F.C., Skinner M., Cohen A.S. (1979). Binding of serum amyloid P-component (SAP) by amyloid fibrils. Clin. Exp. Immunol..

[406] Emsley J., White H.E., O’Hara B.P., Oliva G., Srinivasan N., Tickle I.J., Blundell T.L., Pepys M.B., Wood S.P. (1994). Structure of pentameric human serum amyloid P component. Nature.

[407] Pepys M.B., Booth D., Hutchinson W., Gallimore J., Collins I., Hohenester E. (1997). Amyloid P component. A critical review. Amyloid.

[408] Hutchinson W.L., Hohenester E., Pepys M.B. (2000). Human serum amyloid P component is a single uncomplexed pentamer in whole serum. Mol. Med..

[409] Cho K., Pham T.N., Crivello S.D., Jeong J., Green T.L., Greenhalgh D.G. (2004). Involvement of CD14 and toll-like receptor 4 in the acute phase response of serum amyloid A proteins and serum amyloid P component in the liver after burn injury. Shock.

[410] Pilling D., Gomer R.H. (2018). The Development of Serum Amyloid P as a Possible Therapeutic. Front. Immunol..

[411] Kolstoe S.E., Jenvey M.C., Purvis A., Light M.E., Thompson D., Hughes P., Pepys M.B., Wood S.P. (2014). Interaction of serum amyloid P component with hexanoyl bis(D-proline) (CPHPC). Acta Crystallogr. D Biol. Crystallogr..

[412] Lockridge O., Masson P. (2000). Pesticides and susceptible populations: People with butyrylcholinesterase genetic variants may be at risk. Neurotoxicology.

[413] Fukami T., Yokoi T. (2012). The emerging role of human esterases. Drug Metab. Pharmacokinet..

[414] Behra M., Cousin X., Bertrand C., Vonesch J.L., Biellmann D., Chatonnet A., Strahle U. (2002). Acetylcholinesterase is required for neuronal and muscular development in the zebrafish embryo. Nat. Neurosci..

[415] Meshorer E., Erb C., Gazit R., Pavlovsky L., Kaufer D., Friedman A., Glick D., Ben-Arie N., Soreq H. (2002). Alternative splicing and neuritic mRNA translocation under long-term neuronal hypersensitivity. Science.

[416] Pope C.N., Brimijoin S. (2018). Cholinesterases and the fine line between poison and remedy. Biochem. Pharmacol..

[417] Sridhar G.R., Gumpeny L. (2024). Emerging significance of butyrylcholinesterase. World J. Exp. Med..

[418] Sadeghi M., Seyedebrahimi S., Ghanadian M., Miroliaei M. (2024). Identification of cholinesterases inhibitors from flavonoids derivatives for possible treatment of Alzheimer’s disease: In silico and in vitro approaches. Curr. Res. Struct. Biol..

[419] Ngamelue M.N., Homma K., Lockridge O., Asojo O.A. (2007). Crystallization and X-ray structure of full-length recombinant human butyrylcholinesterase. Acta Crystallogr. Sect. F Struct. Biol. Cryst. Commun..

[420] Nicolet Y., Lockridge O., Masson P., Fontecilla-Camps J.C., Nachon F. (2003). Crystal structure of human butyrylcholinesterase and of its complexes with substrate and products. J. Biol. Chem..

[421] Bourges I., Ramus C., Mousson de Camaret B., Beugnot R., Remacle C., Cardol P., Hofhaus G., Issartel J.P. (2004). Structural organization of mitochondrial human complex I: Role of the ND4 and ND5 mitochondria-encoded subunits and interaction with prohibitin. Biochem. J..

[422] Lenaz G., Baracca A., Fato R., Genova M.L., Solaini G. (2006). Mitochondrial Complex I: Structure, function, and implications in neurodegeneration. Ital. J. Biochem..

[423] Schapira A.H. (2010). Complex I: Inhibitors, inhibition and neurodegeneration. Exp. Neurol..

[424] Giachin G., Bouverot R., Acajjaoui S., Pantalone S., Soler-Lopez M. (2016). Dynamics of Human Mitochondrial Complex I Assembly: Implications for Neurodegenerative Diseases. Front. Mol. Biosci..

[425] Monzio Compagnoni G., Di Fonzo A., Corti S., Comi G.P., Bresolin N., Masliah E. (2020). The Role of Mitochondria in Neurodegenerative Diseases: The Lesson from Alzheimer’s Disease and Parkinson’s Disease. Mol. Neurobiol..

[426] Novack G.V., Galeano P., Castano E.M., Morelli L. (2020). Mitochondrial Supercomplexes: Physiological Organization and Dysregulation in Age-Related Neurodegenerative Disorders. Front. Endocrinol..

[427] Chavda V., Lu B. (2023). Reverse Electron Transport at Mitochondrial Complex I in Ischemic Stroke, Aging, and Age-Related Diseases. Antioxidants.

[428] Subrahmanian N., LaVoie M.J. (2021). Is there a special relationship between complex I activity and nigral neuronal loss in Parkinson’s disease? A critical reappraisal. Brain Res..

[429] Flones I.H., Nyland H., Sandnes D.A., Alves G.W., Tysnes O.B., Tzoulis C. (2022). Early Forms of α-Synuclein Pathology Are Associated with Neuronal Complex I Deficiency in the Substantia Nigra of Individuals with Parkinson’s Disease. Biomolecules.

