# Supplementary material for: Exploring Intrinsic Disorder in Human Synucleins and Associated Proteins
Source: Int J Mol Sci. 2024 Aug 1;25(15):8399. doi: 10.3390/ijms25158399 (PMC11313516; doi:10.3390/ijms25158399)
Supplement: Supplementary file 1 [file ijms-25-08399-s001.zip › ijms-3090837-supplementary.pdf]

Supplementary Materials

Exploring intrinsic disorder in human synucleins and associated proteins

Sriya Reddy Venati [1], Vladimir N. Uversky [1,2,*]

1 Department of Molecular Medicine, Morsani College of Medicine, University of South Florida, Tampa, FL 33612, USA; sriyareddyvenati@usf.edu (S.R.V.); vuversky@usf.edu (V.N.U.)
2 USF Health Byrd Alzheimer's Research Institute, Morsani College of Medicine, University of South Florida, Tampa, FL 33612, USA; vuversky@usf.edu
* Correspondence: Department of Molecular Medicine, University of South Florida, 12901 Bruce B. Downs Blvd. MDC07, Tampa, Florida 33612, USA; Phone: 1-813-974-5816; Fax: 1-813-974-7357; E-mail: vuversky@usf.edu

Supplemetary Table S1: Amino acid sequences of α-, β-, and γ-synucleins analyzed in this study

Species	Amino acid sequence (FASTA format)
	α-synuclein
Homo sapiens	>sp|P37840|SYUA_HUMAN Alpha-synuclein OS=Homo sapiens OX=9606 GN=SNCA PE=1 SV=1<br>MDVFMKGLSKAKEGVVAAAEKTKQGVAEAAGKTKEGVLYVGSKTKEGVVHGVATVAEKTKEQVTN<br>VGGAVVTGVTAVAQKTVEGAGSIAAATGFVKKDQLGKNEEGAPQEGILEDMPVDPDNEAYEMPSE<br>EGYQDYEPEA
Macaca fascicularis	>tr|G7P5X2|G7P5X2_MACFA Alpha-synuclein OS=Macaca fascicularis OX=9541 GN=EGM_14544 PE=3 SV=1<br>MDVFMKGLSKAKEGVVAAAEKTKQGVAEAAGKTKEGVLYVGSKTKEGVVHGVATVAEKTKEQV<br>TNVGGAVVTGVTAVAQKTVEGAGSIAAATGFIKKDQLGKNEEGAPQEGILQDMPVDPDNEAYE<br>MPSEEGYQDYEPEA
Mus musculus	>sp|O55042|SYUA_MOUSE Alpha-synuclein OS=Mus musculus OX=10090 GN=Snca PE=1 SV=2<br>MDVFMKGLSKAKEGVVAAAEKTKQGVAEAAGKTKEGVLYVGSKTKEGVVHGVTTVAEKTKEQV<br>TNVGGAVVTGVTAVAQKTVEGAGNIAAATGFVKKDQMGKGEEGYPQEGILEDMPVDPGSEAYE<br>MPSEEGYQDYEPEA
Monodelphis domestica	Sequence is not available

<table>
<tr><td>Tachyglossus aculeatus</td><td>Sequence is not available</td></tr>
<tr><td>Gallus gallus</td><td>>tr|Q9I9H1|Q9I9H1_CHICK Alpha-synuclein OS=Gallus gallus OX=9031 GN=SNCA PE=1 SV=1

MDVFMKGLNKAKEGVVAAAEKTKQGVAEAAGKTKEGVLYVGSRTKEGVVHGVTTVAEKTKEQV
SNVGGAVVTGVTAVAQKTVEGAGNIAAATGLVKKDQLAKQNEEGFLQEGMVNNTDIPVDPENE
AYEMPPEEEYQDYEPEA</td></tr>
<tr><td>Pelodiscus sinensis</td><td>>tr|K7FW98|K7FW98_PELSI Alpha-synuclein OS=Pelodiscus sinensis OX=13735 GN=SNCA PE=3 SV=1

MDVFMKGLSKAKEGVVAAAEKTKQGMAEAAGKTKEGVLYVGSRTREGVVHGVTTMAEKTKEQV
SNVGGAVVTGVTAVAHKTVEGAGNIAAATGLVKKDQMAKQNEEGLSQEGMMDSTDMPMDPDNE
AYEMPPEEEYQDYEPEA</td></tr>
<tr><td>Xenopus laevis</td><td>>tr|Q7SZ02|Q7SZ02_XENLA Alpha-synuclein OS=Xenopus laevis OX=8355 GN=snca.L PE=2 SV=1

MDVFMKGLSKAKEGVVAAAEKTKQGVAEAAGKTKEGVLYVGSKTKEGVVHGVTTVAEKTKEQV
SNVGGAVVTGVTAVAHKTVEGAGNFAAATGLVKKDQKNESGFGPEGTMENSENMPVNPNNETY
EMPPEEEYQDYDPEA</td></tr>
<tr><td>Erpetoichthys calabaricus</td><td>>tr|A0A8C4RPQ9|A0A8C4RPQ9_ERPCA Alpha-synuclein OS=Erpetoichthys calabaricus OX=27687 GN=SNCA PE=3 SV=1

MDVLMKGLSKAKEGVVAAAEKTKQGVAEAAGKTKEGVMYVGTKTKDGVTSVAEKTKEQVSNVG
GAVVTGVTAVAHKTVEGAGNIAAATGLVKRDHLGKQNEDMLSQEGLMDNTADYPLDPDDTYDM
PPEDDCQEYQPNA</td></tr>
<tr><td colspan="2">β-synuclein</td></tr>
<tr><td>Homo sapiens</td><td>>sp|Q16143|SYUB_HUMAN Beta-synuclein OS=Homo sapiens OX=9606 GN=SNCB PE=1 SV=1

MDVFMKGLSMAKEGVVAAAEKTKQGVTEAAEKTKEGVLYVGSKTREGVVQGVASVAEKTKEQAS
HLGGAVFSGAGNIAAATGLVKREEFPTDLKPEEVAQEAAEEPLIEPLMEPEGESYEDPPQEEYQ
EYEPEA</td></tr>
<tr><td>Macaca fascicularis</td><td>>tr|A0A7N9I9A4|A0A7N9I9A4_MACFA Beta-synuclein OS=Macaca fascicularis OX=9541 GN=SNCB PE=3 SV=1

GPATQAPDPGPIPTPAPSPPRPGLAEPPRPPAAATVPSPAPAPHPGSKTREGVVQGVASVAEK
TKEQASHLGGAVFSGAGNIAAATGLVKREEFPTDLKPEEVAQEAAEEPLIEPLMEPEGESYED
PPQEEYQEYEPEA</td></tr>
<tr><td>Mus musculus</td><td>>sp|Q91ZZ3|SYUB_MOUSE Beta-synuclein OS=Mus musculus OX=10090 GN=Sncb PE=1 SV=1

MDVFMKGLSMAKEGVVAAAEKTKQGVTEAAEKTKEGVLYVGSKTSGVVQGVASVAEKTKEQAS
HLGGAVFSGAGNIAAATGLVKKEEFPTDLKPEEVAQEAAEEPLIEPLMEPEGESYEDSPQEEY
QEYEPEA</td></tr>
</table>

Monodelphis domestica	>tr|F6T1V6|F6T1V6_MONDO Beta-synuclein OS=Monodelphis domestica OX=13616 GN=SNCB PE=3 SV=1<br>MDMFMKGLSMAKEGVVAAAEKTKQGVTEAAEKTKEGVLYVGSEIRSKDRARGSGSVVRDQSNE STEGITMGGIRGDDGSSVGVGRVLVKSSQWWHGIPEEVGQEVGEEPMAEPLLDTEGESYEEPP QEEYQEYEPEA
Tachyglossus aculeatus	>XP_038626735.1 beta-synuclein [Tachyglossus aculeatus]<br>MDMFMKGLNMAKEGVVAAAEKTKQGVTEAAEKTKEGVLYVGNRTREGVVQSVASVAEKTKEQA SQLGGAVFSGAGNIAAATGLMKKEDLPADVKPEEVGQEAGEEPLIEPLLEPEGESYEDPTQEE YQEYEPEA
Gallus gallus	>NP_990002.1 beta-synuclein [Gallus gallus]<br>MEVFMKGLSKAKEGVVAAAEKTKQGVAEAAEKTKEGVLYVGSKTQGVVQGVTSVAEKAKEQAS QLGEAAFSGAGNIAAATGLVKKEEFPADLKAEEVAQEAVEEPLVEPLLEPEGESYEESPQEEY QEYEPEA
Pelodiscus sinensis	>tr|K7FSI4|K7FSI4_PELSI Beta-synuclein-like OS=Pelodiscus sinensis OX=13735 GN=SNCB PE=3 SV=1<br>VAEKTKEQASQLGGAVMSGAGNIAAATGLVKKEEFPTDLKPEEVGQEAGEEPLSEPLLEPEGE AYEEPPQ
Xenopus laevis	>tr|Q6GQG2|Q6GQG2_XENLA Beta-synuclein OS=Xenopus laevis OX=8355 GN=sncb.S PE=2 SV=1<br>MDVFMKGFSKAKEGVVAAAEKTKQGVAEAAEKTKEGVLYVGNKTRDGVVQGVTSVAEKTKEQA SHLGGAVMSGAGNIAAATGLVKKDEFPTDLKPEEEAQEALEEPAAEPLLEPEGESYEDPQDDY QEYEPEA
Erpetoichthys calabaricus	>tr|A0A8C4TNP5|A0A8C4TNP5_ERPCA Beta-synuclein OS=Erpetoichthys calabaricus OX=27687 GN=sncb PE=3 SV=1<br>MDVLMKGLSKAKEGMAAAAEKTKEGVAVAAEKTKEGVLYVGNMTKEGVVQGVASVAEKTKEQA SQLGGAVFSGAGNIAAATGLMKKEEFPTDIKPEELGQEAVEEPLGEPIMEPEGEAYEEAPQDE YQEYEPEA
γ-synuclein	
Homo sapiens	>sp|O76070|SYUG_HUMAN Gamma-synuclein OS=Homo sapiens OX=9606 GN=SNCG PE=1 SV=2<br>MDVFKKGFSIAKEGVVGAVEKTKQGVTEAAEKTKEGVMYVGAKTKENVVQSVTSVAEKTKEQANA VSEAVVSSVNTVATKTVEEAENIAVTSGVVRKEDLRPSAPQQEGEASKEKEEVAEEAQSGGD
Macaca fascicularis	>tr|G7PEV7|G7PEV7_MACFA Gamma-synuclein OS=Macaca fascicularis OX=9541 GN=EGM_17995 PE=3 SV=1<br>MDVFKKGFSIAKEGVVGAVEKTKQGVTEAAEKTKEGVMYVGTKTKENVVHSVTSVAEKTKEQA NAVSEAVVSSVNTVAAKTVEEAENIAVTSGVVRKEDLKPSAPQQEGEAAKEKEEVAEEAQSGG D

<table>
<tr><td>Mus musculus</td><td>>sp|Q9Z0F7|SYUG_MOUSE Gamma-synuclein OS=Mus musculus OX=10090 GN=Sncg PE=1 SV=1

MDVFKKGFSIAKEGVVGAVEKTKQGVTEAAEKTKEGVMYVGTKTKENVVQSVTSVAEKTKEQA
NAVSEAVVSSVNTVANKTVEEAENIVVTTGVVRKEDLEPPAQDQEAKEQEENEEAKSGED</td></tr>
<tr><td>Monodelphis domestica</td><td>>tr|A0A5F8G4U8|A0A5F8G4U8_MONDO Gamma-synuclein OS=Monodelphis domestica OX=13616 GN=SNCG PE=3 SV=1

MDVFKKGFSIAKEGVVGAVEKTKQGVTEAAEKTKEGVMYVGTKTKEGVVQSVTSVAEKTKEQA
NLVSDVMVASVNTVANKTVEEAENIVVTSGIVRKEDLVNPAQPEHGAPEEQPAEAAEVTEEVG
APDAFFPLSPGLDRFSLFSPLLPASWASVTAAIRGAEEKGFPPCGLFSLLLSPFILP</td></tr>
<tr><td>Tachyglossus aculeatus</td><td>>XP_038599981.1 gamma-synuclein [Tachyglossus aculeatus]

MDVFKKGFSIAMDGVVAAAEKTKQGVTEAAEKTKEGVMYVGTKTKEGVVQSVSSVAEKTKEQA
SAVSEAMVASVNTVASKTVEGAESIVVTAGVVKKEDLLRPDQLEEAAAEENPAEAPAEVPEAT
EKEDNGGN</td></tr>
<tr><td>Gallus gallus</td><td>>tr|Q9I9H0|Q9I9H0_CHICK Gamma-synuclein OS=Gallus gallus OX=9031 GN=SNCG1 PE=1 SV=1

MDVFKKGFSIAKEGVVAAAEKTKQGVTEAAEKTKEGVMYVGTKTKEGVVQSVTSVAEKTKEQA
NVVGEAVVASVNTVANKTVEGAETIVATTGVVKKEDLAPQQPAAEGEAAIPGSTEGGGEGENE
GN</td></tr>
<tr><td>Pelodiscus sinensis</td><td>>tr|K7FIG2|K7FIG2_PELSI Gamma-synuclein OS=Pelodiscus sinensis OX=13735 GN=SNCG PE=3 SV=1

MDVFKKGFSIAKEGVVAAAEKTKQGVTEAAEKTKEGVMYVGAKTKEGVVHSVSSVAEKTKEQA
NMVGEAVVASVNTVAGKTVEGAENIVTTTGIVKKEELSHPEHPAEPAAAEEEPPAEDIKATGE
GESESN</td></tr>
<tr><td>Xenopus laevis</td><td>>tr|Q7SYT8|Q7SYT8_XENLA Gamma-synuclein OS=Xenopus laevis OX=8355 GN=sncg.S PE=2 SV=1

MDVFKKGFSMAKEGVVAAAEKTKQGVTEAAEKTKEGVMYVGAKTKEGVVHSVNTVAEKTKEQA
NVVGGAVVSGVNQVSSKTVEGTENVVSSTGLVKKEDLHPDQPEEPAAEEPAVEATESIEQVGD
GEN</td></tr>
<tr><td>Erpetoichthys calabaricus</td><td>>tr|A0A8C4SHF3|A0A8C4SHF3_ERPCA Gamma-synuclein OS=Erpetoichthys calabaricus OX=27687 GN=sncg PE=3 SV=1

MDVFKKGFSMAKEGVVAAAEKTKHGVEEAAAKTKEGVMYVGTKTKEGVAHSVNAVAEKTKEQA
NIVGDAVVSSATQVSAKTVEGVENVAATGGLIKKDEQEGELVQNPAENESEEDAQAAEQVGN</td></tr>
</table>

Supplementary Table S2. Functional enrichment of the STRING-generated PPI network centerd at human α-synuclein. Enrichment is presented in terms of the abundance of proteins in various KEEG pathways.

Pathway	Description	Count	Strength	FDR
hsa04520	Adherens junction	7 of 69	0.75	0.0013
hsa04261	Adrenergic signaling in cardiomyocytes	11 of 146	0.62	0.00046
hsa05143	African trypanosomiasis	5 of 36	0.88	0.0022
hsa04933	AGE-RAGE signaling pathway in diabetic complications	10 of 96	0.76	9.33e-05
hsa05034	Alcoholism	21 of 146	0.9	4.45e-11
hsa04925	Aldosterone synthesis and secretion	9 of 94	0.72	0.00037
hsa04960	Aldosterone-regulated sodium reabsorption	4 of 37	0.78	0.0143
hsa05010	Alzheimer disease	114 of 354	1.25	3.13e-97
hsa05146	Amoebiasis	10 of 101	0.74	0.00013
hsa05031	Amphetamine addiction	16 of 65	1.13	1.69e-11
hsa04152	AMPK signaling pathway	9 of 120	0.62	0.0016
hsa05014	Amyotrophic lateral sclerosis	115 of 350	1.26	8.07e-99
hsa01523	Antifolate resistance	3 of 31	0.73	0.0483
hsa04612	Antigen processing and presentation	11 of 64	0.98	7.25e-07
hsa04371	Apelin signaling pathway	15 of 133	0.79	5.97e-07
hsa04210	Apoptosis	16 of 131	0.83	9.28e-08
hsa04215	Apoptosis - multiple species	8 of 30	1.17	2.23e-06
hsa04140	Autophagy - animal	18 of 131	0.88	2.38e-09
hsa04136	Autophagy - other	4 of 31	0.85	0.0084
hsa05219	Bladder cancer	5 of 40	0.84	0.0033
hsa05224	Breast cancer	8 of 146	0.48	0.0151
hsa04625	C-type lectin receptor signaling pathway	12 of 101	0.82	5.69e-06
hsa04024	cAMP signaling pathway	14 of 207	0.57	0.00019
hsa04218	Cellular senescence	13 of 150	0.68	4.01e-05
hsa05230	Central carbon metabolism in cancer	5 of 68	0.61	0.0216
hsa04022	cGMP-PKG signaling pathway	13 of 163	0.64	8.49e-05
hsa05142	Chagas disease	10 of 97	0.76	9.99e-05
hsa04062	Chemokine signaling pathway	8 of 186	0.38	0.0493
hsa04979	Cholesterol metabolism	6 of 48	0.84	0.0013
hsa04725	Cholinergic synapse	8 of 109	0.61	0.0033
hsa05220	Chronic myeloid leukemia	7 of 75	0.71	0.0020
hsa04713	Circadian entrainment	10 of 91	0.78	6.65e-05
hsa05030	Cocaine addiction	12 of 49	1.13	8.04e-09
hsa05210	Colorectal cancer	10 of 82	0.83	3.17e-05
hsa04728	Dopaminergic synapse	24 of 126	1.02	6.21e-15
hsa01521	EGFR tyrosine kinase inhibitor resistance	10 of 77	0.86	2.04e-05
hsa01522	Endocrine resistance	8 of 94	0.67	0.0015
hsa04144	Endocytosis	17 of 241	0.59	2.48e-05
hsa05213	Endometrial cancer	7 of 58	0.82	0.00057
hsa05169	Epstein-Barr virus infection	28 of 192	0.91	8.24e-15
hsa04012	ErbB signaling pathway	7 of 81	0.68	0.0028
hsa04915	Estrogen signaling pathway	19 of 133	0.9	4.58e-10

hsa04664	Fc epsilon RI signaling pathway	5 of 65	0.63	0.0187
hsa04666	Fc gamma R-mediated phagocytosis	7 of 90	0.63	0.0048
hsa04216	Ferroptosis	6 of 41	0.91	0.00065
hsa05418	Fluid shear stress and atherosclerosis	18 of 129	0.89	1.98e-09
hsa04510	Focal adhesion	10 of 195	0.45	0.0096
hsa04068	FoxO signaling pathway	9 of 126	0.6	0.0021
hsa04540	Gap junction	8 of 87	0.71	0.0010
hsa04971	Gastric acid secretion	8 of 71	0.79	0.00032
hsa05226	Gastric cancer	9 of 146	0.53	0.0051
hsa05214	Glioma	11 of 71	0.93	1.77e-06
hsa04922	Glucagon signaling pathway	10 of 100	0.74	0.00012
hsa04724	Glutamatergic synapse	6 of 112	0.47	0.0418
hsa04929	GnRH secretion	7 of 63	0.79	0.00087
hsa04912	GnRH signaling pathway	11 of 87	0.84	9.21e-06
hsa04935	Growth hormone synthesis, secretion and action	6 of 117	0.45	0.0489
hsa04340	Hedgehog signaling pathway	5 of 46	0.78	0.0056
hsa04640	Hematopoietic cell lineage	6 of 90	0.57	0.0170
hsa05161	Hepatitis B	17 of 158	0.77	1.68e-07
hsa05160	Hepatitis C	15 of 157	0.72	3.53e-06
hsa05225	Hepatocellular carcinoma	11 of 161	0.58	0.00095
hsa04066	HIF-1 signaling pathway	9 of 102	0.69	0.00061
hsa05163	Human cytomegalovirus infection	21 of 217	0.73	2.16e-08
hsa05170	Human immunodeficiency virus 1 infection	19 of 203	0.71	1.82e-07
hsa05165	Human papillomavirus infection	14 of 324	0.38	0.0080
hsa05166	Human T-cell leukemia virus 1 infection	14 of 210	0.57	0.00022
hsa05016	Huntington disease	101 of 295	1.28	1.20e-87
hsa04657	IL-17 signaling pathway	12 of 91	0.86	2.30e-06
hsa05321	Inflammatory bowel disease	5 of 59	0.67	0.0136
hsa04750	Inflammatory mediator regulation of TRP channels	10 of 92	0.78	7.15e-05
hsa05164	Influenza A	13 of 163	0.64	8.49e-05
hsa04931	Insulin resistance	8 of 106	0.62	0.0028
hsa04911	Insulin secretion	6 of 82	0.61	0.0119
hsa04910	Insulin signaling pathway	11 of 132	0.66	0.00022
hsa05167	Kaposi sarcoma-associated herpesvirus infection	22 of 187	0.81	4.13e-10
hsa05134	Legionellosis	13 of 55	1.12	2.43e-09
hsa05140	Leishmaniasis	8 of 69	0.81	0.00027
hsa04720	Long-term potentiation	9 of 63	0.9	2.98e-05
hsa04211	Longevity regulating pathway	8 of 87	0.71	0.0010
hsa04213	Longevity regulating pathway - multiple species	8 of 61	0.86	0.00013
hsa05144	Malaria	7 of 46	0.92	0.00018
hsa04010	MAPK signaling pathway	20 of 286	0.59	4.65e-06
hsa05162	Measles	16 of 137	0.81	1.60e-07
hsa04916	Melanogenesis	10 of 95	0.76	8.71e-05
hsa05218	Melanoma	7 of 72	0.73	0.0016
hsa01100	Metabolic pathways	61 of 1435	0.37	9.91e-09
hsa05206	MicroRNAs in cancer	9 of 159	0.49	0.0082

hsa04137	Mitophagy - animal	15 of 64	1.11	1.45e-10
hsa04150	mTOR signaling pathway	7 of 150	0.41	0.0486
hsa04217	Necroptosis	10 of 147	0.57	0.0016
hsa04722	Neurotrophin signaling pathway	20 of 112	0.99	5.11e-12
hsa04064	NF-kappa B signaling pathway	7 of 101	0.58	0.0082
hsa04621	NOD-like receptor signaling pathway	15 of 173	0.68	9.67e-06
hsa04932	Non-alcoholic fatty liver disease	47 of 146	1.25	3.01e-38
hsa05223	Non-small cell lung cancer	6 of 68	0.69	0.0055
hsa04114	Oocyte meiosis	11 of 121	0.7	0.00011
hsa04380	Osteoclast differentiation	10 of 120	0.66	0.00044
hsa00190	Oxidative phosphorylation	45 of 128	1.29	5.04e-38
hsa04921	Oxytocin signaling pathway	11 of 147	0.62	0.00048
hsa04115	p53 signaling pathway	7 of 72	0.73	0.0016
hsa05212	Pancreatic cancer	8 of 71	0.79	0.00032
hsa04928	Parathyroid hormone synthesis, secretion and action	11 of 104	0.77	3.66e-05
hsa05012	Parkinson disease	122 of 236	1.46	1.60e-124
hsa05130	Pathogenic Escherichia coli infection	18 of 187	0.73	2.83e-07
hsa05200	Pathways in cancer	27 of 515	0.46	1.08e-05
hsa05235	PD-L1 expression and PD-1 checkpoint pathway in cancer	9 of 87	0.76	0.00022
hsa05133	Pertussis	14 of 73	1.02	5.17e-09
hsa04145	Phagosome	10 of 141	0.59	0.0013
hsa00360	Phenylalanine metabolism	3 of 17	0.99	0.0124
hsa04070	Phosphatidylinositol signaling system	7 of 94	0.61	0.0058
hsa04072	Phospholipase D signaling pathway	8 of 147	0.48	0.0155
hsa04744	Phototransduction	5 of 26	1.03	0.00073
hsa04151	PI3K-Akt signaling pathway	24 of 349	0.58	5.97e-07
hsa04611	Platelet activation	7 of 122	0.5	0.0190
hsa01524	Platinum drug resistance	9 of 70	0.85	5.85e-05
hsa05020	Prion disease	104 of 263	1.34	1.13e-95
hsa04914	Progesterone-mediated oocyte maturation	6 of 95	0.54	0.0212
hsa04917	Prolactin signaling pathway	7 of 68	0.75	0.0013
hsa05215	Prostate cancer	12 of 97	0.83	4.09e-06
hsa03050	Proteasome	32 of 43	1.61	7.43e-35
hsa04141	Protein processing in endoplasmic reticulum	21 of 163	0.85	2.65e-10
hsa05205	Proteoglycans in cancer	14 of 194	0.6	0.00011
hsa04015	Rap1 signaling pathway	18 of 201	0.69	7.19e-07
hsa04014	Ras signaling pathway	20 of 225	0.69	1.75e-07
hsa04810	Regulation of actin cytoskeleton	10 of 209	0.42	0.0143
hsa04926	Relaxin signaling pathway	9 of 126	0.6	0.0021
hsa04924	Renin secretion	7 of 66	0.77	0.0011
hsa04723	Retrograde endocannabinoid signaling	48 of 142	1.27	7.48e-40
hsa05323	Rheumatoid arthritis	5 of 83	0.52	0.0447
hsa04970	Salivary secretion	8 of 89	0.7	0.0012
hsa05132	Salmonella infection	23 of 209	0.78	4.58e-10
hsa04726	Serotonergic synapse	11 of 108	0.75	4.93e-05
hsa05131	Shigellosis	26 of 218	0.82	6.02e-12

hsa05222	Small cell lung cancer	8 of 92	0.68	0.0014
hsa04071	Sphingolipid signaling pathway	11 of 116	0.72	8.49e-05
hsa05017	Spinocerebellar ataxia	43 of 135	1.24	7.43e-35
hsa04721	Synaptic vesicle cycle	10 of 72	0.88	1.22e-05
hsa04660	T cell receptor signaling pathway	7 of 100	0.59	0.0079
hsa04659	Th17 cell differentiation	7 of 99	0.59	0.0076
hsa04714	Thermogenesis	48 of 226	1.07	4.03e-32
hsa05216	Thyroid cancer	4 of 37	0.78	0.0143
hsa04919	Thyroid hormone signaling pathway	8 of 120	0.57	0.0056
hsa04918	Thyroid hormone synthesis	7 of 73	0.72	0.0017
hsa04530	Tight junction	8 of 157	0.45	0.0214
hsa04668	TNF signaling pathway	8 of 111	0.6	0.0037
hsa04620	Toll-like receptor signaling pathway	11 of 100	0.78	2.78e-05
hsa05145	Toxoplasmosis	13 of 103	0.84	1.25e-06
hsa05152	Tuberculosis	27 of 165	0.96	2.55e-15
hsa04940	Type I diabetes mellitus	4 of 38	0.76	0.0151
hsa04930	Type II diabetes mellitus	4 of 45	0.69	0.0246
hsa00350	Tyrosine metabolism	5 of 35	0.9	0.0021
hsa04120	Ubiquitin mediated proteolysis	13 of 134	0.73	1.47e-05
hsa04270	Vascular smooth muscle contraction	9 of 132	0.58	0.0028
hsa04370	VEGF signaling pathway	5 of 56	0.69	0.0113
hsa05203	Viral carcinogenesis	16 of 183	0.68	4.23e-06
hsa05416	Viral myocarditis	5 of 55	0.7	0.0106
hsa05135	Yersinia infection	12 of 124	0.73	3.31e-05

Count: The first number indicates how many proteins in the network are annotated with a particular term. The second number indicates how many proteins in total (in the generated network and in the background) have this term assigned.
Strength: Log_{10}(observed / expected). This measure describes how large the enrichment effect is. It's the ratio between i) the number of proteins in the generated network that are annotated with a term and ii) the number of proteins that are expected to be annotated with this term in a random network of the same size.
FDR (False Discovery Rate): This measure describes how significant the enrichment is. Shown are p-values corrected for multiple testing within each category using the Benjamini–Hochberg procedure.

Supplementary Figure S1. **CLUSTAL O(1.2.4) multiple sequence alignment of alpha-synucleins**

```
tr|A0A8C4RPQ9|A0A8C4RPQ9_ERPCA      MDVLMKGLSKAKEGVVAAAEKTKQGVAEAAGKTKEGVMYVGTKTKDG----VTSVAEKTK 56
tr|K7FW98|K7FW98_PELSI              MDVFMKGLSKAKEGVVAAAEKTKQGMAEAAGKTKEGVLYVGSRTREGVVHGVTTMAEKTK 60
tr|Q7SZ02|Q7SZ02_XENLA              MDVFMKGLSKAKEGVVAAAEKTKQGVAEAAGKTKEGVLYVGSKTKEGVVHGVTTVAEKTK 60
sp|O55042|SYUA_MOUSE                MDVFMKGLSKAKEGVVAAAEKTKQGVAEAAGKTKEGVLYVGSKTKEGVVHGVTTVAEKTK 60
tr|G7P5X2|G7P5X2_MACFA              MDVFMKGLSKAKEGVVAAAEKTKQGVAEAAGKTKEGVLYVGSKTKEGVVHGVATVAEKTK 60
sp|P37840|SYUA_HUMAN                MDVFMKGLSKAKEGVVAAAEKTKQGVAEAAGKTKEGVLYVGSKTKEGVVHGVATVAEKTK 60
tr|Q9I9H1|Q9I9H1_CHICK              MDVFMKGLNKAKEGVVAAAEKTKQGVAEAAGKTKEGVLYVGSRTKEGVVHGVTTVAEKTK 60
                                    ***:****.***************:***********:***::*::*    *:::*****

tr|A0A8C4RPQ9|A0A8C4RPQ9_ERPCA      EQVSNVGGAVVTGVTAVAHKTVEGAGNIAAATGLVKRDHLGKQNEDMLSQEGLMDNTADY 116
tr|K7FW98|K7FW98_PELSI              EQVSNVGGAVVTGVTAVAHKTVEGAGNIAAATGLVKKDQMAKQNEEGLSQEG-MMDSTDM 119
tr|Q7SZ02|Q7SZ02_XENLA              EQVSNVGGAVVTGVTAVAHKTVEGAGNFAAATGLVKKDQK---NESGFGPEGTMENSENM 117
sp|O55042|SYUA_MOUSE                EQVTNVGGAVVTGVTAVAQKTVEGAGNIAAATGFVKKDQMGKG-EEGYPQEG-IL--EDM 116
tr|G7P5X2|G7P5X2_MACFA              EQVTNVGGAVVTGVTAVAQKTVEGAGSIAAATGFIKKDQLGKN-EEGAPQEG-IL--QDM 116
sp|P37840|SYUA_HUMAN                EQVTNVGGAVVTGVTAVAQKTVEGAGSIAAATGFVKKDQLGKN-EEGAPQEG-IL--EDM 116
tr|Q9I9H1|Q9I9H1_CHICK              EQVSNVGGAVVTGVTAVAQKTVEGAGNIAAATGLVKKDQLAKQNEEGFLQEG-MVNNTDI 119
                                    ***:*************:*******.:*****::*:*:     *.    ** :    :

tr|A0A8C4RPQ9|A0A8C4RPQ9_ERPCA      PLDPD-DTYDMPPEDDCQEYQPNA        139
tr|K7FW98|K7FW98_PELSI              PMDPDNEAYEMPPEEEYQDYEPEA        143
tr|Q7SZ02|Q7SZ02_XENLA              PVNPNNETYEMPPEEEYQDYDPEA        141
sp|O55042|SYUA_MOUSE                PVDPGSEAYEMPSEEGYQDYEPEA        140
tr|G7P5X2|G7P5X2_MACFA              PVDPDNEAYEMPSEEGYQDYEPEA        140
sp|P37840|SYUA_HUMAN                PVDPDNEAYEMPSEEGYQDYEPEA        140
tr|Q9I9H1|Q9I9H1_CHICK              PVDPENEAYEMPPEEEYQDYEPEA        143
                                    *::*  ::*:** *:  *:*:*:*
```

```
#  Percent Identity  Matrix - created by Clustal2.1
#
     1: tr|A0A8C4RPQ9|A0A8C4RPQ9_ERPCA  100.00   78.26   77.21   76.30   75.56   76.30   78.26
     2: tr|K7FW98|K7FW98_PELSI           78.26  100.00   86.43   86.43   85.00   85.71   90.21
     3: tr|Q7SZ02|Q7SZ02_XENLA           77.21   86.43  100.00   86.13   83.94   85.40   87.86
     4: sp|O55042|SYUA_MOUSE             76.30   86.43   86.13  100.00   93.57   95.00   87.86
     5: tr|G7P5X2|G7P5X2_MACFA           75.56   85.00   83.94   93.57  100.00   98.57   87.14
     6: sp|P37840|SYUA_HUMAN             76.30   85.71   85.40   95.00   98.57  100.00   87.86
     7: tr|Q9I9H1|Q9I9H1_CHICK           78.26   90.21   87.86   87.86   87.14   87.86  100.00
```

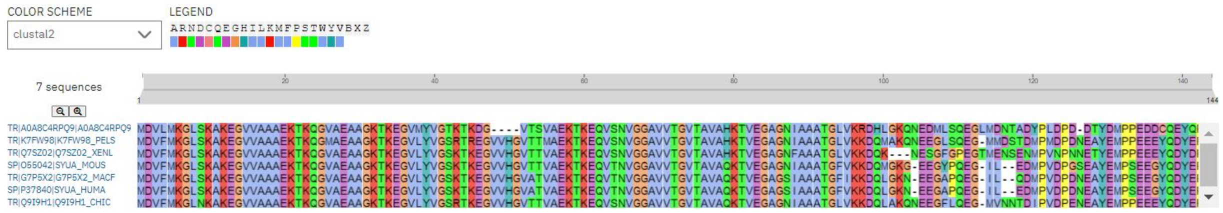

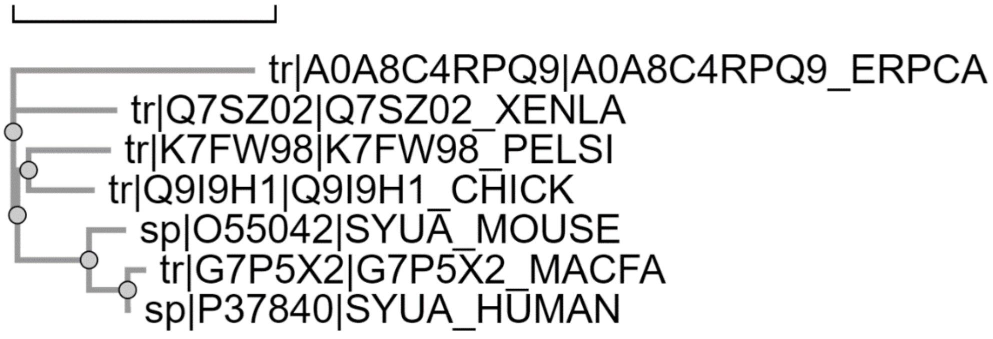

Supplementary Figure S2. CLUSTAL O(1.2.4) multiple sequence alignment of beta-synucleins

```
tr|F6T1V6|F6T1V6_MONDO              MDMFMKGLSMAK--EGVVA--AAEKTKQGVTE-------AAEKTKEGVLYVGSEIRSKDR       49
NP_990002.1                         MEVFMKGLSKAK--EGVVA--AAEKTKQGVAE-------AAEKTKEGVLYVGSKT-QGVV       48
tr|Q6GQG2|Q6GQG2_XENLA              MDVFMKGFSKAK--EGVVA--AAEKTKQGVAE-------AAEKTKEGVLYVGNKTRDGVV       49
XP_038626735.1                      MDMFMKGLNMAK--EGVVA--AAEKTKQGVTE-------AAEKTKEGVLYVGNRTREGVV       49
tr|A0A8C4TNP5|A0A8C4TNP5_ERPCA      MDVLMKGLSKAK--EGMAA--AAEKTKEGV-------AVAAEKTKEGVLYVGNMTKEGVV       49
tr|A0A7N9I9A4|A0A7N9I9A4_MACFA      ------GPATQAPDPGPIPTPAPSPPRPGLAEPPRPPAAATVPSPAPAPHPGSKTREGVV       54
sp|Q16143|SYUB_HUMAN                MDVFMKGLSM--AKEGVVA--AAEKTKQGVTE-------AAEKTKEGVLYVGSKTREGVV       49
sp|Q91ZZ3|SYUB_MOUSE                MDVFMKGLSM--AKEGVVA--AAEKTKQGVTE-------AAEKTKEGVLYVGSKT-SGVV       48
tr|K7FSI4|K7FSI4_PELSI              ------------------------------------------------------------       0


tr|F6T1V6|F6T1V6_MONDO              ARGSGSVVRDQSNESTEGITMGGIRGDDGSSVGVGRVLVKSSQW-WHGIPEEVGQEVGEE       108
NP_990002.1                         -QGVTSVAEKA-KE--QASQLGEAAFSGAGNIAAATGLVKKEEFPADLKAEEVAQEAVEE       104
tr|Q6GQG2|Q6GQG2_XENLA              -QGVTSVAEKT-KE--QASHLGGAVMSGAGNIAAATGLVKKDEFPTDLKPEEEAQEALEE       105
XP_038626735.1                      -QSVASVAEKT-KE--QASQLGGAVFSGAGNIAAATGLMKKEDLPADVKPEEVGQEAGEE       105
tr|A0A8C4TNP5|A0A8C4TNP5_ERPCA      -QGVASVAEKT-KE--QASQLGGAVFSGAGNIAAATGLMKKEEFPTDIKPEELGQEAVEE       105
tr|A0A7N9I9A4|A0A7N9I9A4_MACFA      -QGVASVAEKT-KE--QASHLGGAVFSGAGNIAAATGLVKREEFPTDLKPEEVAQEAAEE       110
sp|Q16143|SYUB_HUMAN                -QGVASVAEKT-KE--QASHLGGAVFSGAGNIAAATGLVKREEFPTDLKPEEVAQEAAEE       105
sp|Q91ZZ3|SYUB_MOUSE                -QGVASVAEKT-KE--QASHLGGAVFSGAGNIAAATGLVKKEEFPTDLKPEEVAQEAAEE       104
tr|K7FSI4|K7FSI4_PELSI              ------VAEKT-KE--QASQLGGAVMSGAGNIAAATGLVKKEEFPTDLKPEEVGQEAGEE       51
                                          *...  :*  :.  :*    .....:...  *:* .:   .   ** .**. **

tr|F6T1V6|F6T1V6_MONDO              PMAEPLLDTEGESYEEPPQEEYQEYEPEA        137
NP_990002.1                         PLVEPLLEPEGESYEESPQEEYQEYEPEA        133
tr|Q6GQG2|Q6GQG2_XENLA              PAAEPLLEPEGESYEDP-QDDYQEYEPEA        133
XP_038626735.1                      PLIEPLLEPEGESYEDPTQEEYQEYEPEA        134
tr|A0A8C4TNP5|A0A8C4TNP5_ERPCA      PLGEPIMEPEGEAYEEAPQDEYQEYEPEA        134
tr|A0A7N9I9A4|A0A7N9I9A4_MACFA      PLIEPLMEPEGESYEDPPQEEYQEYEPEA        139
sp|Q16143|SYUB_HUMAN                PLIEPLMEPEGESYEDPPQEEYQEYEPEA        134
sp|Q91ZZ3|SYUB_MOUSE                PLIEPLMEPEGESYEDSPQEEYQEYEPEA        133
tr|K7FSI4|K7FSI4_PELSI              PLSEPLLEPEGEAYEEPPQ----------        70
                                    *  **::: ***:**:  *
```

Percent Identity Matrix - created by Clustal 2.1

#

1: tr|F6T1V6|F6T1V6_MONDO	100.00	58.33	57.58	60.90	52.67	38.58	61.07	60.77	44.93
2: NP_990002.1	58.33	100.00	84.85	81.95	81.68	67.72	87.02	88.55	84.29
3: tr|Q6GQG2|Q6GQG2_XENLA	57.58	84.85	100.00	81.95	80.92	68.50	87.79	87.69	86.96
4: XP_038626735.1	60.90	81.95	81.95	100.00	81.82	67.19	87.88	87.02	85.71
5: tr|A0A8C4TNP5|A0A8C4TNP5_ERPCA	52.67	81.68	80.92	81.82	100.00	65.62	83.85	84.50	87.14
6: tr|A0A7N9I9A4|A0A7N9I9A4_MACFA	38.58	67.72	68.50	67.19	65.62	100.00	78.12	75.59	87.14
7: sp|Q16143|SYUB_HUMAN	61.07	87.02	87.79	87.88	83.85	78.12	100.00	97.74	87.14
8: sp|Q91ZZ3|SYUB_MOUSE	60.77	88.55	87.69	87.02	84.50	75.59	97.74	100.00	87.14
9: tr|K7FSI4|K7FSI4_PELSI	44.93	84.29	86.96	85.71	87.14	87.14	87.14	87.14	100.00

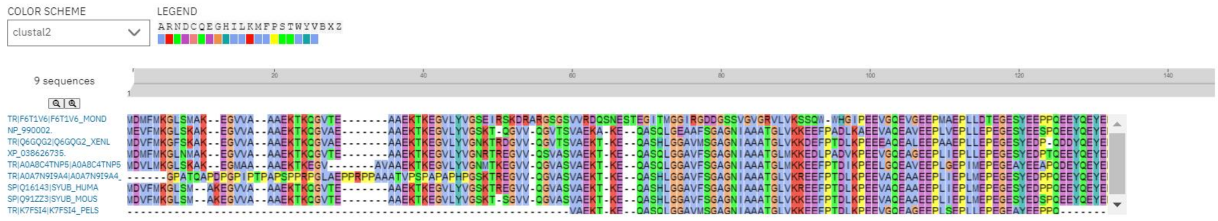

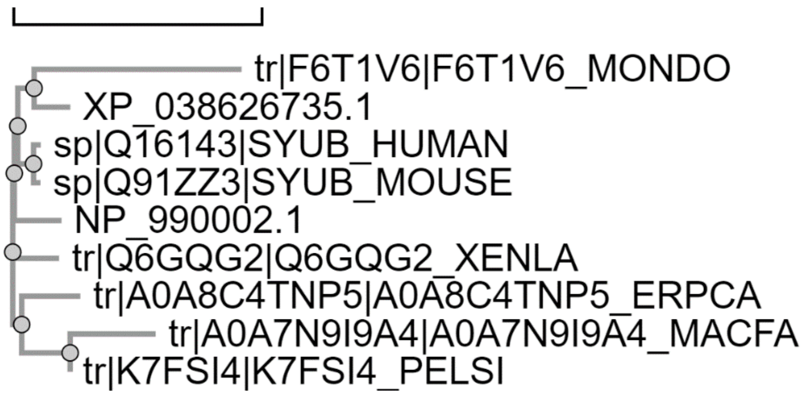
tr|F6T1V6|F6T1V6_MONDO
XP_038626735.1
sp|Q16143|SYUB_HUMAN
sp|Q91ZZ3|SYUB_MOUSE
NP_990002.1
tr|Q6GQG2|Q6GQG2_XENLA
tr|A0A8C4TNP5|A0A8C4TNP5_ERPCA
tr|A0A7N9I9A4|A0A7N9I9A4_MACFA
tr|K7FSI4|K7FSI4_PELSI

Supplementary Figure S3. **CLUSTAL O(1.2.4) multiple sequence alignment of gamma-synucleins**

```
tr|A0A8C4SHF3|A0A8C4SHF3_ERPCA      MDVFKKGFSMAKEGVVAAAEKTKHGVEEAAAKTKEGVMYVGTKTKEGVAHSVNAVAEKTK  60
tr|Q7SYT8|Q7SYT8_XENLA              MDVFKKGFSMAKEGVVAAAEKTKQGVTEAAEKTKEGVMYVGAKTKEGVVHSVNTVAEKTK  60
tr|Q9I9H0|Q9I9H0_CHICK              MDVFKKGFSIAKEGVVAAAEKTKQGVTEAAEKTKEGVMYVGTKTKEGVVQSVTSVAEKTK  60
tr|K7FIG2|K7FIG2_PELSI              MDVFKKGFSIAKEGVVAAAEKTKQGVTEAAEKTKEGVMYVGAKTKEGVVHSVSSVAEKTK  60
XP_038599981.1                      MDVFKKGFSIAMDGVVAAAEKTKQGVTEAAEKTKEGVMYVGTKTKEGVVQSVSSVAEKTK  60
tr|A0A5F8G4U8|A0A5F8G4U8_MONDO      MDVFKKGFSIAKEGVVGAVEKTKQGVTEAAEKTKEGVMYVGTKTKEGVVQSVTSVAEKTK  60
sp|Q9Z0F7|SYUG_MOUSE                MDVFKKGFSIAKEGVVGAVEKTKQGVTEAAEKTKEGVMYVGTKTKENVVQSVTSVAEKTK  60
sp|O76070|SYUG_HUMAN                MDVFKKGFSIAKEGVVGAVEKTKQGVTEAAEKTKEGVMYVGAKTKENVVQSVTSVAEKTK  60
tr|G7PEV7|G7PEV7_MACFA              MDVFKKGFSIAKEGVVGAVEKTKQGVTEAAEKTKEGVMYVGTKTKENVVHSVTSVAEKTK  60
                                    *********:* :***.*.****:** *** **********:****.*.:**.:******

tr|A0A8C4SHF3|A0A8C4SHF3_ERPCA      EQANIVGDAVVSSATQVSAKTVEGVENVAATGGLIKKDEQEGELVQNPAENESEED---A  117
tr|Q7SYT8|Q7SYT8_XENLA              EQANVVGGAVVSGVNQVSSKTVEGTENVVSSTGLVKKEDLHPDQPEE-PAAE-EPAVEAT  118
tr|Q9I9H0|Q9I9H0_CHICK              EQANVVGEAVVASVNTVANKTVEGAETIVATTGVVKKEDLAPQQPAAE----GEAAIP--  114
tr|K7FIG2|K7FIG2_PELSI              EQANMVGEAVVASVNTVAGKTVEGAENIVTTTGIVKKEELSHPEHPAEPAAAEEEPPA--  118
XP_038599981.1                      EQASAVSEAMVASVNTVASKTVEGAESIVVTAGVVKKEDLLRPDQLEEAAAEENPAEAPA  120
tr|A0A5F8G4U8|A0A5F8G4U8_MONDO      EQANLVSDVMVASVNTVANKTVEEAENIVVTSGIVRKEDLVNPAQPEHGAPEEQPAEAAE  120
sp|Q9Z0F7|SYUG_MOUSE                EQANAVSEAVVSSVNTVANKTVEEAENIVVTTGVVRKEDLEPPAQDQEAKEQEENE----  116
sp|O76070|SYUG_HUMAN                EQANAVSEAVVSSVNTVATKTVEEAENIAVTSGVVRKEDLRPSAPQQEGEASKEKEEVAE  120
tr|G7PEV7|G7PEV7_MACFA              EQANAVSEAVVSSVNTVAAKTVEEAENIAVTSGVVRKEDLKPSAPQQEGEAAKEKEEVAE  120
                                    ***. *. .:*:... *: **** .*.:. : *:::*::              :

tr|A0A8C4SHF3|A0A8C4SHF3_ERPCA      QAAEQVGN----------------------------------------------------  125
tr|Q7SYT8|Q7SYT8_XENLA              ESIEQVGDGEN-------------------------------------------------  129
tr|Q9I9H0|Q9I9H0_CHICK              GSTEGGGEGENEGN----------------------------------------------  128
tr|K7FIG2|K7FIG2_PELSI              EDIKATGEGESESN----------------------------------------------  132
XP_038599981.1                      EVPEATEKEDNGGN----------------------------------------------  134
tr|A0A5F8G4U8|A0A5F8G4U8_MONDO      VT-------EEVGAPDAFFPLSPGLDRFSLFSPLLPASWASVTAAIRGAEEKGFPPCGLF  173
sp|Q9Z0F7|SYUG_MOUSE                EA-------KSGED----------------------------------------------  123
sp|O76070|SYUG_HUMAN                EA-------QSGGD----------------------------------------------  127
tr|G7PEV7|G7PEV7_MACFA              EA-------QSGGD----------------------------------------------  127


tr|A0A8C4SHF3|A0A8C4SHF3_ERPCA      ----------       125
tr|Q7SYT8|Q7SYT8_XENLA              ----------       129
tr|Q9I9H0|Q9I9H0_CHICK              ----------       128
tr|K7FIG2|K7FIG2_PELSI              ----------       132
XP_038599981.1                      ----------       134
tr|A0A5F8G4U8|A0A5F8G4U8_MONDO      SLLLSPFILP       183
sp|Q9Z0F7|SYUG_MOUSE                ----------       123
sp|O76070|SYUG_HUMAN                ----------       127
tr|G7PEV7|G7PEV7_MACFA              ----------       127
```

Percent Identity Matrix - created by Clustal 2.1

1: tr|A0A8C4SHF3|A0A8C4SHF3_ERPCA	100.00	69.11	64.17	62.90	57.60	57.98	61.86	61.34	63.87
2: tr|Q7SYT8|Q7SYT8_XENLA	69.11	100.00	76.42	73.23	66.67	67.21	68.64	68.03	68.03
3: tr|Q9I9H0|Q9I9H0_CHICK	64.17	76.42	100.00	78.12	74.22	74.38	78.15	75.21	75.21
4: tr|K7FIG2|K7FIG2_PELSI	62.90	73.23	78.12	100.00	72.73	72.00	73.98	71.20	72.00
5: XP_038599981.1	57.60	66.67	74.22	72.73	100.00	75.59	75.61	70.87	70.87
6: tr|A0A5F8G4U8|A0A5F8G4U8_MONDO	57.98	67.21	74.38	72.00	75.59	100.00	78.05	75.59	75.59
7: sp|Q9Z0F7|SYUG_MOUSE	61.86	68.64	78.15	73.98	75.61	78.05	100.00	86.99	86.99
8: sp|O76070|SYUG_HUMAN	61.34	68.03	75.21	71.20	70.87	75.59	86.99	100.00	96.06
9: tr|G7PEV7|G7PEV7_MACFA	63.87	68.03	75.21	72.00	70.87	75.59	86.99	96.06	100.00

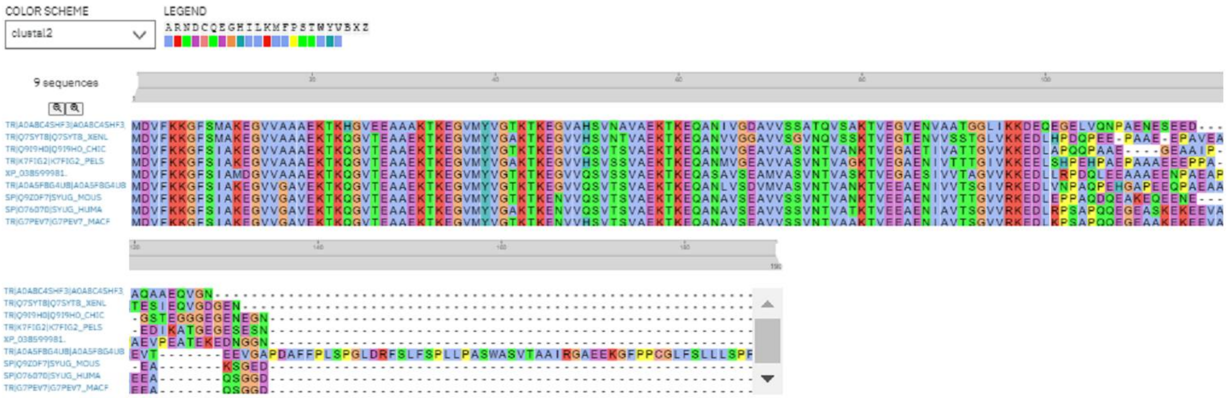
COLOR SCHEME
clustal2
LEGEND
9 sequences

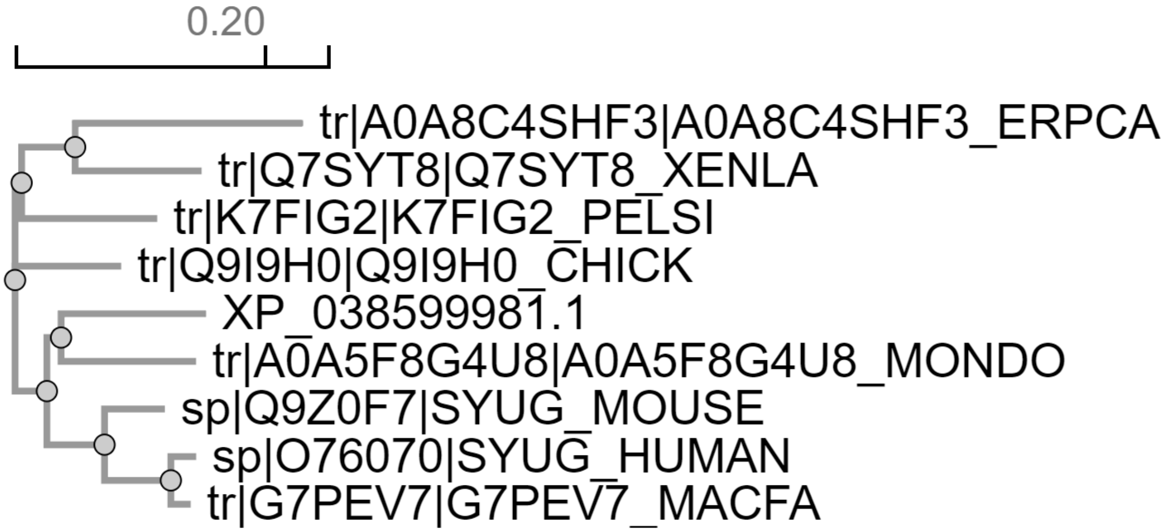
0.20
tr|A0A8C4SHF3|A0A8C4SHF3_ERPCA
tr|Q7SYT8|Q7SYT8_XENLA
tr|K7FIG2|K7FIG2_PELSI
tr|Q9I9H0|Q9I9H0_CHICK
XP_038599981.1
tr|A0A5F8G4U8|A0A5F8G4U8_MONDO
sp|Q9Z0F7|SYUG_MOUSE
sp|O76070|SYUG_HUMAN
tr|G7PEV7|G7PEV7_MACFA

Supplementary Figure S4. CLUSTAL O(1.2.4) multiple sequence alignment of all synucleins

```
tr|A0A8C4SHF3|A0A8C4SHF3_ERPCA      ---------MDVFKKGFSMAKEGVVAAAEKTKHGVEEAAAKTKEGVMYVGTKTKEGVA-H      50
tr|Q7SYT8|Q7SYT8_XENLA              ---------MDVFKKGFSMAKEGVVAAAEKTKQGVTEAAEKTKEGVMYVGAKTKEGVV-H      50
tr|Q9I9H0|Q9I9H0_CHICK              ---------MDVFKKGFSIAKEGVVAAAEKTKQGVTEAAEKTKEGVMYVGTKTKEGVV-Q      50
tr|K7FIG2|K7FIG2_PELSI              ---------MDVFKKGFSIAKEGVVAAAEKTKQGVTEAAEKTKEGVMYVGAKTKEGVV-H      50
XP_038599981.1                      ---------MDVFKKGFSIAMDGVVAAAEKTKQGVTEAAEKTKEGVMYVGTKTKEGVV-Q      50
tr|A0A5F8G4U8|A0A5F8G4U8_MONDO      ---------MDVFKKGFSIAKEGVVGAVEKTKQGVTEAAEKTKEGVMYVGTKTKEGVV-Q      50
sp|Q9Z0F7|SYUG_MOUSE                ---------MDVFKKGFSIAKEGVVGAVEKTKQGVTEAAEKTKEGVMYVGTKTKENVV-Q      50
sp|O76070|SYUG_HUMAN                ---------MDVFKKGFSIAKEGVVGAVEKTKQGVTEAAEKTKEGVMYVGAKTKENVV-Q      50
tr|G7PEV7|G7PEV7_MACFA              ---------MDVFKKGFSIAKEGVVGAVEKTKQGVTEAAEKTKEGVMYVGTKTKENVV-H      50
tr|F6T1V6|F6T1V6_MONDO              ---------MDMFMKGLSMAKEGVVAAAEKTKQGVTEAAEKTKEGVLYVGSEIRSKDRAR      51
tr|Q6GQG2|Q6GQG2_XENLA              ---------MDVFMKGFSKAKEGVVAAAEKTKQGVAEAAEKTKEGVLYVGNKTRDGVV-Q      50
XP_038626735.1                      ---------MDMFMKGLNMAKEGVVAAAEKTKQGVTEAAEKTKEGVLYVGNRTREGVV-Q      50
NP_990002.1                         ---------MEVFMKGLSKAKEGVVAAAEKTKQGVAEAAEKTKEGVLYVGSKT-QGVV-Q      49
sp|Q16143|SYUB_HUMAN                ---------MDVFMKGLSMAKEGVVAAAEKTKQGVTEAAEKTKEGVLYVGSKTREGVV-Q      50
sp|Q91ZZ3|SYUB_MOUSE                ---------MDVFMKGLSMAKEGVVAAAEKTKQGVTEAAEKTKEGVLYVGSKT-SGVV-Q      49
tr|A0A8C4TNP5|A0A8C4TNP5_ERPCA      ---------MDVLMKGLSKAKEGMAAAAEKTKEGVAVAAEKTKEGVLYVGNMTKEGVV-Q      50
tr|A0A7N9I9A4|A0A7N9I9A4_MACFA      GPATQAPDPGPIPTPAPSPPRPGLAEPPRP----PAAATVPSPAPAPHPGSKTREGVV-Q      55
tr|K7FSI4|K7FSI4_PELSI              ------------------------------------------------------------      0
tr|A0A8C4RPQ9|A0A8C4RPQ9_ERPCA      ---------MDVLMKGLSKAKEGVVAAAEKTKQGVAEAAGKTKEGVMYVGTKTKDG----      47
tr|Q7SZ02|Q7SZ02_XENLA              ---------MDVFMKGLSKAKEGVVAAAEKTKQGVAEAAGKTKEGVLYVGSKTKEGVV-H      50
sp|O55042|SYUA_MOUSE                ---------MDVFMKGLSKAKEGVVAAAEKTKQGVAEAAGKTKEGVLYVGSKTKEGVV-H      50
sp|P37840|SYUA_HUMAN                ---------MDVFMKGLSKAKEGVVAAAEKTKQGVAEAAGKTKEGVLYVGSKTKEGVV-H      50
tr|G7P5X2|G7P5X2_MACFA              ---------MDVFMKGLSKAKEGVVAAAEKTKQGVAEAAGKTKEGVLYVGSKTKEGVV-H      50
tr|Q9I9H1|Q9I9H1_CHICK              ---------MDVFMKGLNKAKEGVVAAAEKTKQGVAEAAGKTKEGVLYVGSRTKEGVV-H      50
tr|K7FW98|K7FW98_PELSI              ---------MDVFMKGLSKAKEGVVAAAEKTKQGMAEAAGKTKEGVLYVGSRTREGVV-H      50


tr|A0A8C4SHF3|A0A8C4SHF3_ERPCA      SVNAVAEKTKEQANIVGDAVVSSATQVSAKTVEGVENVAATGGLIKKDEQEGELVQNPAE      110
tr|Q7SYT8|Q7SYT8_XENLA              SVNTVAEKTKEQANVVGGAVVSGVNQVSSKTVEGTENVVSSTGLVKKEDLHPDQPEE-PA      109
tr|Q9I9H0|Q9I9H0_CHICK              SVTSVAEKTKEQANVVGEAVVASVNTVANKTVEGAETIVATTGVVKKEDLAPQQPAAE--      108
tr|K7FIG2|K7FIG2_PELSI              SVSSVAEKTKEQANMVGEAVVASVNTVAGKTVEGAENIVTTTGIVKKEELSHPEHPAEPA      110
XP_038599981.1                      SVSSVAEKTKEQASAVSEAMVASVNTVASKTVEGAESIVVTAGVVKKEDLLRPDQLEEAA      110
tr|A0A5F8G4U8|A0A5F8G4U8_MONDO      SVTSVAEKTKEQANLVSDVMVASVNTVANKTVEEAENIVVTSGIVRKEDLVNPAQPEHGA      110
sp|Q9Z0F7|SYUG_MOUSE                SVTSVAEKTKEQANAVSEAVVSSVNTVANKTVEEAENIVVTTGVVRKEDLEPPAQDQEAK      110
sp|O76070|SYUG_HUMAN                SVTSVAEKTKEQANAVSEAVVSSVNTVATKTVEEAENIAVTSGVVRKEDLRPSAPQQEGE      110
tr|G7PEV7|G7PEV7_MACFA              SVTSVAEKTKEQANAVSEAVVSSVNTVAAKTVEEAENIAVTSGVVRKEDLKPSAPQQEGE      110
tr|F6T1V6|F6T1V6_MONDO              GSGSVVRDQSNE--STEGITMGGIRGD------DGSSVGVGRVLVKSSQWWH-GIPEEVG      102
tr|Q6GQG2|Q6GQG2_XENLA              GVTSVAEKTKEQASHLGGAVMS-----------GAGNIAAATGLVKKDEFPTDLKPEEEA      99
XP_038626735.1                      SVASVAEKTKEQASQLGGAVFS-----------GAGNIAAATGLMKKEDLPADVKPEEVG      99
NP_990002.1                         GVTSVAEKAKEQASQLGEAAFS-----------GAGNIAAATGLVKKEEFPADLKAEEVA      98
sp|Q16143|SYUB_HUMAN                GVASVAEKTKEQASHLGGAVFS-----------GAGNIAAATGLVKREEFPTDLKPEEVA      99
sp|Q91ZZ3|SYUB_MOUSE                GVASVAEKTKEQASHLGGAVFS-----------GAGNIAAATGLVKKEEFPTDLKPEEVA      98
tr|A0A8C4TNP5|A0A8C4TNP5_ERPCA      GVASVAEKTKEQASQLGGAVFS-----------GAGNIAAATGLMKKEEFPTDIKPEELG      99
tr|A0A7N9I9A4|A0A7N9I9A4_MACFA      GVASVAEKTKEQASHLGGAVFS-----------GAGNIAAATGLVKREEFPTDLKPEEVA      104
tr|K7FSI4|K7FSI4_PELSI              ----VAEKTKEQASQLGGAVMS-----------GAGNIAAATGLVKKEEFPTDLKPEEVG      45
tr|A0A8C4RPQ9|A0A8C4RPQ9_ERPCA      -VTSVAEKTKEQVSNVGGAVVTGVTAVAHKTVEGAGNIAAATGLVKRDHLGKQN-EDMLS      105
tr|Q7SZ02|Q7SZ02_XENLA              GVTTVAEKTKEQVSNVGGAVVTGVTAVAHKTVEGAGNFAAATGLVKKDQK---N-ESGFG      106
sp|O55042|SYUA_MOUSE                GVTTVAEKTKEQVTNVGGAVVTGVTAVAQKTVEGAGNIAAATGFVKKDQMGK-G-EEGYP      108
sp|P37840|SYUA_HUMAN                GVATVAEKTKEQVTNVGGAVVTGVTAVAQKTVEGAGSIAAATGFVKKDQLGK-N-EEGAP      108
tr|G7P5X2|G7P5X2_MACFA              GVATVAEKTKEQVTNVGGAVVTGVTAVAQKTVEGAGSIAAATGFIKKDQLGK-N-EEGAP      108
tr|Q9I9H1|Q9I9H1_CHICK              GVTTVAEKTKEQVSNVGGAVVTGVTAVAQKTVEGAGNIAAATGLVKKDQLAKQN-EEGFL      109
tr|K7FW98|K7FW98_PELSI              GVTTMAEKTKEQVSNVGGAVVTGVTAVAHKTVEGAGNIAAATGLVKKDQMAKQN-EEGLS      109
                                        :... .::        .               ..     .:: ..

tr|A0A8C4SHF3|A0A8C4SHF3_ERPCA      NESEED---AQAAEQVGN------------------------------------------      125
tr|Q7SYT8|Q7SYT8_XENLA              AE-EPAVEATESIEQVGDGEN---------------------------------------      129
tr|Q9I9H0|Q9I9H0_CHICK              --GEAAIP--GSTEGGGEGENEGN------------------------------------      128
tr|K7FIG2|K7FIG2_PELSI              AAEEEPPA--EDIKATGEGESESN------------------------------------      132
XP_038599981.1                      AEENPAEAPAEVPEATEKEDNGGN------------------------------------      134
tr|A0A5F8G4U8|A0A5F8G4U8_MONDO      PEEQPAEAAEVT-------EEVGAPDAFFPLSPGLDRFSLFSPLLPASWASVTAAIRGAE      163
sp|Q9Z0F7|SYUG_MOUSE                EQEENE----EA-------KSGED------------------------------------      123
sp|O76070|SYUG_HUMAN                ASKEKEEVAEEA-------QSGGD------------------------------------      127
tr|G7PEV7|G7PEV7_MACFA              AAKEKEEVAEEA-------QSGGD------------------------------------      127
tr|F6T1V6|F6T1V6_MONDO              QEVGEEPMAEPLLDTEGESYEE----------PPQEEYQEYEPEA---------------      137
tr|Q6GQG2|Q6GQG2_XENLA              QEALEEPAAEPLLEPEGESYED-----------PQDDYQEYEPEA---------------      133
XP_038626735.1                      QEAGEEPLIEPLLEPEGESYED----------PTQEEYQEYEPEA---------------      134
NP_990002.1                         QEAVEEPLVEPLLEPEGESYEE----------SPQEEYQEYEPEA---------------      133
sp|Q16143|SYUB_HUMAN                QEAAEEPLIEPLMEPEGESYED----------PPQEEYQEYEPEA---------------      134
```

```
sp|Q91ZZ3|SYUB_MOUSE                QEAAEEPLIEPLMEPEGESYED----------SPQEEYQEYEPEA---------------       133
tr|A0A8C4TNP5|A0A8C4TNP5_ERPCA      QEAVEEPLGEPIMEPEGEAYEE----------APQDEYQEYEPEA---------------       134
tr|A0A7N9I9A4|A0A7N9I9A4_MACFA      QEAAEEPLIEPLMEPEGESYED----------PPQEEYQEYEPEA---------------       139
tr|K7FSI4|K7FSI4_PELSI              QEAGEEPLSEPLLEPEGEAYEE----------PPQ-------------------------       70
tr|A0A8C4RPQ9|A0A8C4RPQ9_ERPCA      QEGLMDNTADYPLDPD-DTYDM----------PPEDDCQEYQPNA---------------       139
tr|Q7SZ02|Q7SZ02_XENLA              PEGTMENSENMPVNPNNETYEM----------PPEEEYQDYDPEA---------------       141
sp|O55042|SYUA_MOUSE                QEGIL-E--DMPVDPGSEAYEM----------PSEEGYQDYEPEA---------------       140
sp|P37840|SYUA_HUMAN                QEGIL-E--DMPVDPDNEAYEM----------PSEEGYQDYEPEA---------------       140
tr|G7P5X2|G7P5X2_MACFA              QEGIL-Q--DMPVDPDNEAYEM----------PSEEGYQDYEPEA---------------       140
tr|Q9I9H1|Q9I9H1_CHICK              QEGMV-NNTDIPVDPENEAYEM----------PPEEEYQDYEPEA---------------       143
tr|K7FW98|K7FW98_PELSI              QEGMM-DSTDMPMDPDNEAYEM----------PPEEEYQDYEPEA---------------       143


tr|A0A8C4SHF3|A0A8C4SHF3_ERPCA      --------------------        125
tr|Q7SYT8|Q7SYT8_XENLA              --------------------        129
tr|Q9I9H0|Q9I9H0_CHICK              --------------------        128
tr|K7FIG2|K7FIG2_PELSI              --------------------        132
XP_038599981.1                      --------------------        134
tr|A0A5F8G4U8|A0A5F8G4U8_MONDO      EKGFPPCGLFSLLLSPFILP        183
sp|Q9Z0F7|SYUG_MOUSE                --------------------        123
sp|O76070|SYUG_HUMAN                --------------------        127
tr|G7PEV7|G7PEV7_MACFA              --------------------        127
tr|F6T1V6|F6T1V6_MONDO              --------------------        137
tr|Q6GQG2|Q6GQG2_XENLA              --------------------        133
XP_038626735.1                      --------------------        134
NP_990002.1                         --------------------        133
sp|Q16143|SYUB_HUMAN                --------------------        134
sp|Q91ZZ3|SYUB_MOUSE                --------------------        133
tr|A0A8C4TNP5|A0A8C4TNP5_ERPCA      --------------------        134
tr|A0A7N9I9A4|A0A7N9I9A4_MACFA      --------------------        139
tr|K7FSI4|K7FSI4_PELSI              --------------------        70
tr|A0A8C4RPQ9|A0A8C4RPQ9_ERPCA      --------------------        139
tr|Q7SZ02|Q7SZ02_XENLA              --------------------        141
sp|O55042|SYUA_MOUSE                --------------------        140
sp|P37840|SYUA_HUMAN                --------------------        140
tr|G7P5X2|G7P5X2_MACFA              --------------------        140
tr|Q9I9H1|Q9I9H1_CHICK              --------------------        143
tr|K7FW98|K7FW98_PELSI              --------------------        143
```

```
#  Percent Identity  Matrix - created by Clustal2.1
#
#
```

1: tr|A0A8C4SHF3|A0A8C4SHF3_ERPCA	100.00	69.11	64.17	62.90	57.60	57.98	61.86	61.34	63.87	35.34	59.65	56.14	55.75	58.77	59.29	54.39	32.73	45.00	57.98	58.68	57.38	57.38	58.20	56.10	54.47
2: tr|Q7SYT8|Q7SYT8_XENLA	69.11	100.00	76.42	73.23	66.67	67.21	68.64	68.03	68.03	40.00	61.02	61.02	57.26	61.86	62.39	55.08	34.21	43.75	59.35	60.80	61.29	61.29	60.48	59.84	57.48
3: tr|Q9I9H0|Q9I9H0_CHICK	64.17	76.42	100.00	78.12	74.22	74.38	78.15	75.21	75.21	37.61	61.74	61.74	62.28	61.74	62.28	57.39	34.23	44.26	61.67	60.66	62.30	61.48	60.66	61.29	58.87
4: tr|K7FIG2|K7FIG2_PELSI	62.90	73.23	78.12	100.00	72.73	72.00	73.98	71.20	72.00	39.67	62.18	59.66	60.17	62.18	62.71	57.14	35.65	46.15	56.45	57.94	58.73	58.73	57.94	57.03	54.69
5: XP_038599981.1	57.60	66.67	74.22	72.73	100.00	75.59	75.61	70.87	70.87	34.96	55.37	54.55	54.17	55.37	55.83	49.59	30.77	34.33	53.17	52.34	54.33	56.69	55.12	52.31	50.00
6: tr|A0A5F8G4U8|A0A5F8G4U8_MONDO	57.98	67.21	74.38	72.00	75.59	100.00	78.05	75.59	75.59	35.66	54.76	51.97	50.00	52.76	52.38	47.24	29.27	34.92	51.88	51.49	53.38	52.63	51.13	50.74	48.53
7: sp|Q9Z0F7|SYUG_MOUSE	61.86	68.64	78.15	73.98	75.61	78.05	100.00	86.99	86.99	35.71	58.18	58.18	56.88	58.18	58.72	52.73	31.13	39.29	56.90	57.26	56.78	56.78	55.93	55.46	52.94
8: sp|O76070|SYUG_HUMAN	61.34	68.03	75.21	71.20	70.87	75.59	86.99	100.00	96.06	36.21	57.89	57.02	55.75	57.02	57.52	51.75	30.91	38.33	55.00	55.37	56.67	55.83	54.17	53.66	51.22
9: tr|G7PEV7|G7PEV7_MACFA	63.87	68.03	75.21	72.00	70.87	75.59	86.99	96.06	100.00	36.21	57.02	56.14	54.87	56.14	56.64	50.88	30.00	38.33	55.83	56.20	57.50	56.67	55.00	54.47	52.03
10: tr|F6T1V6|F6T1V6_MONDO	35.34	40.00	37.61	39.67	34.96	35.66	35.71	36.21	36.21	100.00	57.69	60.31	57.69	61.07	60.77	51.15	34.65	44.78	42.31	46.62	46.97	46.97	46.21	47.76	47.01
11: tr|Q6GQG2|Q6GQG2_XENLA	59.65	61.02	61.74	62.18	55.37	54.76	58.18	57.89	57.02	57.69	100.00	81.20	85.61	87.97	88.64	81.20	65.12	86.96	67.72	67.44	67.97	66.41	65.62	67.94	67.18
12: XP_038626735.1	56.14	61.02	61.74	59.66	54.55	51.97	58.18	57.02	56.14	60.31	81.20	100.00	81.95	88.06	87.22	80.60	63.08	85.71	58.59	63.85	63.57	64.34	64.34	67.42	64.39
13: NP_990002.1	55.75	57.26	62.28	60.17	54.17	50.00	56.88	55.75	54.87	57.69	85.61	81.95	100.00	87.22	88.72	81.20	64.34	84.29	60.63	66.67	66.41	64.84	64.06	67.18	65.65
14: sp|Q16143|SYUB_HUMAN	58.77	61.86	61.74	62.18	55.37	52.76	58.18	57.02	56.14	61.07	87.97	88.06	87.22	100.00	97.74	82.84	74.62	87.14	61.72	67.69	67.44	67.44	66.67	68.18	68.18
15: sp|Q91ZZ3|SYUB_MOUSE	59.29	62.39	62.28	62.71	55.83	52.38	58.72	57.52	56.64	60.77	88.64	87.22	88.72	97.74	100.00	83.46	72.09	87.14	60.63	67.44	67.19	67.19	66.41	67.94	67.18
16: tr|A0A8C4TNP5|A0A8C4TNP5_ERPCA	54.39	55.08	57.39	57.14	49.59	47.24	52.73	51.75	50.88	51.15	81.20	80.60	81.20	82.84	83.46	100.00	63.85	87.14	60.16	63.85	63.57	63.57	63.57	65.15	64.39
17: tr|A0A7N9I9A4|A0A7N9I9A4_MACFA	32.73	34.21	34.23	35.65	30.77	29.27	31.13	30.91	30.00	34.65	65.12	63.08	64.34	74.62	72.09	63.85	100.00	87.14	38.71	43.65	43.20	43.20	42.40	44.53	45.31
18: tr|K7FSI4|K7FSI4_PELSI	45.00	43.75	44.26	46.15	34.33	34.92	39.29	38.33	38.33	44.78	86.96	85.71	84.29	87.14	87.14	87.14	87.14	100.00	48.53	53.03	52.31	50.77	49.23	55.88	52.94
19: tr|A0A8C4RPQ9|A0A8C4RPQ9_ERPCA	57.98	59.35	61.67	56.45	53.17	51.88	56.90	55.00	55.83	42.31	67.72	58.59	60.63	61.72	60.63	60.16	38.71	48.53	100.00	77.21	76.30	77.04	76.30	77.54	78.26
20: tr|Q7SZ02|Q7SZ02_XENLA	58.68	60.80	60.66	57.94	52.34	51.49	57.26	55.37	56.20	46.62	67.44	63.85	66.67	67.69	67.44	63.85	43.65	53.03	77.21	100.00	84.78	84.78	84.06	87.14	86.43
21: sp|O55042|SYUA_MOUSE	57.38	61.29	62.30	58.73	54.33	53.38	56.78	56.67	57.50	46.97	67.97	63.57	66.41	67.44	67.19	63.57	43.20	52.31	76.30	84.78	100.00	95.00	93.57	87.86	86.43
22: sp|P37840|SYUA_HUMAN	57.38	61.29	61.48	58.73	56.69	52.63	56.78	55.83	56.67	46.97	66.41	64.34	64.84	67.44	67.19	63.57	43.20	50.77	77.04	84.78	95.00	100.00	98.57	88.57	86.43
23: tr|G7P5X2|G7P5X2_MACFA	58.20	60.48	60.66	57.94	55.12	51.13	55.93	54.17	55.00	46.21	65.62	64.34	64.06	66.67	66.41	63.57	42.40	49.23	76.30	84.06	93.57	98.57	100.00	87.86	85.71
24: tr|Q9I9H1|Q9I9H1_CHICK	56.10	59.84	61.29	57.03	52.31	50.74	55.46	53.66	54.47	47.76	67.94	67.42	67.18	68.18	67.94	65.15	44.53	55.88	77.54	87.14	87.86	88.57	87.86	100.00	90.21
25: tr|K7FW98|K7FW98_PELSI	54.47	57.48	58.87	54.69	50.00	48.53	52.94	51.22	52.03	47.01	67.18	64.39	65.65	68.18	67.18	64.39	45.31	52.94	78.26	86.43	86.43	86.43	85.71	90.21	100.00
